# Supplementary material for: Harnessing cholesterol uptake of malaria parasites for therapeutic applications
Source: EMBO Mol Med. 2024 Jun 11;16(7):4. doi: 10.1038/s44321-024-00087-1 (PMC11251039; doi:10.1038/s44321-024-00087-1)
Supplement: Supplementary file 1 — Appendix [file 44321_2024_87_MOESM1_ESM.pdf]

Appendix
Fraser/Curtis et al.

Table of Content:

Chemical Synthesis	Page 1-24
Biological Methods	Page 25-34
References	Page 35-38

Chemical synthesis

Experimental

Unless otherwise specified ^{1}H NMR and ^{13}C NMR experiments were performed using deuterated chloroform ($CDCl_3$), deuterated dichloromethane (CD_2Cl_2), deuterated dimethtlsulfoxide (DMSO-d_6), deuterated dioxane (dioxane-d_8) or deuterated methanol (MeOD-d_4) using Bruker Avance 400 MHz, 600 MHz or 700 MHz spectrometers at 298 K. Deuterated solvents were supplied by Cambridge Isotope Laboratories, Inc. Residual solvent peaks or ^{13}C signals corresponding to deuterated solvent were used as internal reference corresponding to values given by Fulmer *et al.*, (2010) *(1)*. Analysis of these spectra was completed using MestReNOVA (version 14.2.1). Chemical shifts are reported in parts per million (ppm). Multiplicity is assigned as s = singlet, d = doublet, t = triplet, q = quartet, sept = septet and m = multiplet or combination of these. Where compounds were found as a pair of inseparable diastereomers; peaks corresponding to the same ^{1}H and ^{13}C environments are given as peak 1/peak 2. Coupling constants (*J*) reported in Hz. Unless otherwise stated, low-resolution mass spectrometry (LRMS) was performed using a Waters LCT Premier XE mass spectrometer and high-resolution mass spectrometry (HRMS) was performed using a Waters SYNAPT G2-Si mass spectrometer. Samples were prepared at a concentration of ~1 mg of analyte in 1 mL of methanol for LRMS and was subsequently diluted in methanol for HRMS. Infrared spectra were recorded using PerkinElmer 1800 Series FTIR spectrometer. Specific rotation recorded using the Rudolf research systems Autopol I polarimeter, where 10 mg of analyte was dissolved in 1.0 mL of chloroform. Thin layer chromatography (TLC) analysis performed using Merck TLC silica gel 60 F254 plates using mobile phases as stated. Purification by silica flash chromatography was conducted using chem-supply silica gel 60 0.04 – 0.06 mm (230 – 400 mesh ASTM) using eluent as stated. Purification by high performance liquid chromatography (HPLC) was conducted using Waters 2695 separations module, Agilent Pursuit XRs 5 C18 250x10mm column, Waters 2998 Photodiode array detector (266 nm) and Waters Fraction Collector III, controlled by Waters Empower 2 software.

Purity of compounds used for biological testing (>95%) was determined using the same Waters separations module and photodiode array detector with an Agilent Eclipse XDB-C18 5 μm column. General eluent used: 95% HPLC methanol (Honeywell - Burdick & Jackson) : 5% (0.1% HPLC trifluoroacetic acid) (Sigma-Aldrich) solution in filtered water or 90% Acetonitrile : 10% water.

Specific transformations

Scheme A)

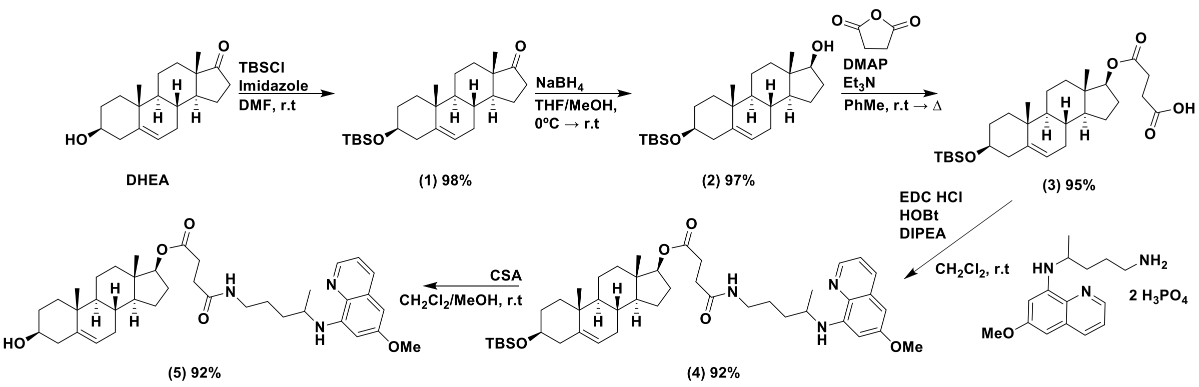

3β-(*tert*-Butyldimethylsilyloxy)-androst-5-en-17-one (1)

Procedure adapted from Yamauchi *et al.,* (2005) *(2)*. *tert*-Butyldimethylsilyl chloride (2.74 g, 18.2 mmol) was added to a stirring solution of 3β-hydroxyandrost-5-en-17-one (3.50 g, 12.1 mmol) and imidazole (2.07 g, 30.3 mmol) in anhydrous DMF (40 mL). The reaction mixture was stirred at room temperature for 2 hours. The reaction was diluted with EtOAc (100 mL) and 5% citric acid solution (100 mL). The aqueous layer was further extracted with EtOAc (2 x 100 mL). The combined organic extract was washed with saturated NaCl solution, dried over anhydrous $MgSO_4$ and filtered. The solvent was removed under reduced pressure and the residue obtained was purified by silica column chromatography (20% EtOAc : n-hexanes) to yield 3β-(*tert*-butyldimethylsilyloxy)-androst-5-en-17-one (4.79 g, 98%) as a white solid.

m.p 147-149 °C (lit.[2] 149 °C); **^{1}H NMR** (400 MHz, $CDCl_3$) δ 5.34 (d, *J* = 5.3 Hz, 1H), 3.48 (m, 1H), 2.40 – 0.95 (m,19H), 1.02 (s, 3H), 0.89 (s, 9H), 0.88 (s, 3H), 0.06 (s, 6H); **^{13}C NMR** (101 MHz, $CDCl_3$) δ 221.3, 141.9, 120.5, 72.6, 52.0, 50.5, 47.7, 42.9, 37.5, 36.9, 36.0, 32.2,

31.7, 31.6, 31.0, 26.1, 22.0, 20.5, 19.6, 18.4, 13.7, -4.4 (2C); **LRMS** (GCMS) found *m/z* 345 $[M-C_4H_9]^+$; **HRMS** (ESI +): found *m/z* 425.2834 $[M+Na]^+$, theoretical ($C_{25}H_{42}O_2SiNa$) *m/z* 425.2846 $[M+Na]^+$; **IR** 2946, 2857, 1746 cm^{-1}; **specific rotation** $[\alpha]_D^{25}$ -38.63 (c 1.0, $CHCl_3$).

3β-(*tert*-Butyldimethylsilyloxy)-androst-5-en-17β-ol (2)

Procedure adapted from Moreira *et al.,* (2008) *(3)*. Sodium borohydride (0.536 g, 14.2 mmol) was added to a stirring solution of 3β-(*tert*-butyldimethylsilyloxy)-androst-5-en-17-one (2.85 g, 7.08 mmol) in ice bath cooled THF (20 mL) and MeOH (20 mL). The reaction mixture was brought to room temperature and stirred for 2.5 hours. The reaction was diluted with CH_2Cl_2 (40 mL) and water (20 mL), then treated with 2M HCl solution (5 mL). The aqueous layer was further extracted with CH_2Cl_2 (2 x 50 mL). The combined organic extract was washed with saturated NaCl solution, dried over anhydrous $MgSO_4$ and filtered. The solvent was removed under reduced pressure and the residue obtained was purified by silica column chromatography (20% EtOAc : n-hexanes) to yield 3β-(*tert*-butyldimethylsilyloxy)-androst-5-en-17β-ol (2.78 g, 97%) as a white solid.

m.p 170-172 °C (lit. *(4)* 171-172 °C); **^{1}H NMR** (400 MHz, $CDCl_3$) δ 5.31 (d, *J* = 4.6 Hz, 1H), 3.64 (t, *J* = 8.5 Hz, 1H), 3.52 – 3.42 (m, 1H), 2.30 – 0.93 (m, 19H), 1.01 (s, 3H), 0.89 (s, 9H), 0.75 (s, 3H), 0.05 (s, 6H), OH not observed; **^{13}C NMR** (101 MHz, $CDCl_3$) δ 141.8, 121.0, 82.1, 72.7, 51.5, 50.5, 43.0, 42.9, 37.6, 36.8, 36.8, 32.2, 32.1, 31.7, 30.7, 26.1, 23.6, 20.8, 19.6, 18.4, 11.1, -4.4 (2C); **LRMS** (GCMS) found *m/z* 347 $[M-C_4H_9]^+$; **HRMS** (ESI+) found *m/z* 427.2992 $[M+Na]^+$, theoretical ($C_{25}H_{44}O_2SiNa$) *m/z* 427.3003 $[M+Na]^+$; **IR** 3305, 2929, 2853 cm^{-1}; **specific rotation** $[\alpha]_D^{25}$ -38.63 (c 1.0, $CHCl_3$)

3β-(*tert*-Butyldimethylsilyloxy)-androst-5-en-17β-yl hemisuccinate (3)

Procedure adapted from Keglevich *et al.,* (2019) *(5)*. Succinic anhydride (0.317 g, 3.17 mmol) was added to a stirring solution of 3β-(*tert*-butyldimethylsilyloxy)-androst-5-en-17*β*-ol (0.640 g, 1.58 mmol), Et_3N (1 mL) and DMAP 0.194 g, 1.58 mmol) in anhydrous toluene (20 mL). The reaction mixture was brought to reflux and stirred for 24 hours. The reaction was diluted with CH_2Cl_2 (40 mL), water (20 mL), then treated with 2M HCl solution until pH 2. The aqueous layer was further extracted with CH_2Cl_2 (2 x 40 mL). The combined organic extract was washed with saturated NaCl solution, dried over anhydrous $MgSO_4$ and filtered. The solvent was removed under reduced pressure and the residue obtained was purified by silica column chromatography (5% AcOH : 20% EtOAc : n-hexanes) to yield 3β-(*tert*-butyldimethylsilyloxy)-androst-5-en-17β-yl hemisuccinate (0.763 g, 95%) as a white solid.

m.p 164-168 °C; **^{1}H NMR** (400 MHz, $CDCl_3$) δ 5.31 (d, *J* = 4.3 Hz, 1H), 4.62 (t, *J* = 8.4 Hz, 1H), 3.47 (m, 1H), 2.72 – 2.59 (m, 4H), 2.30 – 1.05 (m, 19H) 1.00 (s, 3H), 0.88 (s, 9H), 0.79 (s, 3H), 0.05 (s, 6H), COOH not observed; **^{13}C NMR** (101 MHz, $CDCl_3$) δ 177.9, 172.2, 141.8, 120.9, 83.4, 72.7, 51.2, 50.3, 42.9, 42.6, 37.5, 36.9, 36.8, 32.2, 31.9, 31.6, 29.3, 29.2, 27.6, 26.1, 23.7, 20.7, 19.6, 18.4, 12.1, -4.4 (2C); **LRMS** (ESI-) found *m/z* 503.2 $[M-H]^-$; **HRMS** (ESI-) found *m/z* 503.3192 $[M-H]^-$, theoretical ($C_{29}H_{47}O_5Si$) *m/z* 503.3198 $[M-H]^-$; **IR** 3000, 2964, 1735, 1705 cm^{-1}; **specific rotation** $[\alpha]_D^{25}$ -10.30 (c 1.0, $CHCl_3$).

3β-(*tert*-Butyldimethylsilyloxy)-androst-5-en-17β-yl succinate-primaquine amide (4)

Procedure adapted from Fernandes *et al.,* (2009) *(6)*. EDC HCl (0.189 g, 0.992 mmol) was added to a stirring solution of 3β-(*tert*-butyldimethylsilyloxy)-androst-5-en-17β-yl hemisuccinate (0.250 g, 0.496 mmol), HOBt (67 mg, 0.05 mmol) and DIPEA (0.75 mL) in anhydrous CH_2Cl_2 (5 mL). Separate to this, primaquine bisphosphate (0.248 g, 0.546 mmol) was added to a solution of DIPEA (0.75 mL) in CH_2Cl_2 (5 mL) and was stirred until dissolved. After 30 minutes, the two solutions were combined, and then stirred at room temperature for 24 hours. The reaction was diluted with CH_2Cl_2 (40 mL) and 5% citric acid solution (40 mL). The aqueous layer was further extracted with CH_2Cl_2 (2 x 40 mL). The combined organic extract was washed with saturated $NaHCO_3$ solution and then saturated NaCl solution, dried over anhydrous Na_2SO_4 and filtered. The solvent was removed under reduced pressure and the residue obtained was purified by silica column chromatography (3% MeOH : CH_2Cl_2) to yield 3β-(*tert*-butyldimethylsilyloxy)-androst-5-en-17β-yl succinate-primaquine amide (288 mg, 92%) as a green solid.

m.p 94-96 °C; **^{1}H NMR** (400 MHz, CD_2Cl_2) δ 8.50 (d, *J* = 4.1 Hz, 1H), 7.94 (d, *J* = 8.2 Hz, 1H), 7.31 (dd, *J* = 8.2, 4.2 Hz, 1H), 6.35 (d, *J* = 2.2 Hz, 1H), 6.27 (d, *J* = 2.2 Hz, 1H), 6.03 (d, *J* = 8.4 Hz, 1H), 5.69 (s, 1H), 5.30 (s, 1H), 4.56 (t, *J* = 8.4 Hz, 1H), 3.87 (s, 3H), 3.63 (s, 1H), 3.47 (tt, *J* = 10.4, 4.7 Hz, 1H), 3.29 – 3.17 (m, 2H), 2.58 (t, *J* = 6.7 Hz, 2H), 2.38 (t, *J* = 6.8 Hz, 2H), 2.27 – 0.88 (m, 23H), 1.28 (d, *J* = 6.4 Hz, 3H), 0.99 (s, 3H), 0.88 (s, 9H), 0.77 (s, 3H), 0.05 (s, 6H); **^{13}C NMR** (101 MHz, CD_2Cl_2) δ 173.2, 171.5, 159.9, 145.4, 144.7, 142.0, 135.7, 135.1, 130.3, 122.3, 121.1, 97.0, 92.0, 83.3, 72.9, 55.5, 51.4, 50.6, 48.2, 43.2, 42.8, 39.8, 37.7, 37.2, 37.0, 34.4, 32.5, 32.1, 31.9, 31.5, 30.2, 27.9, 26.8, 26.1, 23.9, 21.0, 20.7, 19.6, 18.4, 12.1, -4.5 (2C); **LRMS** (ESI+) found m/z 746.5 $[M+H]^+$; **HRMS** (ESI+) found m/z 746.4929

[M+H]$^+$, theoretical ($C_{44}H_{68}N_3O_5$) m/z 746.4923 [M+H]$^+$; **IR** 3379, 3314, 2929, 2854, 1731, 1648, 1615, 1595, 1576, 1519 cm^{-1}.

3β-Hydroxyandrost-5-en-17β-yl succinate-primaquine amide (5)

Procedure adapted from Yamashita *et al.,* (2005) *(7)*. Camphor sulfonic acid (0.155 g, 6.66 mmol) was added to a stirring solution of 3β-(*tert*-butyldimethylsilyloxy)-androst-5-en-17β-yl succinate-primaquine amide (284 mg, 0.333 mmol) in CH_2Cl_2. (7.5 mL) and MeOH (7.5 mL). The reaction mixture was stirred at room temperature for 3 hours. The reaction was diluted with saturated $NaHCO_3$ solution (30 mL) and extracted with CH_2Cl_2 (20 mL). The aqueous layer was further extracted with CH_2Cl_2 (2 x 30 mL). The combined organic extract was washed with saturated NaCl solution, dried over anhydrous Na_2SO_4 and filtered. The solvent was removed under reduced pressure and the residue obtained was purified by silica column chromatography (3% MeOH : CH_2Cl_2) to yield 3β-hydroxyandrost-5-en-17β-yl succinate-primaquine amide (194 mg, 92%) as a green solid.

m.p 78-80 °C **^{1}H NMR** (400 MHz, MeOD) δ 8.55 – 8.48 (m, 1H), 8.06 (d, *J* = 8.2 Hz, 1H), 8.01 – 7.94 (m, 1H), 7.42 – 7.35 (m, 1H), 6.51 – 6.44 (m, 1H,), 6.35 (s, 1H), 5.32 (d, *J* = 4.6 Hz, 1H), 4.56 – 4.48 (m, 1H), 3.87 (s, 3H), 3.70 – 3.59 (m, 1H), 3.44 – 3.35 (m, 1H), 3.26 – 3.15 (m, 2H), 2.59 (t, *J* = 6.0 Hz, 2H), 2.45 (t, *J* = 6.7 Hz, 2H), 2.27 – 0.77 (m, 23H), 1.28 (m, 3H), 0.98/0.96 (m, 3H), 0.77/0.76 (m, 3H), OH and aniline NH not observed; **^{13}C NMR** (101 MHz, MeOD) δ 174.18/174.17, 174.1, 161.0, 146.2, 145.3, 142.2, 136.5, 136.3, 131.6, 122.9, 122.1, 98.35/98.33, 93.0, 84.3, 72.3, 55.7, 52.16/52.11, 51.49/51.46, 48.6, 43.6/43.59, 43.0, 40.4, 38.4, 37.94/37.93, 37.7, 35.00/34.99, 32.9, 32.4, 32.3, 31.6, 30.69/30.68, 28.4,

27.22/27.20, 24.4, 21.6, 20.7, 19.9, 12.4; **LRMS** (ESI+) found *m/z* 632.4 $[M+H]^+$; **HRMS** found *m/z* 632.4061 $[M+H]^+$, theoretical ($C_{38}H_{54}N_3O_5$) *m/z* 632.4058 $[M+H]^+$; IR 3316, 2932, 1728, 1651, 1615, 1575, 1519 cm^{-1}.

Scheme B)

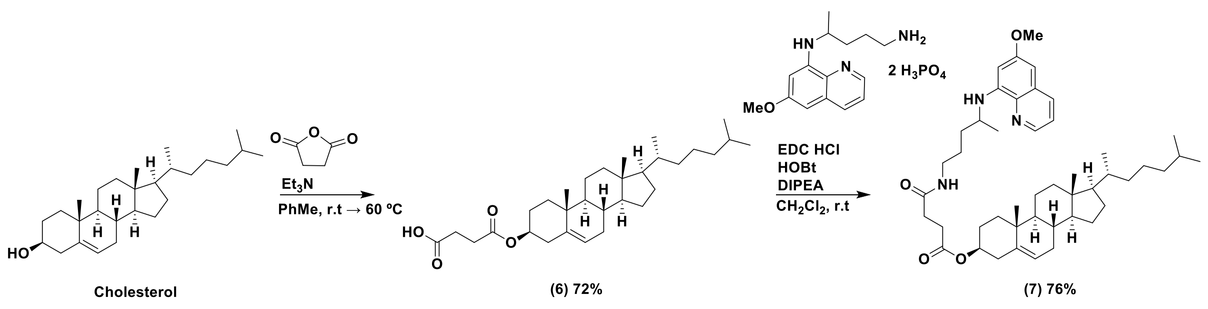

Cholester-3-yl hemisuccinate (6)[8]

Procedure adapted from Kumar *et al.*, (2016) *(8)*. Cholesterol (773.4 mg, 2 mmol) and succinic anhydride (320.1 mg, 3.2 mmol) were dissolved in dry toluene (4 mL). Triethylamine (70 µL, 0.5 mmol) was added to the suspension and the reaction mixture was stirred at 60 °C overnight. The reaction mixture was cooled down to ambient temperature and the pale-yellow suspension was treated with water (10 mL) and the organic layer extracted with dichloromethane (2 x 20 mL). The combined organic extracts were washed with 2 M hydrochloric acid (3 x 5 mL), water (3 x 5 mL) and saturated brine solution (8 mL) before being dried over anhydrous Na_2SO_4. The solution was then dried in vacuo to afford cholester-3-yl hemisuccinate (758.2 mg, 72 %) as a white solid.

[1]**H NMR** (400 MHz, $CDCl_3$) δ 5.37 (d, *J* 5.1 Hz, 1H), 4.63 (m, 1H), 2.64 (m, 4H), 2.32 (d, *J* 8.0 Hz, 2H), 2.03 – 1.76 (m, 5H), 1.62 – 1.02 (m, 18H), 1.00 (s, 3H), 0.98 – 0.92 (m, 3H), 0.89 (d, *J* 6.5 Hz, 3H), 0.84 (dd, *J* 6.6, 1.8 Hz, 6H), 0.65 (s, 3H), COOH not observed; **LRMS** (ESI+) found *m/z* 509 $[M+Na]^+$; **IR** (ATR, solid) 2937, 1707, 1465, 1435, 1377, 1316, 1247, 1180, 1000, 942, 799, 736, 655 cm^{-1}.

Cholester-3-yl succinate-primaquine amide (7)

Procedure adapted from Fernandes *et al.,* (2009) *(6)*. EDC HCl (39 mg, 0.21 mmol) was added to a stirring solution of cholester-3-yl hemisuccinate (50 mg, 0.10 mmol), HOBt (21 mg, 0.15 mmol) and DIPEA (0.5 mL) in anhydrous CH_2Cl_2 (5 mL). Separate to this, primaquine bisphosphate (51 mg, 0.11 mmol) was added to a solution of DIPEA (0.5 mL) in CH_2Cl_2 (5 mL) and was stirred until dissolved. After 30 minutes, the two solutions were combined and stirred at room temperature for 24 hours. The reaction mixture was diluted with CH_2Cl_2 (20 mL), water (20 mL) and 5% citric acid solution until pH 5. The aqueous layer was further extracted with CH_2Cl_2 (2 x 30 mL). The combined organic extract was washed with saturated $NaHCO_3$ solution and saturated NaCl solution, dried over anhydrous Na_2SO_4 and filtered. The solvent was removed under reduced pressure and the residue obtained was purified by silica column chromatography (3% MeOH : CH_2Cl_2) to yield cholester-3-yl succinate-primaquine amide (57 mg, 76%) as a green oil.

[1]**H NMR** (400 MHz, CD_2Cl_2) δ 8.50 (dd, *J* = 4.2, 1.6 Hz, 1H), 7.94 (dd, *J* = 8.2, 1.6 Hz, 1H), 7.31 (dd, *J* = 8.3, 4.2 Hz, 1H), 6.35 (d, *J* = 2.5 Hz, 1H), 6.27 (d, *J* = 2.5 Hz, 1H), 6.02 (d, *J* = 8.5 Hz, 1H), 5.83 – 5.72 (m, 1H), 5.35 (d, *J* = 5.0 Hz, 1H), 4.61 – 4.49 (m, 1H), 3.87 (s, 3H), 3.66 – 3.58 (m, 1H), 3.30 – 3.16 (m, 2H), 2.56 (t, *J* = 6.9 Hz, 2H), 2.38 (t, *J* = 6.8 Hz, 2H), 2.28

(d, J = 7.6 Hz, 2H), 2.04 – 0.83 (m, 30H), 1.28 (d, J = 6.3 Hz, 3H), 1.00 (s, 3H), 0.92 (d, J = 6.5 Hz, 3H), 0.87 (dd, J = 6.7, 1.6 Hz, 6H), 0.68 (s, 3H); **^{13}C NMR** (101 MHz, CD_2Cl_2) δ 172.7, 171.6, 159.9, 145.4, 144.7, 140.2, 135.7, 135.1, 130.3, 122.9, 122.3, 97.0, 92.0, 74.6, 57.1, 56.6, 55.5, 50.8, 48.2, 42.7, 40.2, 39.9, 39.8, 38.5, 37.4, 37.0, 36.6, 36.2, 34.3, 32.3, 32.3, 31.4, 30.3, 28.6, 28.4, 28.1, 26.8, 24.6, 24.2, 23.0, 22.7, 21.4, 20.7, 19.5, 18.9, 12.0; **LRMS** (ESI+) found *m/z* 728.6 $[M+H]^+$; **HRMS** (ESI+) found *m/z* 728.5364 $[M+H]^+$, theoretical ($C_{46}H_{70}N_3O_4$) *m/z* 728.5361 $[M+H]^+$; **IR** 3379, 2936, 2463, 1732, 1635, 1618, 1577, 1520 cm^{-1}.

Scheme C)

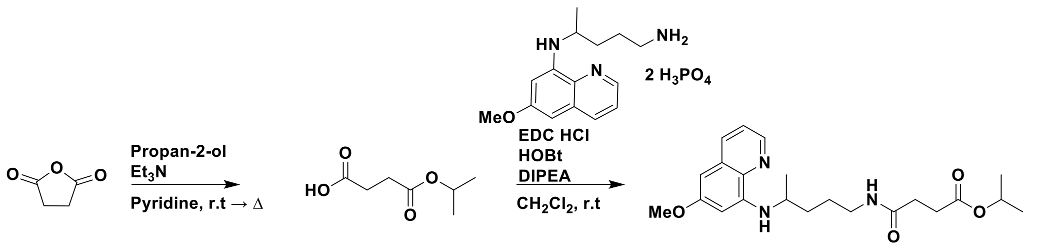

Propan-2-yl succinate (8)

Procedure adapted from Cole et al., (2017) *(9)*. Succinic anhydride (2.00 g, 20.0 mmol) was added to a stirring solution of pyridine (2.0 mL, 25 mmol) in propan-2-ol (20 mL). The solution was heated to reflux and stirred for 20 h. The reaction mixture was concentrated under reduced pressure. The residue obtained was partitioned between CH_2Cl_2 (30 mL) and water (40 mL) and treated with 2M HCl solution until pH 2. The aqueous layer was further extracted with CH_2Cl_2 (2 x 30 mL). The combined organic extract was washed with saturated NaCl solution

(60 mL), dried over anhydrous $MgSO_4$ and filtered. The solvent was removed under reduced pressure and the residue obtained was purified by silica column chromatography (20 % EtOAc : n-hexanes) to yield propan-2-yl succinate (2.38 g, 74%) as a white oily solid.

m.p 52-53 °C; **^{1}H NMR** (400 MHz, $CDCl_3$) δ 11.09 (s, 1H), 5.02 (hept, *J* = 6.3 Hz, 1H), 2.67 (t, *J* = 6.5 Hz, 2H), 2.58 (t, *J* = 6.5 Hz, 2H), 1.23 (d, *J* = 6.3 Hz, 6H); **^{13}C NMR** (101 MHz, $CDCl_3$) δ 178.3, 171.6, 68.3, 29.2, 29.0, 21.7; **LRMS** (ESI-) found *m/z* 159.1 $[M-H]^-$; **HRMS** (ESI-) found *m/z* 159.0658 $[M-H]^-$, theoretical ($C_7H_{11}O_4$) *m/z* 159.0663 $[M-H]^-$; **IR** 3124, 1726, 1713 cm^{-1}.

Propan-2-yl succinate-primaquine amide (9)

Procedure adapted from Fernandes *et al.,* (2009) *(6)*. EDC HCl (0.191 g, 1 mmol) was added to a stirring solution of propan-2-yl succinate (80 mg, 0.50 mmol), HOBt (68 mg, 0.5 mmol) and DIPEA (0.65 mL) in anhydrous CH_2Cl_2 (5 mL). Separate to this, primaquine bisphosphate (0.250 g, 0.550 mmol) was added to a solution of DIPEA (0.65 mL) in CH_2Cl_2 (5 mL) and was stirred until dissolved. After 30 minutes, the two solutions were combined, and then stirred at room temperature for 24 hours. The reaction mixture was diluted with CH_2Cl_2 (40 mL) and 5% citric acid solution (30 mL). The aqueous layer was further extracted with CH_2Cl_2 (2 x 40 mL). The combined organic extract was washed with saturated $NaHCO_3$ solution and saturated NaCl solution, dried over anhydrous Na_2SO_4 and filtered. The solvent was removed under reduced pressure and the residue obtained was purified by silica column chromatography (3% MeOH : CH_2Cl_2) to yield propan-2-yl succinate-primaquine amide (167 mg, 83%) as a green oil.

^{1}H NMR (400 MHz, MeOD) δ 8.48 (d, *J* = 4.2 Hz, 1H), 8.01 (d, *J* = 8.2 Hz, 1H), 7.37 – 7.32 (m, 1H), 6.47 – 6.41 (m, 1H), 6.33 – 6.28 (m, 1H), 4.94 (dq, *J* = 12.5, 6.3 Hz, 1H), 3.86 (s, 3H), 3.69 – 3.59 (m, 1H), 3.23 – 3.13 (m, 2H), 2.54 (t, *J* = 6.8 Hz, 2H), 2.43 (t, *J* = 6.9 Hz, 2H), 1.74 – 1.58 (m, 4H), 1.28 (d, *J* = 6.3 Hz, 3H), 1.18 (d, *J* = 6.3 Hz, 6H); **^{13}C NMR** (101 MHz, MeOD) δ 174.3, 173.8, 161.0, 146.2, 145.3, 136.5, 136.3, 131.6, 122.9, 98.3, 93.0, 69.2, 55.6, 48.9, 40.3, 34.9, 31.5, 30.8, 27.1, 22.0, 20.8; **LRMS** (ESI+) found *m/z* 424.2 $[M+Na]^+$; **HRMS** (ESI+) found *m/z* 402.2387 $[M+H]^+$, theoretical ($C_{22}H_{32}N_3O_4$) *m/z* 402.2387 $[M+H]^+$; **IR** 3390, 2981, 2863, 2472, 2414, 1727, 1634, 1615, 1594, 1576, 1518 cm^{-1}.

Scheme D)

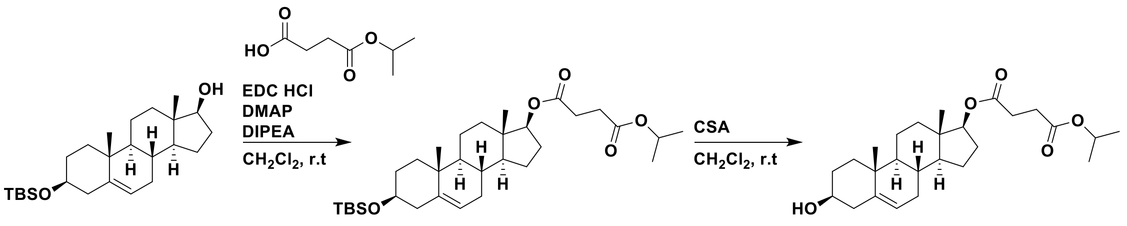

(2) (10) 81% (11) 94%

3β-(*tert*-Butyldimethylsilyloxy)-androst-5-en-17β-yl -propan-2-yl succinate diester (10)

Procedure adapted from Krausova *et al.,* (2018) *(10)*. EDC HCl (0.335 g, 1.74 mmol) was added to a stirring solution of propan-2-yl succinate (0.279 g, 1.74 mmol), DMAP (0.213 g, 1.74 mmol) and DIPEA (0.5 mL) in CH_2Cl_2 (15 mL). After 15 minutes 3β-(*tert*-butyldimethylsilyloxy)-androst-5-en-17β-ol (0.353 g, 0.874 mmol) was added to the solution. The reaction mixture was stirred at room temperature for 21 hours. The reaction was diluted

with CH_2Cl_2 (40 mL) and 5% citric acid solution (50 mL). The aqueous layer was further extracted with CH_2Cl_2 (2 x 40mL). The combined organic extract was washed with saturated NaCl solution, dried over anhydrous Na_2SO_4 and filtered. The solvent was removed under reduced pressure and the residue obtained was purified by silica column chromatography (5% EtOAc : n-hexanes) to yield 3β-(*tert*-butyldimethylsilyloxy)-androst-5-en-17β-yl -propan-2-yl succinate diester (348 mg, 81%) as a white solid.

m.p 105-107 °C; **^{1}H NMR** (400 MHz, $CDCl_3$) δ5.31 (d, *J* = 4.9 Hz, 1H), 5.01 (hept, *J* = 6.3 Hz, 1H), 4.61 (t, *J* = 8.4 Hz, 1H), 3.47 (tt, *J* = 10.7, 4.7 Hz, 1H), 2.65 – 2.54 (m, *J* = 4.4 Hz, 4H), 2.30 – 0.91 (m, 19H), 1.23 (d, *J* = 6.3 Hz, 6H), 1.00 (s, 3H), 0.88 (s, 9H), 0.80 (s, 3H), 0.05 (s, 6H); **^{13}C NMR** (101 MHz, $CDCl_3$) δ; 172.4, 171.9, 141.8, 120.9, 83.2, 72.7, 68.2, 51.2, 50.3, 42.9, 42.6, 37.5, 36.9, 36.8, 32.2, 31.9, 31.6, 29.8, 29.6, 27.6, 26.1, 23.7, 22.0 (2C), 20.7, 19.6, 18.4, 12.1, -4.4 (2C); **LRMS** (ESI+) found *m/z* 547.4 $[M+H]^+$; **HRMS** (ESI+) found *m/z* 569.3638 $[M+Na]^+$, theoretical ($C_{32}H_{54}O_5SiNa$) *m/z* 569.3633 $[M+Na]^+$; **IR** 2938, 1729 cm^{-1}; **specific rotation** $[\alpha]_D^{25}$ -44.00 (c 1.0, $CHCl_3$).

3β-Hydroxyandrost-5-en-17β-yl -propan-2-yl succinate diester (11)

Procedure adapted from Yamashita *et al.,* (2005) *(7)*. Camphor sulfonic acid (0.302 g, 1.30 mmol) was added to a stirring solution of 3β-(*tert*-butyldimethylsilyloxy)-androst-5-en-17β-yl succinate-propan-2-yl diester (0.360 g, 0.650 mmol) in CH_2Cl_2 (10 mL) and MeOH (10 mL). The reaction mixture was stirred at room temperature for 25 minutes. The reaction was diluted with CH_2Cl_2 (50 mL) and saturated $NaHCO_3$ solution (50 mL). The aqueous layer was further extracted with CH_2Cl_2 (2 x 50 mL). The combined organic extract was washed with saturated

NaCl solution, dried over anhydrous Na_2SO_4 and filtered. The solvent was removed under reduced pressure and the residue obtained was purified by silica column chromatography (20% EtOAc : n-hexanes) to yield 3β-hydroxyandrost-5-en-17β-yl -propan-2-yl succinate diester (265 mg, 94%) as a white solid.

m.p 105-107 °C; **^{1}H NMR** (400 MHz, $CDCl_3$) δ 5.34 (d, *J* = 5.4 Hz, 1H), 5.01 (hept, *J* = 6.3 Hz, 1H), 4.66 – 4.57 (m, 1H), 3.57 – 3.46 (m, 1H), 2.63 – 2.55 (m, 4H), 2.32 – 0.91 (m, 19H), 1.23 (d, *J* = 6.3 Hz, 6H), 1.01 (s, 3H), 0.80 (s, 3H), OH not observed; **^{13}C NMR** (101 MHz, $CDCl_3$) δ 172.4, 171.9, 141.0, 121.4, 83.2, 71.8, 68.2, 51.2, 50.2, 42.6, 42.4, 37.4, 36.9, 36.7, 31.8, 31.7, 31.6, 29.7, 29.6, 27.6, 23.7, 21.9 (2C), 20.7, 19.6, 12.1; **LRMS** (ESI+) found *m/z* 433.3 $[M+H]^+$; **HRMS** (ESI+) found *m/z* 455.2773 $[M+Na]^+$, theoretical ($C_{26}H_{40}O_5Na$) *m/z* 455.2768 $[M+Na]^+$; **IR** 3443, 2933, 1730 cm^{-1}; **specific rotation** $[\alpha]_D^{25}$ –38.80 (c 1.0, $CHCl_3$).

Scheme E)

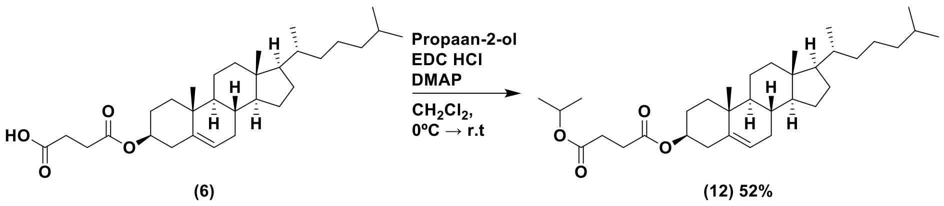

Cholester-3β-yl propan-2-yl succinate diester (12)

A solution of cholesterol hemisuccinate (20.1 mg, 0.041 mmol) and EDC.HCl (15.8 mg, 0.082 mmol) in dry dichloromethane (1 mL) was stirred at 0 °C for 5 minutes under N_2. To the solution, DMAP (9.9 mg, 0.082 mmol), isopropanol (6.3 µL, 0.082 mmol) and additional dichloromethane (1 mL) were added. The reaction mixture was warmed up to ambient temperature and left to stir under N_2 overnight. The solution was then quenched by dropwise addition of 5% citric acid solution (5 mL). The pale-yellow solution was then extracted with ethyl acetate (3 x 5 mL) to give a colourless organic extract. The combined organic extracts were then washed with saturated brine solution (10 mL) and dried over anhydrous $MgSO_4$. The crude material was purified by flash chromatography (silica, 1:3 ethyl acetate:hexanes) to afford Cholester-3β-yl propan-2-yl succinate diester (11.4 mg, 52%) as a white solid.

^{1}H NMR (400 MHz, $CDCl_3$) δ 5.35 (d, *J* 4.05 Hz, 1H), 4.99 (sept., *J* 6.3 Hz, 1H), 4.60 (m, 1H), 2.56 (s, 4H), 2.29 (d, *J* 7.8 Hz, 2H), 2.02 – 1.76 (m, 5H), 1.61 – 1.25 (m, 12H), 1.21 (d, *J* 6.3 Hz, 6H), 1.17 – 1.02 (m, 7H), 0.99 (s, 3H), 0.96 – 0.91 (m, 2H), 0.89 (d, *J* 6.5 Hz, 3H), 0.84 (dd, *J* 6.6, 1.8 Hz, 6H), 0.65 (s, 3H); **^{13}C NMR** (175 MHz, $CDCl_3$) δ 172.1, 171.9, 139.8, 122.9, 74.5, 68.2, 56.9, 56.4, 50.2, 42.5, 39.9, 39.7, 38.3, 37.2, 36.8, 36.4, 36.0, 32.1, 32.1, 29.8, 29.8, 28.4, 28.2, 28.0, 24.5, 24.0, 23.0, 22.8, 22.0, 21.2, 19.5, 18.9, 12.1; **LRMS** (ESI+) found *m/z* 551 $[M+Na]^+$; **HRMS** (ESI+) found *m/z* 529.4266 $[M+H]^+$, theoretical ($C_{34}H_{57}O_4$) *m/z* 529.4251 $[M+H]^+$; **IR** (ATR, solid) 2941, 2890, 2869, 2852, 1728, 1467, 1373, 1318, 1309, 1164, 1107, 996, 982, 958 cm^{-1}; **specific rotation** $[\alpha]_D^{25}$ -24.78 (c 1.0, $CHCl_3$).

Scheme F)

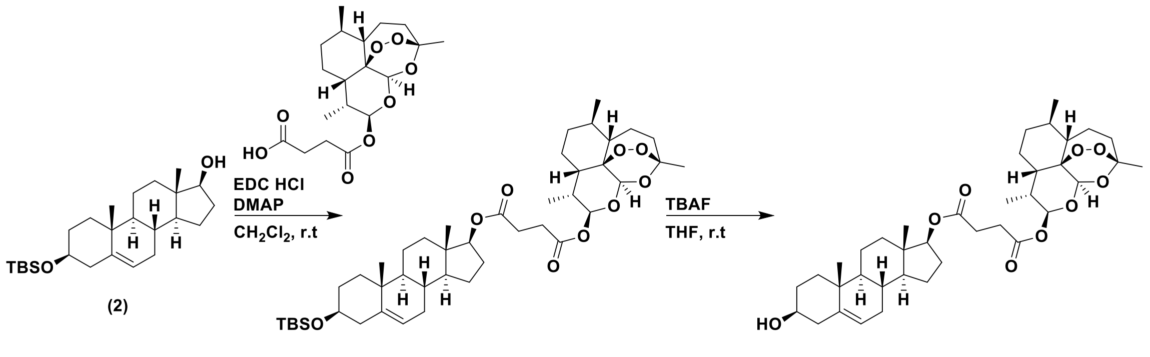

(2) (13) 54% (14) 57%

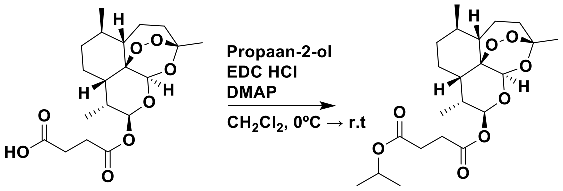

(15) 42%

3β-(*tert*-Butyldimethylsilyloxy)-androst-5-en-17β-yl artesunate ester (13)

A solution of artesunate (10.9 mg, 0.028 mmol) and EDC.HCl (10 mg, 0.052 mmol) was stirred in dichloromethane (2 mL) in a flame dried round bottom flask under N_2. After 5 m a solution of DMAP (6.4 mg, 0.052mmol) and 3β-(*tert*-butyldimethylsilyloxy)-androst-5-en-17β-ol (21.1 mg, 0.052 mmol) in dichloromethane (2 mL) was added. The combined solution was left stirring at room temperature for 20 h before being quenched with 5 % citric acid solution (5 mL) and extracted with EtOAc (4 x 5 mL). The combined organic layers were washed with brine (10 mL), dried over anhydrous $MgSO_4$ and finally concentrated under reduced pressure to give a crude white solid. The crude product was purified by flash chromatography (silica, 10% EtOAc in hexanes) to give 3β-(*tert*-butyldimethylsilyloxy)-androst-5-en-17β-yl artesunate ester (11.9 mg, 54%) as a clear oil.

^{1}H NMR (600 MHz, $CDCl_3$) δ 5.79 (d, *J* = 9.9 Hz, 1H), 5.43 (s, 1H), 5.32 – 5.29 (m, 1H), 4.62 – 4.59 (m, 1H), 3.50 – 3.44 (m, 1H), 2.74 – 2.54 (m, 5H), 2.40 – 0.82 (m, 30H), 1.43 (s, 3H), 1.00 (s, 3H), 0.96 (d, *J* = 6.2 Hz, 3H), 0.88 (s, 9H), 0.85 (d, *J* = 7.1 Hz, 3H), 0.79 (s, 3H), 0.05 (s, 6H); **^{13}C NMR** (151 MHz, $CDCl_3$) δ 172.2, 171.3, 141.8, 120.9, 104.6, 92.2, 91.6, 83.3, 80.3, 72.7, 51.7, 51.2, 50.3, 45.4, 42.9, 42.6, 37.5, 37.4, 36.9, 36.8, 36.4, 34.2, 32.2, 31.9, 31.9, 31.6, 29.5, 29.3, 27.6, 26.1, 26.1, 24.7, 23.7, 22.1, 20.7, 20.4, 19.6, 18.4, 12.3, 12.2, -4.4(2C); **LRMS** (ESI+) found *m/z* 793 $[M+Na]^+$; **HRMS** (ESI+) found *m/z* 793.4686 $[M+Na]^+$, theoretical ($C_{44}H_{70}O_9SiNa$) *m/z* 793.4681 $[M+Na]^+$; **specific rotation** $[\alpha]_D^{25}$ -14.36 (c 0.8, $CHCl_3$).

3β-Hydroxyandrost-5-en-17β-yl artesunate ester (14)

A solution of 3β-(*tert*-butyldimethylsilyloxy)-androst-5-en-17β-yl succinate-artesunate ester (30 mg, 0.039 mmol) in dry tetrahydrofuran (10mL) was treated with 1M TBAF solution in THF (140 μL, 0.14 mmol) and stirred for 40 h at room temperature. The solvent was removed under reduced pressure to give a crude yellow oil. The crude material was purified by flash chromatography (silica, 1:1 ethyl acetate:hexanes) to afford 3β-hydroxyandrost-5-en-17β-yl artesunate ester (14.8 mg, 57 %) as a colourless oil.

^{1}H NMR (400 MHz, $CDCl_3$) δ 5.79 (d, *J* 9.8 Hz, 1H), 5.43 (s, 1H), 5.34 (d, *J* 5.0 Hz, 1H), 4.61 (t, *J* 8.4 Hz, 1H) 3.52 (td, *J* 5.4, 11.2 Hz, 1H), 2.72-0.88 (m, 36H) 1.43, (s, 3H), 1.01 (s, 3H), 0.96 (d, *J* 6.2 Hz, 3H), 0.85 (d, *J* 7.2 Hz, 3H), 0.80 (s, 3H); **^{13}C NMR** (175 MHz, $CDCl_3$) δ 172.2, 171.3, 141.0, 121.4, 104.6, 92.3, 91.6, 83.3, 80.3, 71.9, 60.5, 51.7, 51.2, 50.2, 45.4, 42.6, 42.4, 37.4, 36.9, 36.7, 36.4, 34.3, 31.9, 31.9, 31.8, 31.6, 29.5, 29.3, 27.6, 26.1, 24.7, 22.2, 20.7,

20.4, 19.6, 14.3, 12.2, 12.2; **LRMS** (ESI+) found *m/z* 679 [M+Na]$^+$; **HRMS** (ESI+) found *m/z* 679.3815 [M+Na]$^+$, theoretical ($C_{38}H_{56}O_9Na$) *m/z* 679.3817 [M+Na]$^+$; **IR** (ATR, solid) 3450, 2930, 1735, 1454, 1375, 1158, 1037, 1015, 877 cm^{-1}.

Propan-2-yl artesunate ester (15)

A solution of artesunate (20 mg, 0.052 mmol) and EDC.HCl (19.9 mg, 0.104 mmol) was stirred in dry dichloromethane (1mL) at 0 °C for 5 minutes under N_2. To the solution, DMAP (12.7 mg, 0.104 mmol), isopropanol (7.78 µL, 0.104 mmol) and additional dichloromethane (1 mL) were added. The reaction mixture was warmed up to ambient temperature and left to stir under N_2 for 4 h. Additional isopropanol (7.78 µL, 0.054 mmol) was added and the reaction was stirred for another 2 h. The solution was then quenched by dropwise addition of 5% citric acid solution (5 mL). The colourless solution was then extracted with ethyl acetate (3 x 5 mL) to give a colourless organic extract. The extract was then washed with saturated brine solution (10 mL) and dried over anhydrous $MgSO_4$. The crude material was purified by flash chromatography (silica, 1:3 ethyl acetate:hexanes) to afford propan-2-yl artesunate ester (9.4 mg, 42 %) as a white solid.

^{1}H NMR (400 MHz, $CDCl_3$) δ 5.77 (d, *J* 9.9 Hz, 1H), 5.41 (s, 1H), 4.99 (sept., *J* 6.2 Hz, 1H), 2.71 – 2.50 (m, 5H), 2.35 (td, *J* 14.0, 4.0 Hz, 1H), 2.00 (ddd, *J* = 14.6, 4.7, 3.1 Hz, 1H), 1.81 (m, 1H), 1.72 (m, 2H), 1.62 – 1.57 (m, 1H), 1.51 – 1.43 (m, 1H), 1.40 (s, 3H), 1.37 – 1.23 (m, 3H), 1.20 (dd, *J* 6.3, 0.8 Hz, 6H), 1.06 – 0.96 (m, 1H), 0.94 (d, *J* 5.9 Hz, 3H), 0.83 (d, *J* 7.1 Hz, 3H); **^{13}C NMR** (175 MHz, $CDCl_3$) δ 171.8, 171.4, 104.7, 92.3, 91.7, 80.3, 68.3, 51.8, 45.5, 37.5, 36.5, 34.3, 32.0, 29.5, 29.5, 26.2, 24.8, 22.2, 22.0 (2C), 20.4, 12.3; **LRMS** (ESI+) found

m/z 449 [M+Na]$^+$; **HRMS** (ESI+) found *m/z* 449.2149 [M+Na]$^+$, theoretical ($C_{22}H_{34}O_8Na$) *m/z* 449.2146 [M+Na]$^+$; **IR** (ATR, solid) 2976, 2928, 2875, 1750, 1730, 1454, 1375, 1201, 1159, 1102, 1036, 1012, 926, 876, 825 cm^{-1}; **specific rotation** $[\alpha]_D^{25}$ + 11.18 (c 1.0, $CHCl_3$).

Scheme G)

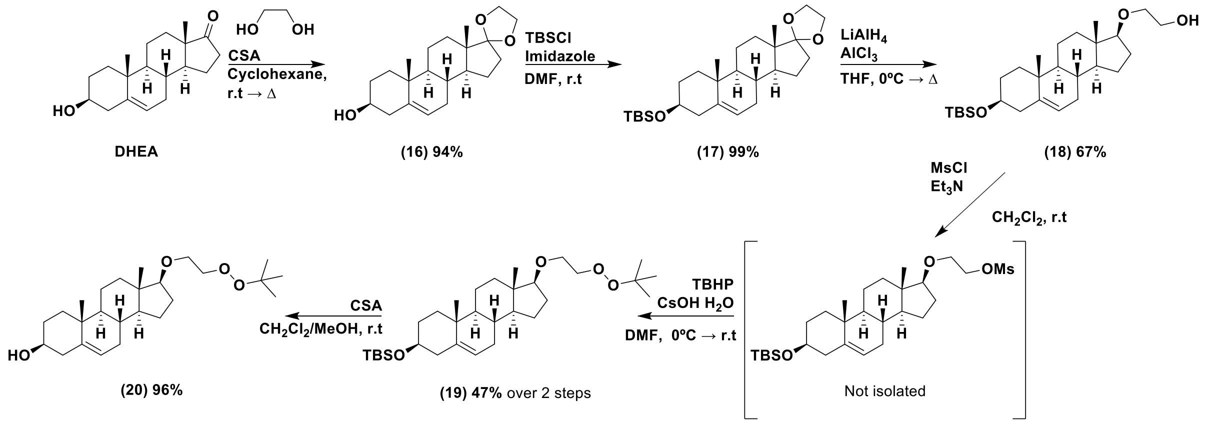

17,17-(Ethylenedioxy)-androst-5-en-3β-ol (16)

Procedure adapted from Calogeropoulou et al., (2009) *(11)*. 3β-Hydroxyandrost-5-en-17-one (1.0 g, 3.5 mmol) was combined with ethylene glycol (0.60 mL, 10 mmol) and camphor sulfonic acid (10 mg, 0.14 mmol) in cyclohexane (100 mL). The reaction flask was fitted with a Dean-Stark apparatus, stirred, and brought to reflux for 4 hours. The reaction was cooled, diluted with EtOAc (50 mL) and poured into saturated $NaHCO_3$ solution (50 mL). The aqueous layer was further extracted with EtOAc (2 x 50 mL). The combined organic extract was washed with saturated NaCl solution, dried over anhydrous Na_2SO_4 and filtered. The solvent was

removed under reduced pressure to yield 17,17-(ethylenedioxy)-androst-5-en-3β-ol (1.08 g, 94%) as a white solid.

m.p 162-165 °C (lit. *(11)* 162-165 °C); **^{1}H NMR** (400 MHz, $CDCl_3$) δ 5.37 – 5.31 (m, 1H), 3.95 – 3.83 (m, 4H), 3.52 (m, 1H), 2.31 – 0.93 (m, 19H), 1.00 (s, 3H), 0.85 (s, 3H), OH not observed; **^{13}C NMR** (101 MHz, DMSO-*d_6*) δ 141.3, 120.2, 118.5, 69.9, 64.6, 64.0, 50.2, 49.7, 45.1, 42.2, 36.9, 36.1, 33.7, 31.7, 31.4, 30.8, 30.2, 22.3, 20.1, 19.2, 14.1; **LRMS** (ESI+) found *m/z* 355.2 $[M+Na]^+$; **HRMS** (ESI+) found *m/z* 355.2259 $[M+Na]^+$, theoretical ($C_{21}H_{32}O_3Na$) *m/z* 355.2244 $[M+Na]^+$; **IR** 3546, 3140, 2989, 2828 cm^{-1}

3β-(*tert*-Butyldimethylsilyloxy)-17,17-(ethylenedioxy)-androst-5-ene (17)

Procedure adapted from Yamauchi *et al.,* (2005) *(2)*. *tert*-Butyldimethylsilyl chloride (0.580 g, 3.84 mmol) was added to a stirring solution of 17,17-(ethylenedioxy)-androst-5-en-3β-ol (0.850 g, 2.56 mmol) and imidazole (0.436 g, 6.40 mmol) in anhydrous DMF (10 mL). The reaction mixture was stirred at room temperature for 2 hours. The reaction was diluted with water (50 mL) and extracted with EtOAc (50 mL). The aqueous layer was further extracted with EtOAc (2 x 50 mL). The combined organic extract was washed with saturated NaCl solution, dried over anhydrous $MgSO_4$ and filtered. The solvent was removed under reduced pressure to yield 3β-(*tert*-butyldimethylsilyloxy)-17,17-(ethylenedixoy)-androst-5-ene (1.125 g, 99%) as a white solid.

m.p 119-121 °C (lit. *(12)* 121-122 °C); **^{1}H NMR** (400 MHz, $CDCl_3$) δ 5.34 – 5.28 (m, 1H), 3.96 – 3.84 (m, 4H), 3.48 (m, 1H), 2.29 – 0.92 (m, 19H), 1.00 (s, 3H), 0.88 (s, 9H), 0.85 (s, 3H), 0.05 (s, 6H); **^{13}C NMR** (101 MHz, dioxane-*d_8*) δ 142.23, 121.66, 119.94, 73.29, 65.67, 65.07, 51.47, 51.13, 46.46, 43.60, 38.07, 37.40, 34.81, 33.02, 32.88, 32.08, 31.43, 26.30, 23.46, 21.26, 19.70, 18.74, 14.62, -4.36 (2C); **LRMS** (ESI+) found *m/z* 469.3 $[M+Na]^+$, **HRMS** (ESI+) found *m/z* 469.3118 $[M+Na]^+$, theoretical ($C_{27}H_{46}O_3SiNa$) *m/z* 469.3108 $[M+Na]^+$; **IR** 2987, 2886 cm^{-1}.

3β-(*tert*-Butyldimethylsilyloxy)-17β-(2-hydroxyethoxy)-androst-5-ene (18)

Procedure adapted from Upasani *et al.,* (1999) *(13)*. 3β-(*tert*-Butyldimethylsilyloxy)-17,17-(ethylenedioxy)-androst-5-ene (1.00 g, 2.24 mmol) in anhydrous THF (12 mL) was cooled over an ice bath under N_2 atmosphere. $AlCl_3$ (0.600 g, 4.48 mmol) in anhydrous THF (5 mL) and $LiAlH_4$ (0.170 g, 4.48 mmol) in anhydrous THF (5 mL) were added individually by dropwise addition to the previous solution with stirring. The reaction was brought to reflux and stirred for 2 hours. The reaction was then cooled over an ice bath and quenched by addition of 10% Rochelle's salt solution (10 mL) and EtOAc (15 mL). The mixture was left stirring for a further 20 minutes. The solution was then diluted with EtOAc (50 mL) and 10% Rochelle's salt (50 mL). The aqueous layer was further extracted with EtOAc (2 x 50 mL). The combined organic extract was washed with saturated NaCl solution, dried over anhydrous $MgSO_4$ and filtered. The solvent was removed under reduced pressure and the residue obtained was purified by silica column chromatography (15% EtOAc : n-hexanes) to yield 3β-(*tert*-butyldimethylsilyloxy)-17β-(2-hydroxyethoxy)-androst-5-ene (0.676 g, 67%) as a white solid.

m.p 117-119 °C; **^{1}H NMR** (400 MHz, $CDCl_3$) δ 5.30 (m, 1H), 3.71 – 3.66 (m, 2H), 3.61 – 3.44 (m, 3H), 3.35 (t, *J* = 8.3 Hz, 1H), 2.29 – 0.91 (m, 19H), 1.00 (s, 3H), 0.88 (s, 9H), 0.77 (s, 3H), 0.05 (s, 6H), OH not observed; **^{13}C NMR** (101 MHz, $CDCl_3$) δ 141.8, 121.0, 89.6, 72.7, 71.1, 62.2, 51.8, 50.4, 43.0, 42.9, 38.0, 37.5, 36.8, 32.2, 31.9, 31.7, 28.2, 26.1, 23.5, 20.9, 19.6, 18.4, 11.7, -4.4 (2C); **LRMS** (ESI+) found *m/z* 471.3 $[M+Na]^+$; **HRMS** (ESI+) found *m/z* 471.3284 $[M+Na]^+$, theoretical ($C_{27}H_{48}O_3SiNa$) *m/z* 471.3265 $[M+Na]^+$; **IR** 3462, 2927, 2856 $cm^{-}1$; **specific rotation** $[\alpha]_D^{25}$ -33.20 (c 1.0, $CHCl_3$).

17β-(2-(*tert*-Butylperoxy)-ethoxy)-3β-(*tert*-butyldimethylsilyloxy)-androst-5-ene (19)

Procedure adapted from Dussault *et al.,* (2000) *(14)*. Methane sulfonyl chloride (180 mL, 2.27 mmol) was added to a cooled and stirring solution of 3β-(*tert*-butyldimethylsilyloxy)-17β-(2-hydroxyethoxy)-androst-5-ene (300 mg, 0.670 mmol) and Et_3N (340 mL, 2.44 mol) in CH_2Cl_2. The reaction was brought to room temperature and stirred under N_2 for 45 minutes. The solvent was removed under reduced pressure and yellow residue obtained was redissolved in anhydrous DMF (30 mL) and added to a cooled solution of CsOH monohydrate (500 mg, 3.35 mmol) and *tert*-butyl hydroperoxide (~ 240 mL, 1.2 mmol) (nominally 5.0 M in decane) in anhydrous DMF (12 mL). The reaction was brought to room temperature and stirred under N_2 for 24 hours. A further amount of CsOH monohydrate (430 mg, 2.86 mmol) was added producing a yellowish solution which was stirred at room temperature for a further 24 hours. The reaction mixture was diluted with EtOAc (50 mL) and water (50 mL). The aqueous layer was further extracted with EtOAc (2 x 50 mL). The combined organic extract was washed saturated NaCl solution, dried over anhydrous Na_2SO_4 and filtered. The solvent was removed under reduced pressure and the residue obtained was purified by silica column chromatography

(100% hexane, followed by 10% EtOAc : n-hexanes) to yield 17β-(2-(*tert*-butylperoxy)-ethoxy)-3β-(*tert*-butyldimethylsilyloxy)-androst-5-ene (198 mg, 57%) as a white solid.

m.p 122-124 °C; **^{1}H NMR** (400 MHz, $CDCl_3$) δ 5.30 (d, J = 4.6 Hz, 1H), 4.05 (t, J = 5.1 Hz, 2H), 3.74 – 3.57 (m, 2H), 3.47 (tt, J = 10.5, 4.6 Hz, 1H), 3.33 (t, J = 8.2 Hz, 1H), 2.33 – 2.21 (m, 1H), 2.21 – 2.11 (m, 1H), 2.05 – 1.89 (m, 3H), 1.86 – 1.77 (m, 1H), 1.74 – 1.68 (m, 1H), 1.63 – 0.84 (m, 12H), 1.24 (s, 9H), 1.00 (s, 3H), 0.88 (s, 9H), 0.77 (s, 3H), 0.05 (s, 6H); **^{13}C NMR** (101 MHz, $CDCl_3$) δ 141.8, 121.0, 89.6, 80.4, 74.8, 72.7, 67.5, 51.8, 50.5, 43.0 (2C), 38.0, 37.6, 36.8, 32.2, 31.9, 31.7, 28.1, 26.5, 26.1, 23.5, 20.9, 19.6, 18.4, 11.6, -4.4 (2C); **LRMS** (ESI+) found *m/z* 543.4 $[M+Na]^+$; **HRMS** (ESI+) found *m/z* 543.3848 $[M+Na]^+$, theoretical ($C_{31}H_{56}O_4SiNa$) *m/z* 543.3840 $[M+Na]^+$; **specific rotation** $[\alpha]_D^{25}$ +256.60 (c 1.0, $CHCl_3$).

17β-(2-(*tert*-Butylperoxy)-ethoxy)-androst-5-en-3β-ol (20)

Procedure adapted from Yamashita *et al.,* (2005) *(7)*. Camphor sulfonic acid (106 mg, 0.456 mmol) was added to a stirring solution of 17β-(2-(*tert*-butylperoxy)-ethoxy)-3β-(*tert*-butyldimethylsilyloxy)-androst-5-ene (120 mg, 0.228 mmol) in CH_2Cl_2 (5 mL) and MeOH (5 mL). The reaction was stirred at room temperature for 1 hour. The reaction mixture was diluted with CH_2Cl_2 (50 mL) and saturated $NaHCO_3$ solution (50 mL). The aqueous layer was further extracted with CH_2Cl_2 (2 x 40 mL). The combined organic extract was washed with saturated NaCl solution, dried over anhydrous Na_2SO_4 and filtered. The solvent was removed under reduced pressure and the residue obtained was purified by silica column chromatography (20% EtOAc : n-hexanes) to yield 17β-(2-(*tert*-butylperoxy)-ethoxy)-androst-5-en-3β-ol (89 mg, 96%) as a white oily solid.

^{1}H NMR (400 MHz, $CDCl_3$) δ 5.34 (d, *J* = 5.0 Hz, 1H), 4.05 (t, *J* = 5.1 Hz, 2H), 3.73 – 3.58 (m, 2H), 3.52 (tt, *J* = 10.9, 4.7 Hz, 1H), 3.34 (t, *J* = 8.2 Hz, 1H), 2.35 – 2.18 (m, 2H), 2.07 – 1.90 (m, 3H), 1.88 – 1.79 (m, 2H), 1.62 – 0.88 (m, 12H), 1.25 (s, 9H), 1.01 (s, 3H), 0.78 (s, 3H), OH not observed; **^{13}C NMR** (101 MHz, $CDCl_3$) δ 141.0, 121.6, 89.6, 80.4, 74.8, 71.9, 67.5, 51.7, 50.4, 43.0, 42.4, 38.0, 37.4, 36.7, 31.9, 31.8, 31.6, 28.1, 26.5, 23.5, 20.9, 19.6, 11.6; **LRMS** (ESI+) found *m/z* 429.3 $[M+Na]^+$; **HRMS** (ESI+) found *m/z* 429.2969 $[M+Na]^+$, theoretical ($C_{25}H_{42}O_4Na$) *m/z* 429.2975 $[M+Na]^+$; **IR** 3362 cm^{-1}; **specific rotation** $[\alpha]_D^{25}$ -49.50 (c 1.0, $CHCl_3$).

Biological Methods

Uptake of Fluorescent Cholesterol Analogues:

Cholesterol depleted media was prepared by removing lipoproteins from serum by ultracentrifugation according to methods by Havel *et al. (15)* with modifications as described below. The density of heat-inactivated human serum was adjusted to 1.21 g/mL by addition of KBr. The solution was centrifuged at 220,000 *g* for 48 hours at 10°C using a Beckman SW 41 Ti rotor. The top layers were carefully removed, and the bottom layer was then diluted with MilliQ water to wash and centrifuged for an additional 24 hours under the same conditions as above. The top layers were carefully removed as above. The bottom fraction was taken as cholesterol-depleted serum and transferred to 3.5 molecular weight cut off dialysis tubing (Thermofisher 88242). This was exhaustively dialysed in four changes of PBS (2 L, pH 7.4) over 24 hours at 4°C. The final product was sterilised by passing through a 0.2 µm filter and stored at -20°C until use. Culture medium for this experiment was prepared from RPMI 1640-HEPES with Glutamax supplemented with 10 mM D-Glucose, 480 µM hypoxanthine, 20 µg/mL gentamicin and 10% v/v cholesterol-depleted serum as described above (cholesterol-depleted media). Cholesterol concentration was assessed using an Amplex™ Red Cholesterol Assay Kit (Thermofisher A12216) according to the manufacturer's instructions, indicating >98% depletion of cholesterol.

Fluorescent cholesterol analogues used in this study are dehydroergosterol (Cayman Chemical 19709), 24-BODIPY-Cholestrol (Cayman Chemical 24618), 3-Undeconoate-BODIPY-Cholesterol (Thermo Fisher C12680), 22-NBD-Cholesterol (Thermo Fisher N1148), 3-Hexanoyl-NBD-cholesterol (Cayman Chemical 13221). NBD without cholesterol is *p*-methoxybenzylamie-NBD (Santa Cruz Biotechnology sc-214208). Ring-stage parasites at 4% parasitemia and 2% haematocrit were incubated with 2 µM cholesterol analogues or solvent

controls in cholesterol-depleted media for 24 hours at 37°C under microaerophilic conditions. Cells were washed twice in PBS with 10 mM D-Glucose (PBS-G), and then resuspended in 5 µg/mL Hoechst 33342 (Thermo Fisher 14533) in PBS-G and incubated for 20 minutes at 37°C. Cells stained with dehydroergosterol were instead incubated with 500 nM Mitotracker™ Deep Red FM (Thermo Fisher M22426) for 15 minutes and then washed twice in PBS-G. Samples were imaged with fluorescence microscopy or read on a flow cytometer.

Images were collected and deconvoluted on a Deltavision Deconvolution microscope at 1,000× magnification, with a resolution of 67 nm per pixel. NBD and BODIPY fluorescence were detected at 475/28 nm excitation (ex) and 525/48 nm emission (em). Hoechst fluorescence (nucleic acid) and dehydroergosterol were detected at 390/18 nm ex and 435/48 nm em. For each fluorescent cholesterol analogue, images were collected under the same exposure conditions (without binning) and converted to TIFF files under the same brightness and contrast settings. Individual cells were cropped from larger images with Fiji ImageJ *(16)*. No other manipulations were performed.

For quantification, events were measured on a LSR II or LSRFortessa Flow Cytometer. NBD fluorescence was detected at 488 nm ex and 530/30 nm em. Hoechst 33342 (Thermo Fisher) fluorescence was detected at 410 nm ex and 450/50 nm em. Data were initially processed using FlowJo. RBCs were gated on FSC and SCC and doublets excluded. iRBCs (positive) and uRBCs (negative) were differentiated by Hoechst fluorescence. The geometric mean of fluorescence intensity (mean fluorescence intensity, MFI) was calculated with FlowJo. Background fluorescence was subtracted based on unstained controls (buffer or solvent only). MFI data was normalised between experiments by setting the fluorescence of uRBCs to 1.

Growth and Viability Inhibition Assays:

***Plasmodium falciparum* Growth Inhibition Assays:**

P. falciparum parasites were maintained under routine culture conditions in red blood cells and RPMI 1640-Hepes with Glutamax, supplemented with 10 mM D-glucose, 480 µM hypoxanthine, 20 µg/mL gentamicin, 0.375% (w/v) AlbuMAX^{TM} II (Thermo Fisher 11021029), and 2.5% v/v heat-inactivated human serum *(17)*. Cultures were maintained at 37°C under microaerophilic conditions (94% N_2, 5% CO_2, 1% O_2). All experiments used 3D7 wildtype parasites except for the ring stage survival assay, as specified. Cultures were synchronised with 5% w/v D-sorbitol *(18)*.

Growth inhibition was investigated according to methods described by Smilkstein *et al. (19)* with modifications by Spry *et al. (20)*. Compounds or solvent controls (DMSO, ethanol, or ethyl acetate) were serially diluted 2-fold across flat-bottom 96-well plates. Culture media without compound was used as a negative control. 200 nM chloroquine (Sigma C6628) or artesunate (Sigma A3731) was used as a positive control to determine background fluorescence. The parasitaemia of a culture containing predominantly ring stage parasites was counted by Giemsa smear. The parasitaemia was adjusted to 1% by addition of fresh uRBCs and added to the plate at a final haematocrit of 1%, with 200 µL per well. The plates were incubated at 37°C under humid microaerophilic conditions. After 24, 48, or 72 hours, the plates were transferred to a -18°C freezer to stop the assay and lyse cells. Plates were thawed and lysate was mixed 1:1 with SYBR Safe DNA Gel Stain (Invitrogen 33102) (final concentration 1/10,000) diluted in lysis buffer (20 mM TRIS, 5 mM EDTA, 0.008% w/v Saponin, 0.08% w/v Triton-X 100, pH 7.5). Fluorescence was detected at 490 nm excitation/ 520 nm emission on a FLUOstar Optima fluorescence plate reader (BMG Labtech).

***Plasmodium falciparum* Gametocyte Viability Assays:**

Asexual *P. falciparum* parasites were induced to form gametocytes as described *(21)*. Synchronized trophozoite-stage parasites at 2% parasitaemia were incubated overnight and "stressed" by replacing only 25% of the culture medium, and 67% the following day, with the haematocrit maintained at 4%. On the subsequent day (designated as day 0) of gametocytogenesis, the culture containing early ring-stage parasites was first purified by sorbitol synchronisation to remove asynchronous trophozoites, and passed through a magnetic field in a sterile MACS® column in a SuperMACS™ II magnet to remove remaining late-stage parasites or later-stage gametocytes (negative selection). The culture medium was supplemented with 50 mM N-acetyl-D-glucosamine to inhibit asexual parasite proliferation, and changed daily. Sorbitol treatment was performed on Day 1, 5, and 6 to further remove asexual parasites. Gametocytes were magnetically enriched (positive selection) in a sterile column with a SuperMACS™ II magnet and eluted in culture media with 50 mM N-acetyl-D-glucosamine. These gametocytes were incubated overnight at 37°C under microaerophilic conditions, and purity was assessed by examination under a light microscope (400× magnification).

Gametocyte viability was assessed as described by Ridgway *et al. (21)* with modifications. Compounds or solvent controls (DMSO) were serially diluted across 96-well plates in culture media supplemented with 50 mM N-acetyl-D-glucosamine. Culture media without compound was used as a negative control. 100 mM artemisinin (Sigma 361593) was used as a positive control to determine background gametocyte fluorescence. Stage III-IV gametocytes (Day 7) or stage V (day 10) gametocytes were concentrated and added to the plate with 200 µL per well. The plate was incubated for 48 hours at 37°C under humid microaerophilic conditions. Parasites were then stained with 500 nM MitoTracker™ Deep Red FM and 5 µg/ml Hoechst

33342 in PBS-G for 30 minutes and washed twice in PBS-G. Samples were read on a flow cytometer as described above.

Gametocytes were gated with FlowJo on FSC, SCC, and Hoechst fluorescence, and doublets excluded. Live (positive) and dead (negative) gametocytes were differentiated by MitoTracker™ Deep Red FM fluorescence (detected at 633 nm ex and 780/60 nm em), and the percentage of each was calculated.

***Plasmodium berghei* Liver Stage Growth Inhibition Assays:**

Plasmodium berghei ANKA Bergreen *(22)* or GFP-Luc (RMgm-29) *(23)*, respectively expressing green fluorescent protein (GFP) or a GFP-Luciferase fusion protein, were maintained under routine conditions in SWR/J *Mus musculus* mice and *Anopheles stephensi* mosquitos.

Growth inhibition of *P. berghei* liver-stages was investigated after infection of human hepatoma cells (Huh7) and subsequent growth for 48 hours at 37° C, 5% CO_2 and 80% humidity. The day prior to infection, 10,000 Huh7 cells were seeded in dark-sided 96-well microplates (Greiner Bio One). On the day of infection, compounds or solvent controls (DMSO) were serially diluted in culture media and 5,000 – 10,000 Bergreen or GFP-Luc sporozoites were added per well following dissection from infected *A. stephensi* salivary glands. Uninfected wells served as control for background luminescence. To promote invasion, the plates were centrifuged at 1,550 *g* for 5 minutes. After 60 minutes, the media was completely replaced with fresh, prewarmed media with the matching drug concentrations. Media was partially replaced with new drug dilutions over the following two days. 48 hours post-infection, luminescence signal was measured by addition of 1:1 ONE-Glo™ substrate (Promega) to the wells, followed by detection at 300 – 700 nm using a Synergy HTX plate reader (Agilent).

For measurement of parasite size and number, 20,000 Huh7 cells were seeded onto collagen-coated coverslips on the day prior to infection. As above, 10,000 – 20,000 sporozoites were added per slide, allowed to invade for 60 minutes after centrifugation, and then incubated for 48 hours at 37°C, 5% CO_2, and 80% humidity. Infected cells were fixed with 3% paraformaldehyde in PBS, permeabilized with 0.3% v/v Triton X-100, and labelled with 1:1,000 polyclonal chicken anti-GFP (Abcam ab13970), 1:2,000 goat anti-chicken Alexa Fluor™ 546 (Invitrogen A-11040) or Alexa Fluor™ 488 (Invitrogen A-11039) to ensure that intrinsic GFP fluorescence could be detected, as well as Fluoromount-G® containing 4′,6-diamidino-2-phenylindole (DAPI; Southern Biotech), to stain parasite and host cell nuclei. Images were collected on a Zeiss Axio Observer Z2 microscope with a Zeiss AxioCam MRm at 200× or 400× (for quantification) or 630× (for cell images) magnification, with a resolution of 102 nm, 163 nm or 102 nm per pixel respectively. Alexa Fluor™ 546 fluorescence was detected at 546/13 nm excitation and 675/35 nm emission. GFP/Alexa Fluor™ 488 fluorescence was detected at 470/20 nm excitation and 525/25 nm emission. DAPI fluorescence (nucleic acid) was detected at 359/24 nm ex and 445/25 nm em. For quantification, all cells on each coverslip were imaged under the same settings for each experiment. *P. berghei* liver stage size was measured as described *(24)* using Fiji ImageJ *(25)*. Liver-stage parasites were identified and delineated in fluorescent images using defined threshold levels, and the identified fluorescent areas were used to measure parasite area. The number of parasites on each coverslip was also counted. Data are presented as a percentage of the size or parasite number of the solvent control to normalize for differences between experiments. For cell images presented in the figure, images were collected under optimal exposure settings to ensure features were visible. Individual cells were cropped from larger images with Fiji ImageJ, and the brightness was adjusted while ensuring that no fluorescence was removed. No other manipulations were performed.

Toxoplasma Growth Inhibition Assays:

Growth inhibition was investigated as described previously *(26)*. Fluorescent *Toxoplasma gondii* parasites were inoculated into an optical bottom 96-well plate containing confluent human foreskin fibroblast (HFF) host cells at a density of 2,000 parasites/well, with a serial dilution of each compound, or culture media/DMSO (0.1% v/v) alone. Plates were measured daily with a FluoStar Optima fluorescence plate reader at 540 nm excitation and 590 nm emission for one week, and the background fluorescence from the time 0 reading was subtracted from all other measurements. The 72-hour timepoint was chosen for further analysis because parasite growth was in the mid-logarithmic stage (**Figure S2**).

Human Hepatoma Viability Assays:

The Huh7 hepatoma cell line (ATCC) was maintained under routine culture conditions in Dulbecco's Modified Eagle Medium (DMEM) containing 3.7 g/L $NaHCO_3$ supplemented with 10% foetal bovine serum (FBS), 100 units/mL penicillin, and 100 µg/mL streptomycin at 37°C in a humidified 5% CO_2 incubator.

Viability assays were performed in parallel to the *P. berghei* growth inhibition. 10,000 Huh7 cells were seeded in 96-well and dark-sided microplates (Greiner Bio One) and cultured at 37°C, 5% CO_2 and 80% humidity. The next day, the media was replaced with compounds or solvent controls (DMSO) serially diluted in culture media. Media was partially replaced with new drug dilutions over the following two days. After 48 hours, CellTiter-Blue® (Promega) was added according to the manufacturer's instructions and incubated for 3 hours. Fluorescence intensity was measured with 530/25 ex and 590/20 em in a Synergy HTX plate reader (Agilent).

Human Foreskin Fibroblast Growth Inhibition Assays:

The HFF cell line was maintained under routine culture conditions in DMEM containing 3.7 g/L $NaHCO_3$ supplemented with 10% v/v newborn calf serum (NCS), 50 units/mL penicillin, 50 µg/mL streptomycin, 10 µg/mL gentamicin, 0.25 µg/mL amphotericin B, and 0.2 mM L-glutamine at 37°C in a humidified 5% CO_2 incubator. RH *Toxoplasma gondii* parasites expressing the fluorescent protein tdTomato *(26)* were cultured under routine culture conditions in HFF cells and DMEM containing 2 g/L $NaHCO_3$ supplemented with 1% (v/v) FBS, 50 units/mL penicillin, 50 µg/mL streptomycin, 10 µg/mL gentamicin, 0.25 µg/mL amphotericin B, and 0.2 mM L-glutamine at 37°C in a humidified 5% CO_2 incubator.

HFF cells were detached from a flask using 0.25% Trypsin with 0.2 g/L EDTA (Sigma T4049) and washed with culture media. Cells were incubated with a serial dilution of each compound, or media/DMSO alone. Cycloheximide at 10 µg/mL was used as a positive control to determine background fluorescence. Approximately 5 000 cells were seeded in each well. Plates were incubated at 37°C for 72 hours (until reaching confluency) and the media was removed. Plates were transferred to a -18°C freezer. Plates were thawed and SYBR Safe DNA Gel Stain (final concentration 1/10,000) diluted in lysis buffer (as above) was added. Fluorescence was read at 490 nm excitation/ 520 nm emission on a FLUOstar Optima fluorescence plate reader.

Human Embryonic Kidney Cell Viability Assays:

Human Embryonic Kidney Cell (HEK293) culture:

The HEK293 cell line was maintained under routine culture conditions in DMEM containing 3.7 g/L $NaHCO_3$ supplemented with 10% FBS, 200 µM glutamine, 100 units/mL penicillin, and 100 µg/mL streptomycin at 37°C in a humidified 5% CO_2 incubator.

Cells were detached from a flask using 0.25% Trypsin with 0.2 g/L EDTA (Sigma T4049) and washed with culture media containing no phenol red. Cells were incubated with a serial dilution of each compound, or media/DMSO alone. The protein synthesis inhibitor cycloheximide (Sigma 01810) at 10 µg/mL was used as a positive control to determine background absorbance. 5,000 cells were seeded in each well. After 96 hours, 0.45 mg/mL methylthiazolyldiphenyl-tetrazolium bromide (MTT; Sigma M2128) was added and incubated for a further two hours, before mixing 1.1:1 with 10% w/v sodium dodecyl sulfate (SDS) to solubilise crystals and freezing the plate at -18°C to stop the assay. Plates were defrosted at room temperature and absorbance was read at 570 nm in an Infinite M1000 pro plate reader (Tecan).

***Plasmodium falciparum* Ring Stage Survival Assay**

Ring stage survival assays were conducted according to standard protocols with some modifications *(27, 28)*. CAM3.11 and CAM3.11REV parasites were sorbitol synchronised twice, 12 hours apart, and then incubated for a further 30 hours until the majority of the parasites were mature schizonts (with clearly separated nuclei). Pelleted RBCs were incubated at 37°C with heparinised RPMI for 15 minutes, and then centrifuged over 75% Percoll® in PBS for 15 minutes at 1,000 *g*. Mature schizonts were collected, washed with warm RPMI, and cultured for exactly three hours with fresh uRBCs and culture media. After 3 hours, the cultures were sorbitol synchronised and incubated in duplicate with 700 nM of artesunate, PAYb076a, GGA5, HJB8a53, a DMSO solvent-only control or media alone for exactly six hours, before removing the drug, washing in RPMI, and returning to culture conditions. Samples of each cell suspension were transferred to a -18°C freezer at the start of the assay (time = 0) or treated for the entire 72 hours with 700 nM artesunate to determine background fluorescence under no-growth conditions. After a further 66 hours of incubation (totalling 72 hours), cells were

resuspended, and a sample from each well was transferred to a fresh plate and frozen at -18ºC. Microscope slide smears were made using the remaining cells and stained with 10% v/v Giemsa. Plates were thawed and SYBR Safe DNA Gel Stain (final concentration 1/10,000) diluted in lysis buffer (as above) was added for DNA detection at 490 nm excitation / 520 nm emission on a FLUOstar Optima fluorescence plate reader. Parasite proliferation and viability were also assessed using Giemsa-stained smears examined under a light microscope (1,000× magnification); with 'viable' and 'non-viable' (vacuolated or pyknotic) parasites distinguished according to standard protocol. Images were captured using a Leica ICC50 camera, with a resolution of 76 nm per pixel.

Growth Inhibition Data:

Growth inhibition data were normalised by subtracting background fluorescence, luminescence, or percentage of cells from a no-growth or no-viability control (high drug concentration or time = 0 measurement as specified) and expressed as a percentage of the growth or cell percentage in the negative control (culture media alone or solvent control) at the indicated timepoint. Data were fitted with a four parameter [inhibitor] vs response curve in Prism 9, and the concentration inhibiting 50% of growth (IC_{50}) was calculated by the model.

References

1. G. R. Fulmer; A. J. M. Miller; N. H. Sherden; H. E. Gottlieb; A. Nudelman; B. M. Stoltz; J. E. Bercaw, ; K. I. Goldberg, NMR chemical shifts of trace impurities: common laboratory solvents, organics, and gases in deuterated solvents relevant to the organometallic chemist. *Organometallics* **29** (9), 2176–2179 (2010).

2. T. Yamauchi; M. Kato; T. Mikami; Y. Fujimura, Synthesis of 1-deoxymaxacalcitol. *Heterocycles* **65** (9), 2111 (2005).

3. V. M. A. Moreira; T. S. Vasaitis; Z. Guo; V. C. O. Njar; J. A. R. Salvador, Synthesis of novel C17 steroidal carbamates: studies on CYP17 action, androgen receptor binding and function, and prostate cancer cell growth. *Steroids* **73** (12), 1217–1227 (2008).

4. H. Hosoda; D. K. Fukushima; J. Fishman, Convenient, high yield conversion of androst-5-ene-3.beta.,17.beta.-diol to dehydroisoandrosterone. *J. Org. Chem.* **38** (24), 4209–4211 (1973).

5. A. Keglevich; V. Zsiros; P. Keglevich; Á. Szigetvári; M. Dékány; C. Szántay; E. Mernyák; J. Wölfling and L.Hazai, Synthesis and in vitro antitumor effect of new vindoline-steroid hybrids. *Curr. Org. Chem.* **23** (8), 959–967 (2019).

6. I. Fernandes; N. Vale; V. de Freitas; R. Moreira; N. Mateus; P. Gomes, Anti-tumoral activity of imidazoquines, a new class of antimalarials derived from primaquine. *Bioorg. Med. Chem. Lett.* **19** (24), 6914–6917 (2009).

7. T. Yamashita; N. Kawai; H. Tokuyama; T. Fukuyama, Stereocontrolled total synthesis of (−)-eudistomin C. *J. Am. Chem. Soc.* **127** (43), 15038–15039 (2005).

8. P. Kumar; D. S. Shankar Rao; S. Krishna Prasad; N. Jayaraman, In-plane modulated smectic Ã vs smectic 'A' lamellar structures in poly(ethyl or propyl ether imine) dendrimers. *Polymer* **86**, 98–104 (2016).

9. K. P. Cole; S. J. Ryan; J. M. Groh; R. D. Miller, Reagent-free continuous thermal tert-butyl ester deprotection. *Org. Synth. Flow Med. Chem.* **25** (23), 6209–6217 (2017).

10. B. Krausova; B. Slavikova; M. Nekardova; P. Hubalkova; V. Vyklicky; H. Chodounska; L. Vyklicky; E. Kudova, Positive modulators of the N-methyl-d-aspartate receptor: structure–activity relationship study of ateroidal 3-hemiesters. *J. Med. Chem.* **61** (10), 4505–4516 (2018).

11. T. Calogeropoulou; N. Avlonitis; V. Minas; X. Alexi; A. Pantzou; I. Charalampopoulos; M. Zervou; V. Vergou; E. S. Katsanou; I. Lazaridis; M. N. Alexis; A. Gravanis. Novel dehydroepiandrosterone derivatives with antiapoptotic, neuroprotective activity. *J. Med. Chem.* **52** (21), 6569–6587 (2009).

12. Y. Shen; D. L. Burgoyne, Efficient synthesis of IPL576,092: a novel anti-asthma agent. *J. Org. Chem.* **67** (11), 3908–3910 (2002).

13. R. B. Upsani; D. B. Fick; D. J. Hogenkamp; N. C. Lan, Neuroactive steroids of the androstane and pregnane series. US5925630A, July 20, 1999.

14. P. H. Dussault; H.-J. Lee; X. Liu, Selectivity in Lewis acid-mediated fragmentations of peroxides and ozonides: application to the synthesis of Alkenes, homoallyl ethers, and 1,2-dioxolanes. *J. Chem. Soc. Perkin 1* **17**, 3006–3013 (2000).

15. R. J. Havel, H. A. Eder, J. H. Bragdon, The distribution and chemical composition of ultracentrifugally separated lipoproteins in human serum. *Journal of Clinical Investigation* **34**, 1345–1353 (1955).

16. C. A. Schneider, W. S. Rasband, K. W. Eliceiri, NIH Image to ImageJ: 25 years of image analysis. *Nature Methods 2012 9:7* **9**, 671–675 (2012).

17. A. Maier, M. Rug, in *Malaria: Methods and Protocols*, R. Menard, Ed. (Humana Press, USA, 2013), pp. 3–15.

18. C. Lambros, J. P. Vanderberg, Synchronization of *Plasmodium falciparum* erythrocytic stages in culture. *The Journal of Parasitology* **65**, 418–420 (1979).

19. M. Smilkstein, N. Sriwilaijaroen, J. X. Kelly, P. Wilairat, M. Riscoe, Simple and inexpensive fluorescence-based technique for high-throughput antimalarial drug screening. *Antimicrob Agents Chemother* **48**, 1803–1806 (2004).

20. C. Spry, C. Macuamule, Z. Lin, K. G. Virga, R. E. Lee, E. Strauss, K. J. Saliba, S. A. Ralph, Ed. Pantothenamides are potent, on-target inhibitors of *Plasmodium falciparum* growth when serum pantetheinase is inactivated. *PLoS One* **8**, e54974 (2013).

21. M. C. Ridgway, K. S. Shea, D. Cihalova, A. G. Maier, Novel method for the separation of male and female gametocytes of the malaria parasite *Plasmodium falciparum* that enables biological and drug discovery. *mSphere* **5**, e00671-20 (2020).

22. T. W. A. Kooij, M. M. Rauch, K. Matuschewski, Expansion of experimental genetics approaches for *Plasmodium berghei* with versatile transfection vectors. *Mol Biochem Parasitol* **185**, 19–26 (2012).

23. C. J. Janse, B. Franke-Fayard, G. R. Mair, J. Ramesar, C. Thiel, S. Engelmann, K. Matuschewski, G. J. Van Gemert, R. W. Sauerwein, A. P. Waters, High efficiency transfection of *Plasmodium berghei* facilitates novel selection procedures. *Mol Biochem Parasitol* **145**, 60–70 (2006).

24. W. Petersen, W. Stenzel, O. Silvie, J. Blanz, P. Saftig, K. Matuschewski, A. Ingmundson, Sequestration of cholesterol within the host late endocytic pathway restricts liver-stage *Plasmodium* development. *Mol Biol Cell* **28**, 726–735 (2017).

25. J. Schindelin, I. Arganda-Carreras, E. Frise, V. Kaynig, M. Longair, T. Pietzsch, S. Preibisch, C. Rueden, S. Saalfeld, B. Schmid, J. Y. Tinevez, D. J. White, V. Hartenstein, K. Eliceiri, P. Tomancak, A. Cardona, Fiji - an open source platform for biological image analysis. *Nat Methods* **9**, 676–682 (2012).

26. J. A. Hayward, F. V. Makota, D. Cihalova, R. A. Leonard, E. Rajendran, S. M. Zwahlen, L. Shuttleworth, U. Wiedemann, C. Spry, K. J. Saliba, A. G. Maier, G. G. van Dooren, D. Soldati-Favre, Ed. A screen of drug-like molecules identifies chemically diverse electron transport chain inhibitors in apicomplexan parasites. *PLoS Pathog* **19**, e1011517 (2023).

27. J. Straimer, N. F. Gnädig, B. Witkowski, C. Amaratunga, V. Duru, A. P. Ramadani, M. Dacheux, N. Khim, L. Zhang, S. Lam, P. D. Gregory, F. D. Urnov, O. Mercereau-Puijalon, F. Benoit-Vical, R. M. Fairhurst, D. Ménard, D. A. Fidock, K13-propeller mutations confer artemisinin resistance in *Plasmodium falciparum* clinical isolates. *Science (1979)* **347**, 428–431 (2015).

28. B. Witkowski, D. Menard, C. Amaratunga, R. Fairhurst, Ring-stage Survival Assays (RSA) to evaluate the *in-vitro* and *ex-vivo* susceptibility of *Plasmodium falciparum* to artemisinins. *Institute Pasteur du Cambodge – National Institutes of Health* Procedure RSAv1 (2013).
