# Supplementary figures and images for: Harnessing cholesterol uptake of malaria parasites for therapeutic applications (part 1 of 2)
Source: EMBO Mol Med. 2024 Jun 11;16(7):4. doi: 10.1038/s44321-024-00087-1 (PMC11251039; doi:10.1038/s44321-024-00087-1)

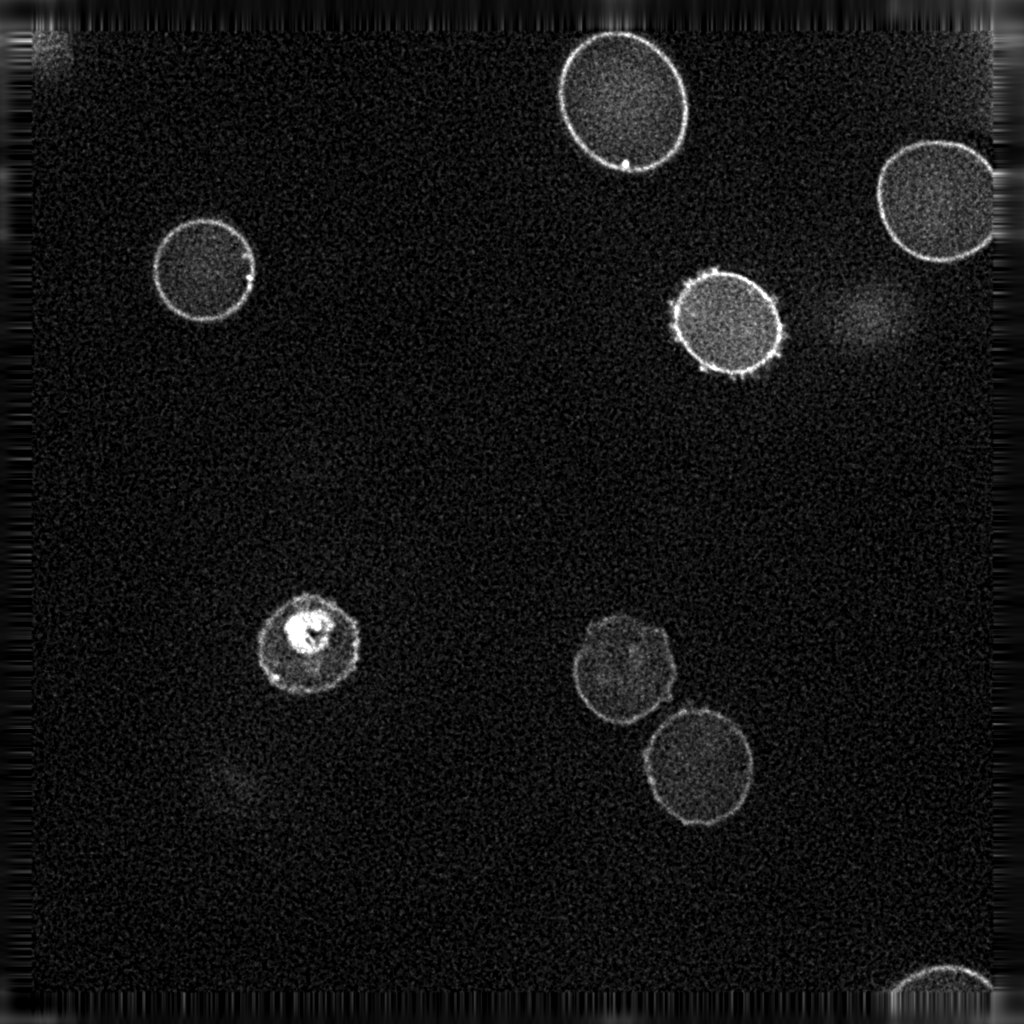

Supplement: Supplementary file 3 — Source data Fig. 1 [file 44321_2024_87_MOESM3_ESM.zip › Fig. 1/Figure 1F/Uncropped/1F_w523_Green.tif]

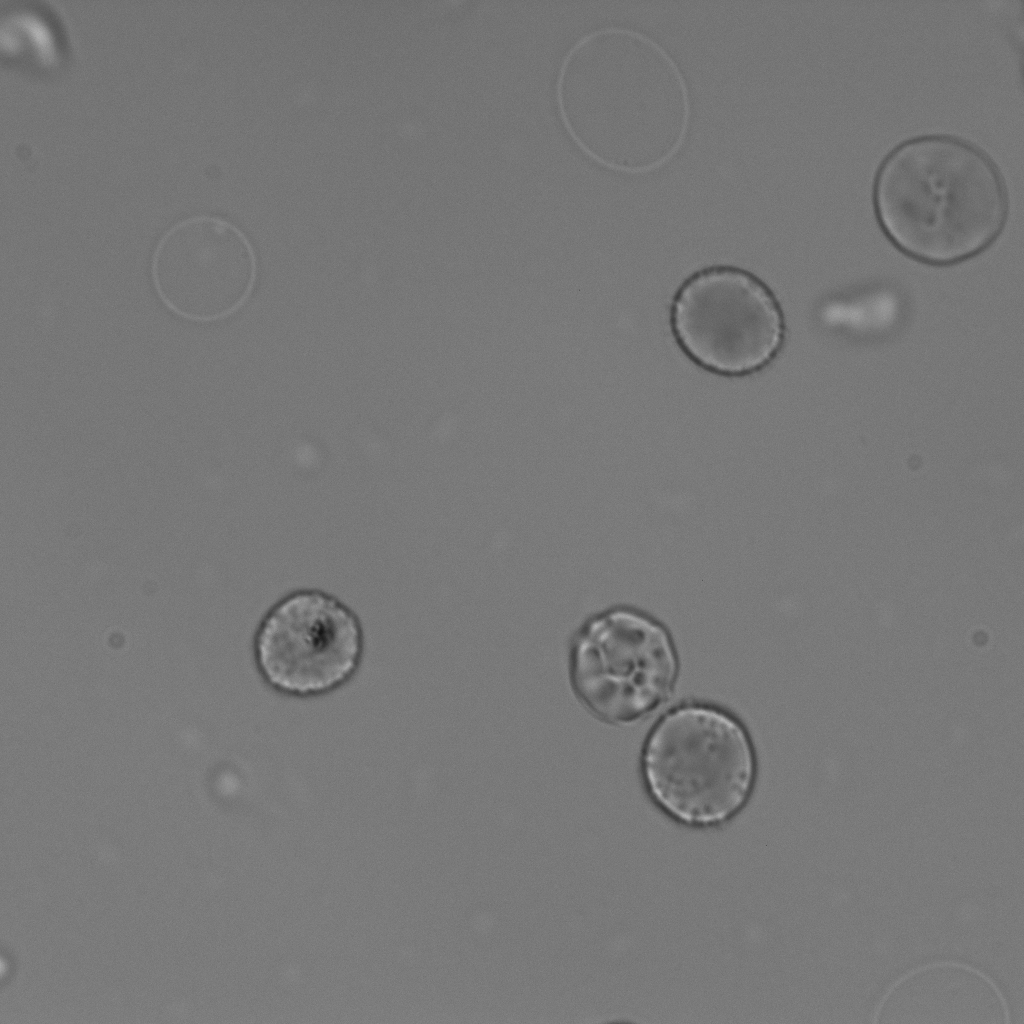

Supplement: Supplementary file 3 — Source data Fig. 1 [file 44321_2024_87_MOESM3_ESM.zip › Fig. 1/Figure 1F/Uncropped/1F_DIC.tif]

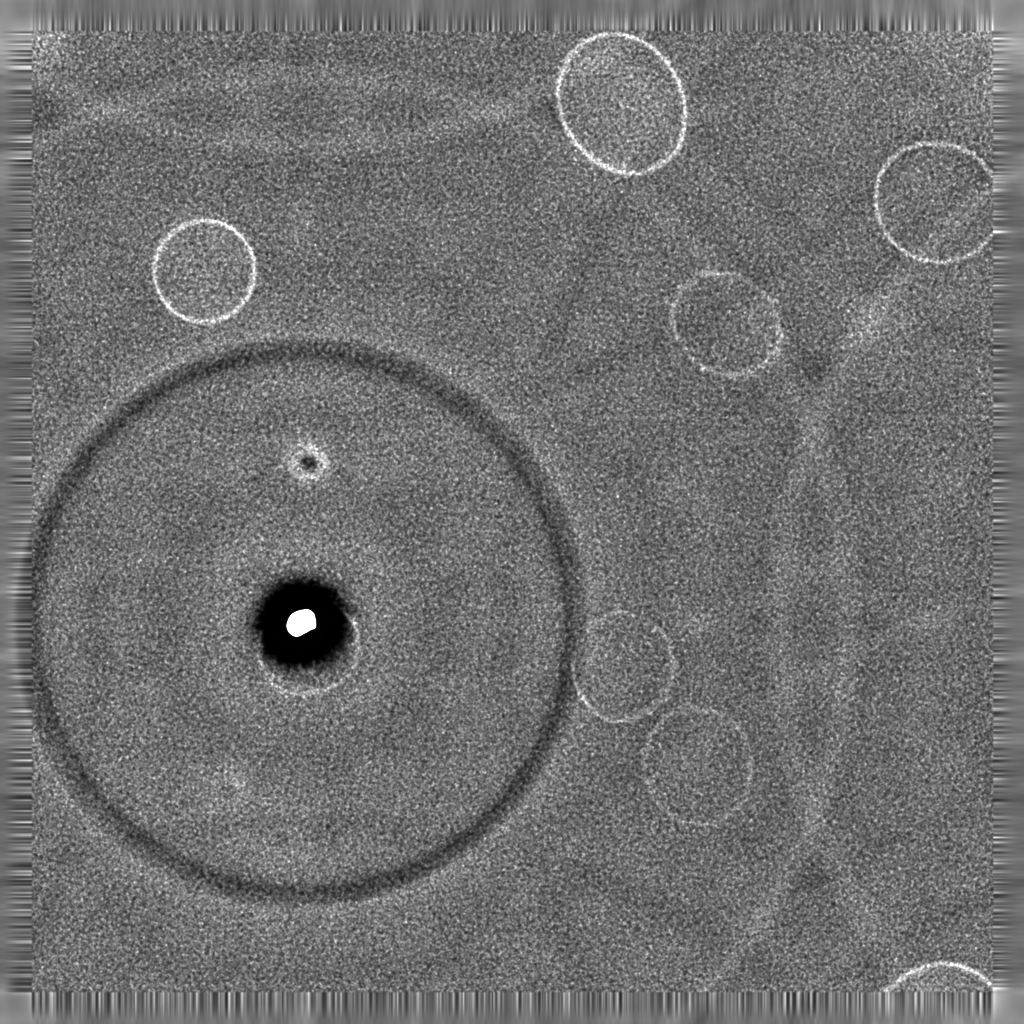

Supplement: Supplementary file 3 — Source data Fig. 1 [file 44321_2024_87_MOESM3_ESM.zip › Fig. 1/Figure 1F/Uncropped/1F_w435_Blue.tif]

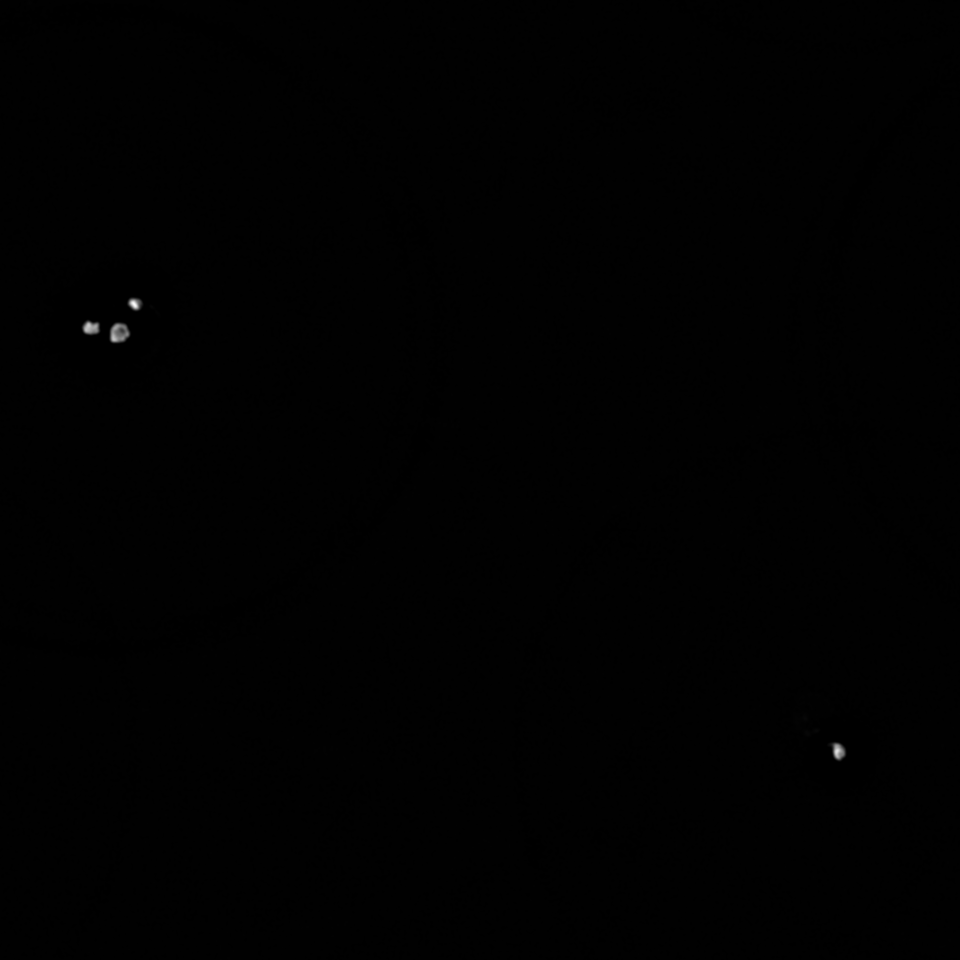

Supplement: Supplementary file 3 — Source data Fig. 1 [file 44321_2024_87_MOESM3_ESM.zip › Fig. 1/Figure 1G/Uncropped/1G_w435_Blue.tif]

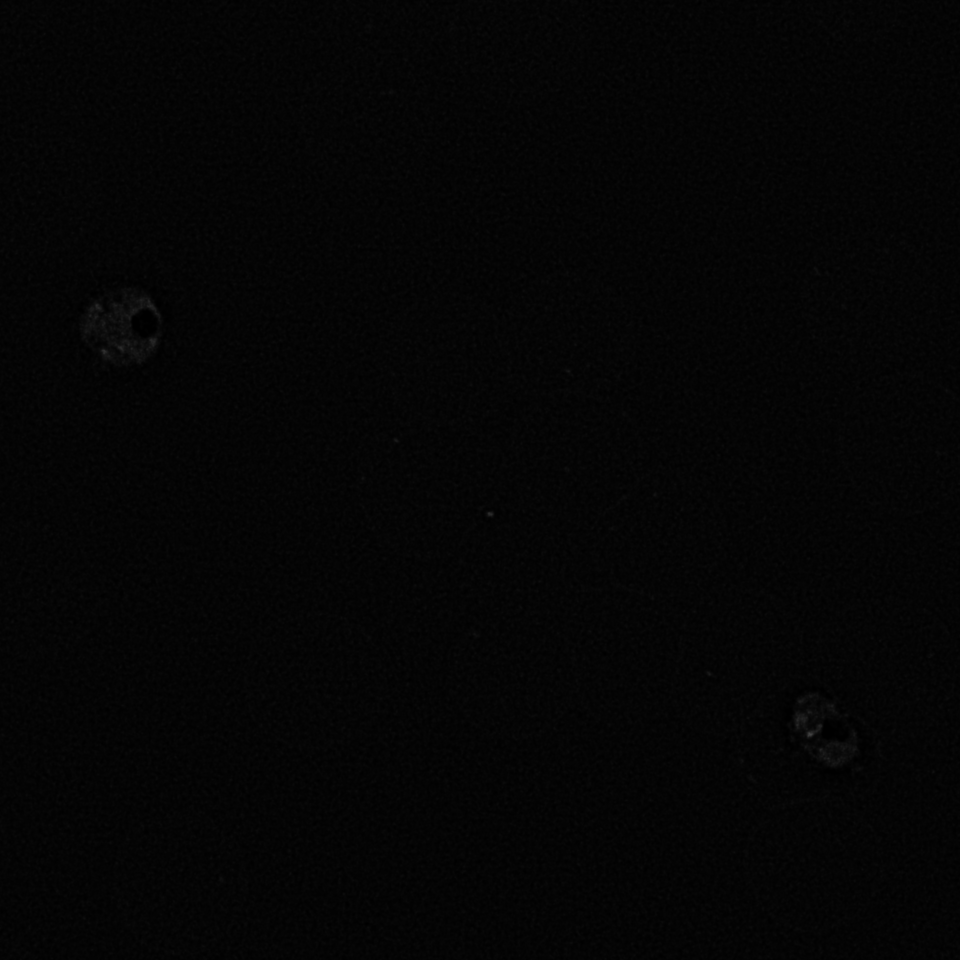

Supplement: Supplementary file 3 — Source data Fig. 1 [file 44321_2024_87_MOESM3_ESM.zip › Fig. 1/Figure 1G/Uncropped/1G_w523_Green.tif]

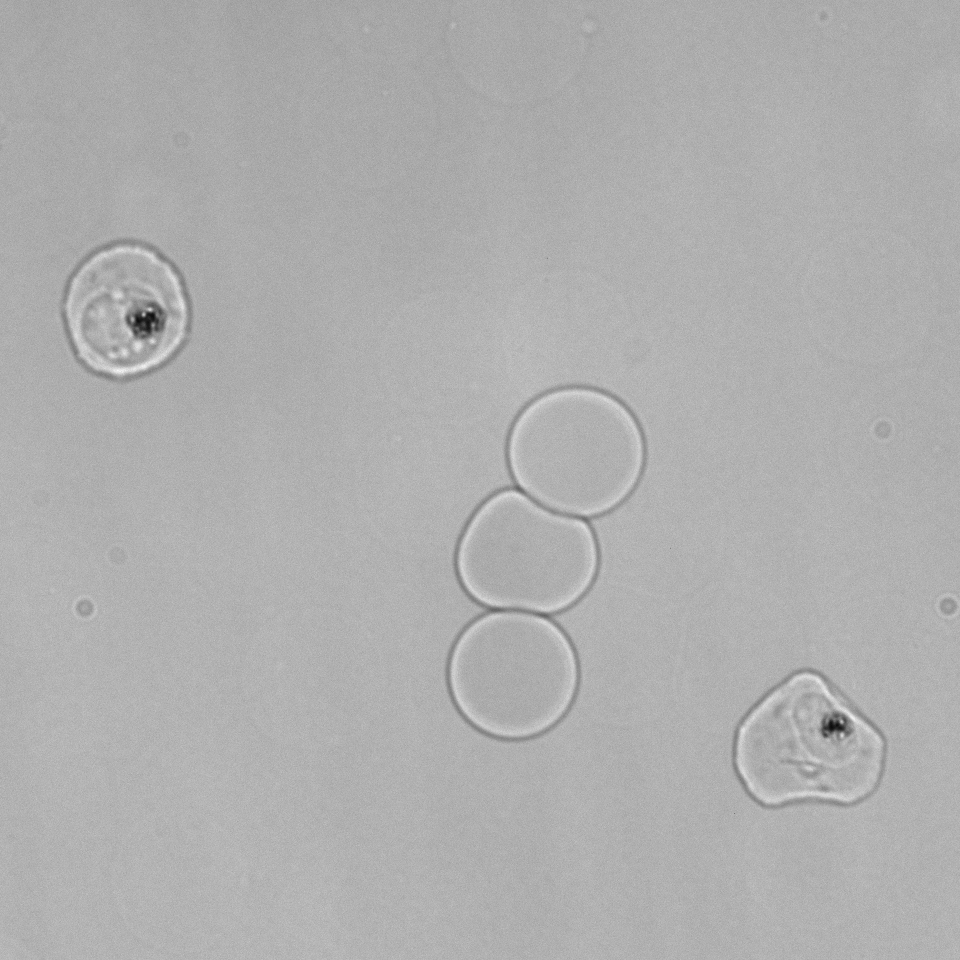

Supplement: Supplementary file 3 — Source data Fig. 1 [file 44321_2024_87_MOESM3_ESM.zip › Fig. 1/Figure 1G/Uncropped/1G_DIC.tif]

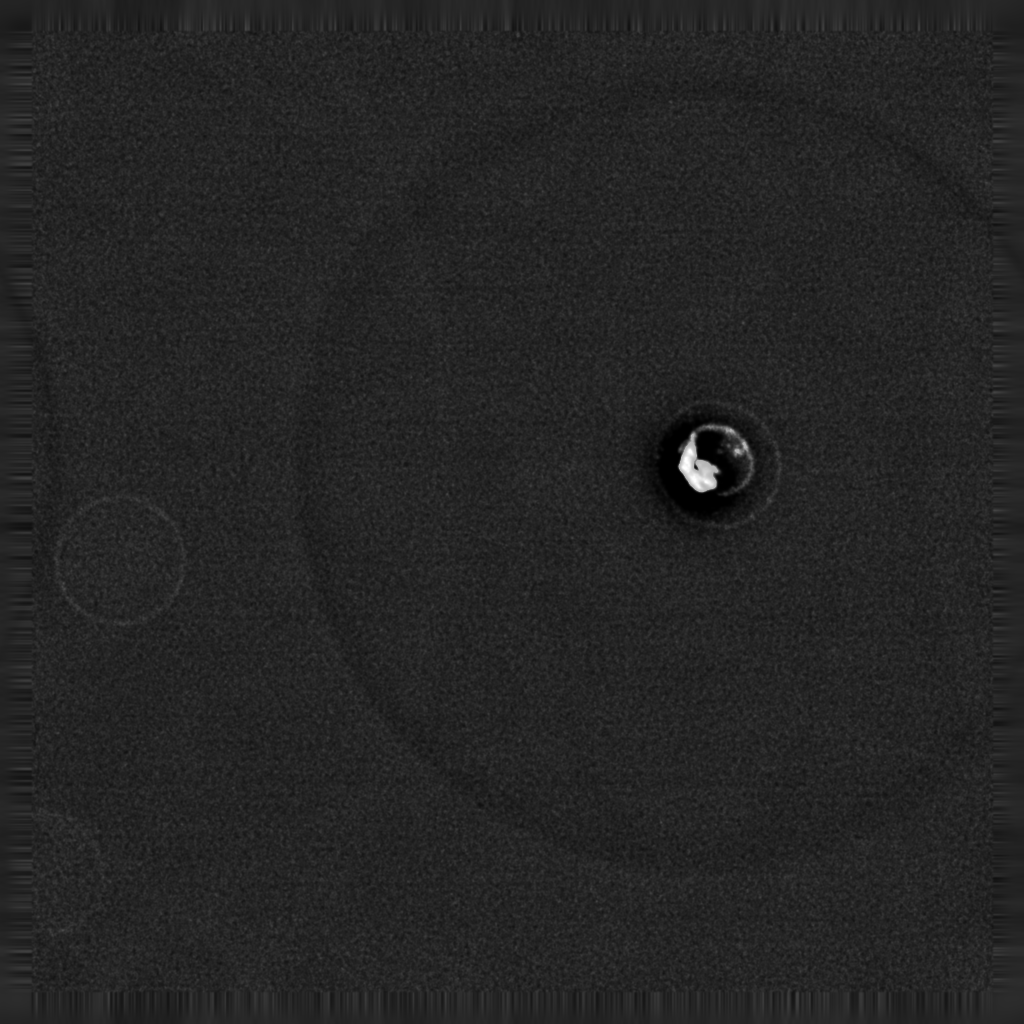

Supplement: Supplementary file 3 — Source data Fig. 1 [file 44321_2024_87_MOESM3_ESM.zip › Fig. 1/Figure 1D/Uncropped/1D_w435_Blue.tif]

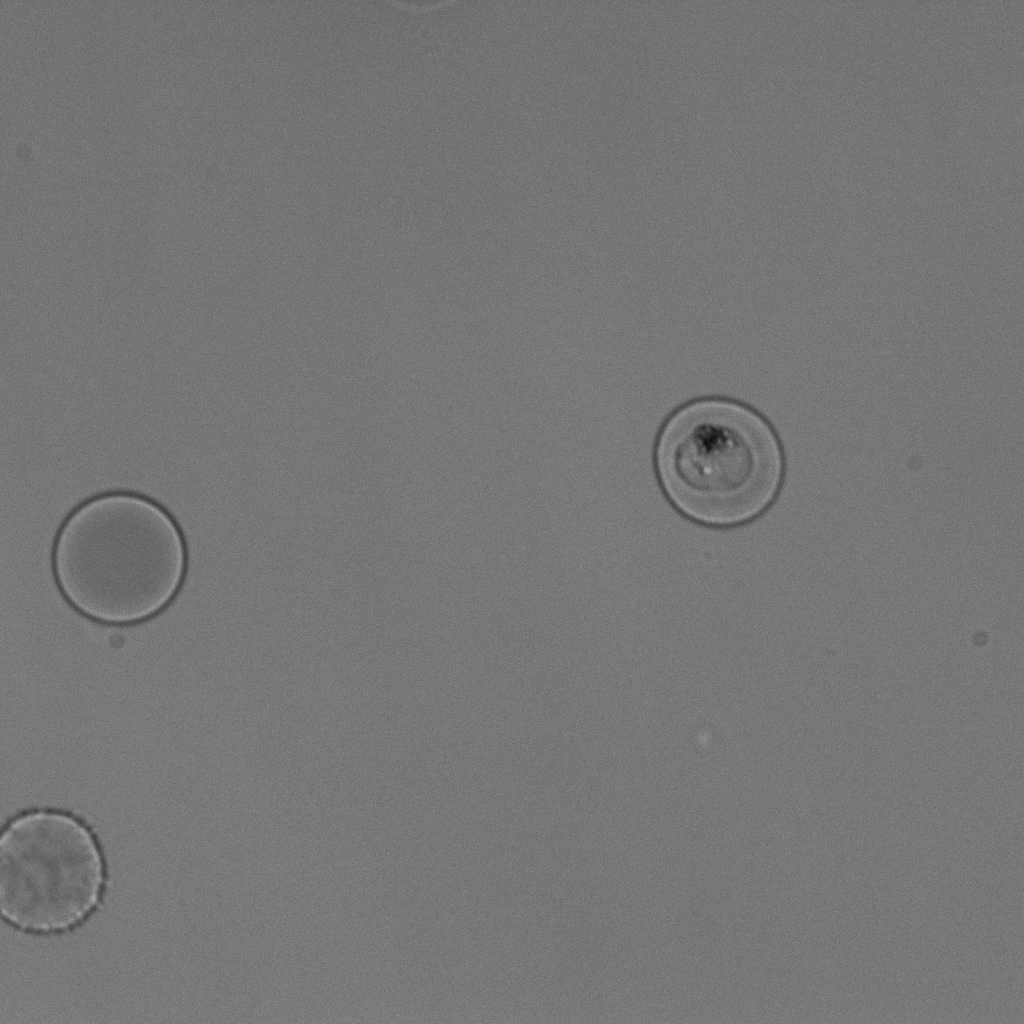

Supplement: Supplementary file 3 — Source data Fig. 1 [file 44321_2024_87_MOESM3_ESM.zip › Fig. 1/Figure 1D/Uncropped/1D_DIC.tif]

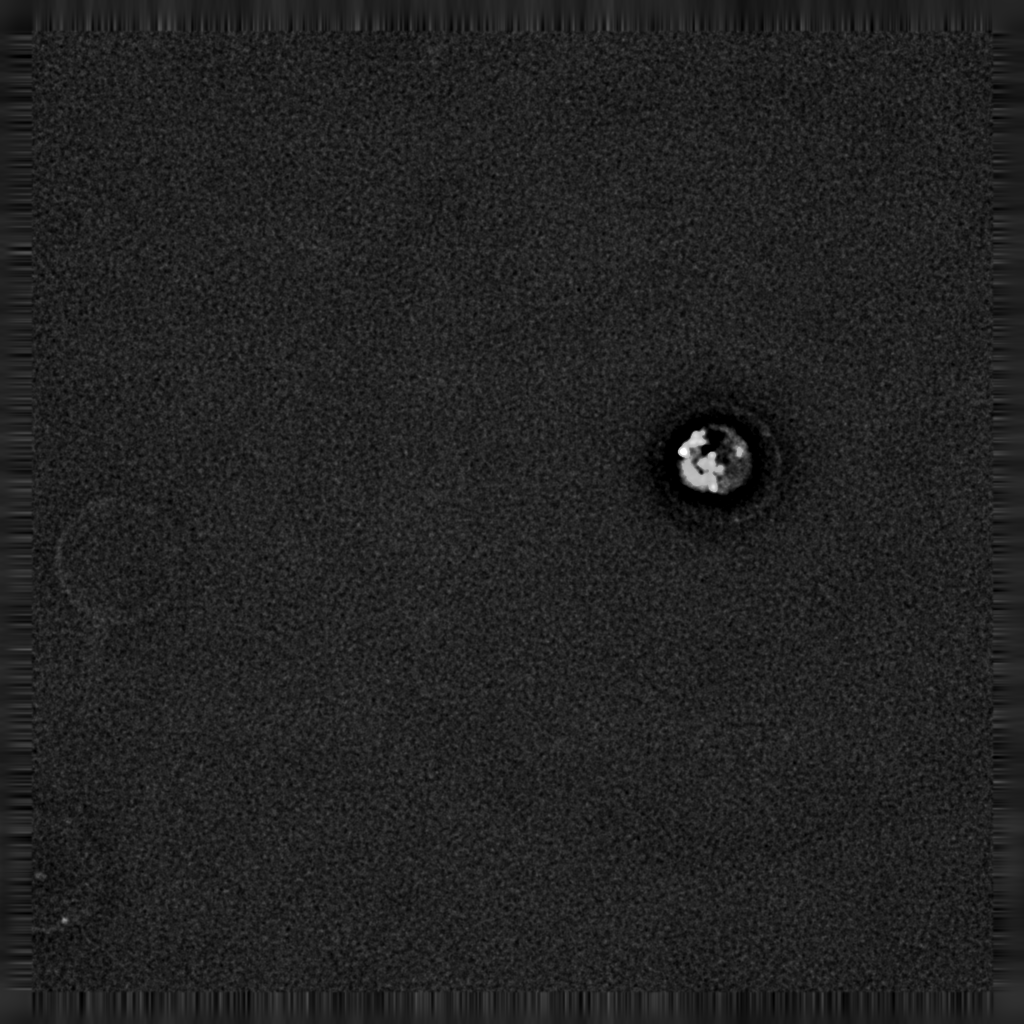

Supplement: Supplementary file 3 — Source data Fig. 1 [file 44321_2024_87_MOESM3_ESM.zip › Fig. 1/Figure 1D/Uncropped/1D_w523_Green.tif]

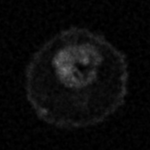

Supplement: Supplementary file 3 — Source data Fig. 1 [file 44321_2024_87_MOESM3_ESM.zip › Fig. 1/Figure 1F/Cropped/Infected/1F_w523_Green_Infected.tif]

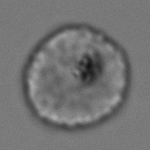

Supplement: Supplementary file 3 — Source data Fig. 1 [file 44321_2024_87_MOESM3_ESM.zip › Fig. 1/Figure 1F/Cropped/Infected/1F_DIC_Infected.tif]

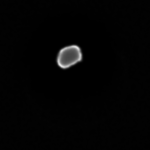

Supplement: Supplementary file 3 — Source data Fig. 1 [file 44321_2024_87_MOESM3_ESM.zip › Fig. 1/Figure 1F/Cropped/Infected/1F_w435_Blue_Infected.tif]

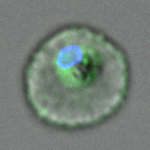

Supplement: Supplementary file 3 — Source data Fig. 1 [file 44321_2024_87_MOESM3_ESM.zip › Fig. 1/Figure 1F/Cropped/Infected/1F_Merge_Infected.tif]

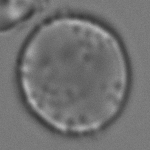

Supplement: Supplementary file 3 — Source data Fig. 1 [file 44321_2024_87_MOESM3_ESM.zip › Fig. 1/Figure 1F/Cropped/Uninfected/1F_DIC_Uninfected.tif]

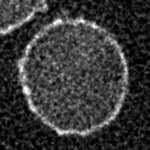

Supplement: Supplementary file 3 — Source data Fig. 1 [file 44321_2024_87_MOESM3_ESM.zip › Fig. 1/Figure 1F/Cropped/Uninfected/1F_w523_Green_Uninfected.tif]

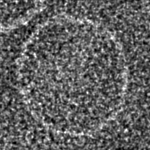

Supplement: Supplementary file 3 — Source data Fig. 1 [file 44321_2024_87_MOESM3_ESM.zip › Fig. 1/Figure 1F/Cropped/Uninfected/1F_w435_Blue_Uninfected.tif]

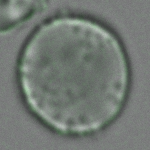

Supplement: Supplementary file 3 — Source data Fig. 1 [file 44321_2024_87_MOESM3_ESM.zip › Fig. 1/Figure 1F/Cropped/Uninfected/1F_Merge_Uninfected.tif]

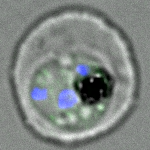

Supplement: Supplementary file 3 — Source data Fig. 1 [file 44321_2024_87_MOESM3_ESM.zip › Fig. 1/Figure 1G/Cropped/Infected/1G_Merge_Infected.tif]

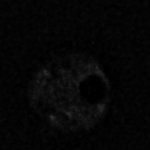

Supplement: Supplementary file 3 — Source data Fig. 1 [file 44321_2024_87_MOESM3_ESM.zip › Fig. 1/Figure 1G/Cropped/Infected/1G_w523_Green_Infected.tif]

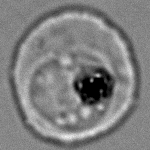

Supplement: Supplementary file 3 — Source data Fig. 1 [file 44321_2024_87_MOESM3_ESM.zip › Fig. 1/Figure 1G/Cropped/Infected/1G_DIC_Infected.tif]

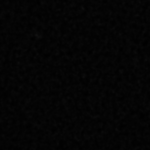

Supplement: Supplementary file 3 — Source data Fig. 1 [file 44321_2024_87_MOESM3_ESM.zip › Fig. 1/Figure 1G/Cropped/Uninfected/1G_w523_Green_Uninfected.tif]

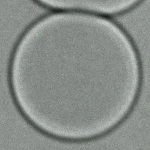

Supplement: Supplementary file 3 — Source data Fig. 1 [file 44321_2024_87_MOESM3_ESM.zip › Fig. 1/Figure 1G/Cropped/Uninfected/1G_Merge_Uninfected.tif]

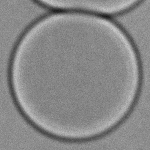

Supplement: Supplementary file 3 — Source data Fig. 1 [file 44321_2024_87_MOESM3_ESM.zip › Fig. 1/Figure 1G/Cropped/Uninfected/1G_DIC_Uninfected.tif]

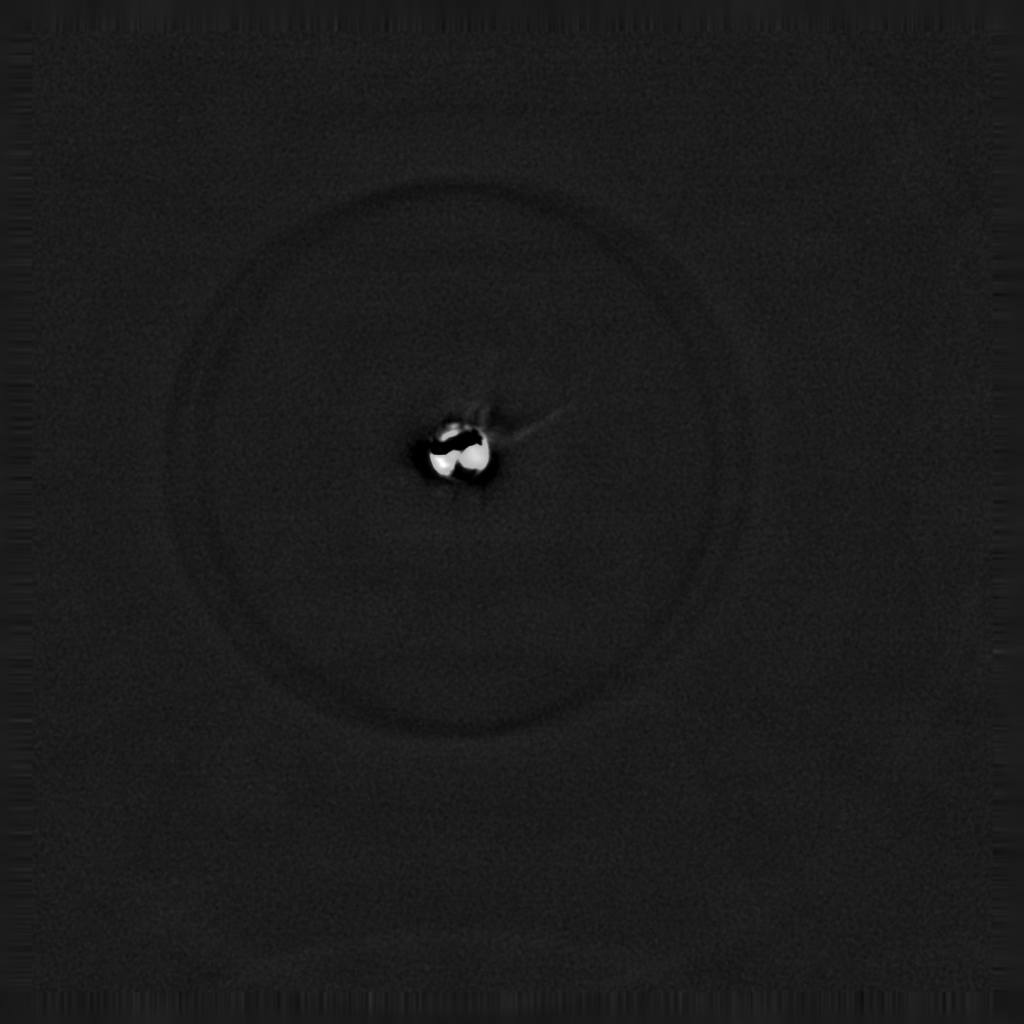

Supplement: Supplementary file 3 — Source data Fig. 1 [file 44321_2024_87_MOESM3_ESM.zip › Fig. 1/Figure 1B/Uncropped/Infected/1B_w435_Green_Infected.tif]

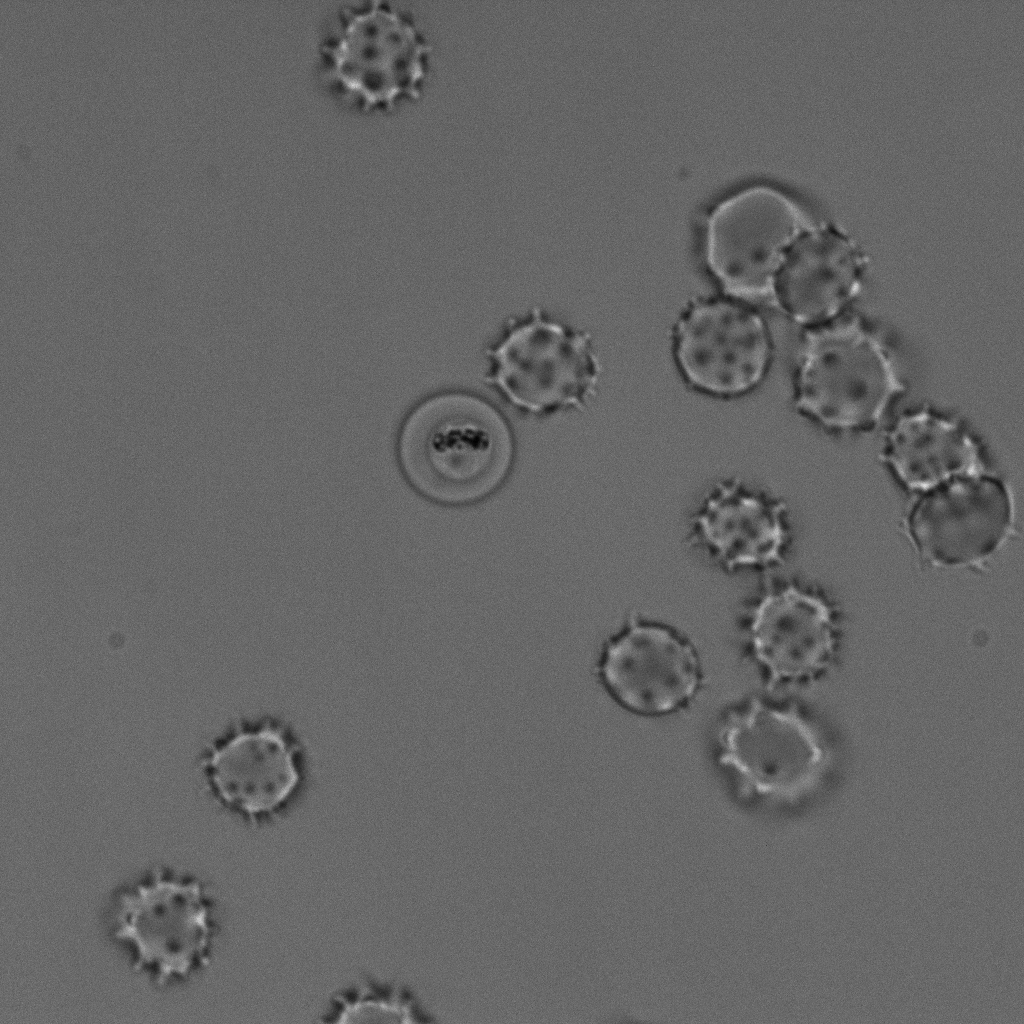

Supplement: Supplementary file 3 — Source data Fig. 1 [file 44321_2024_87_MOESM3_ESM.zip › Fig. 1/Figure 1B/Uncropped/Infected/1B_DIC_Infected.tif]

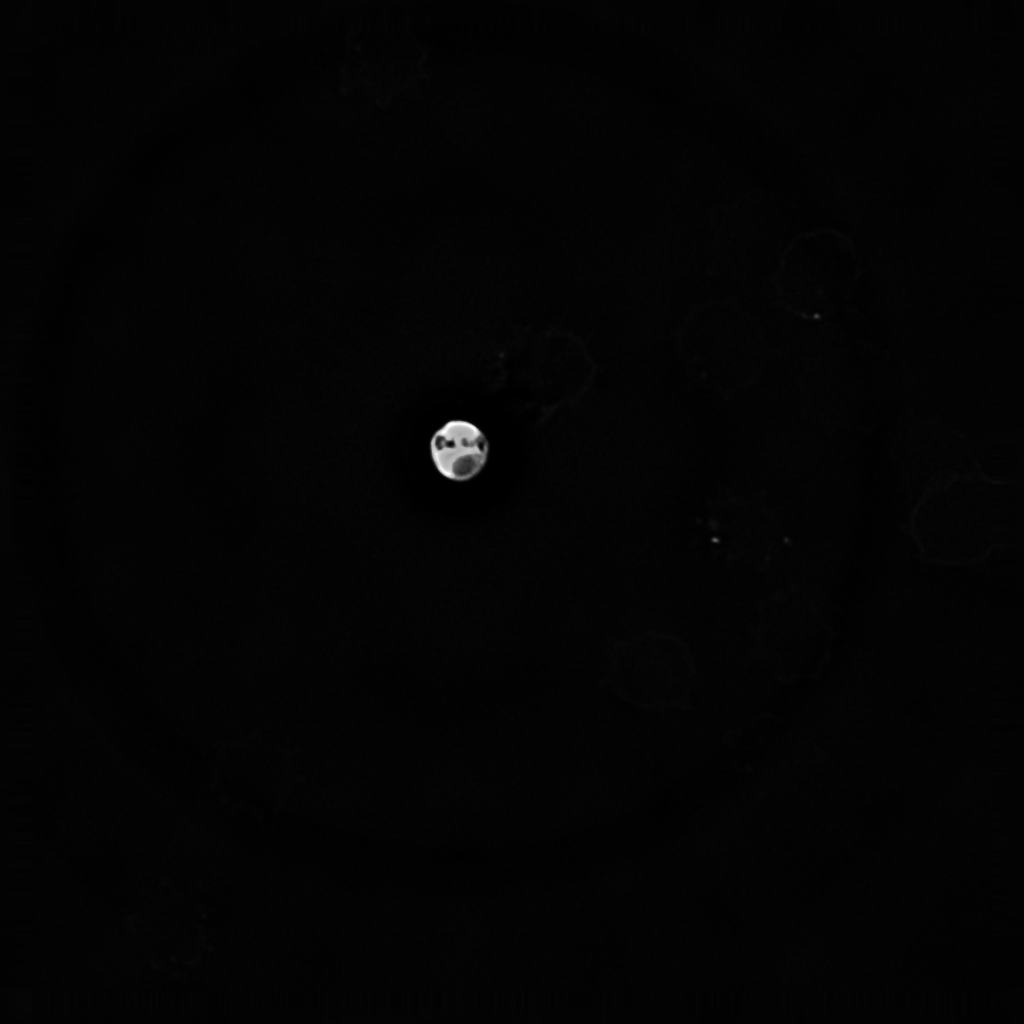

Supplement: Supplementary file 3 — Source data Fig. 1 [file 44321_2024_87_MOESM3_ESM.zip › Fig. 1/Figure 1B/Uncropped/Infected/1B_w676_Red_Infected.tif]

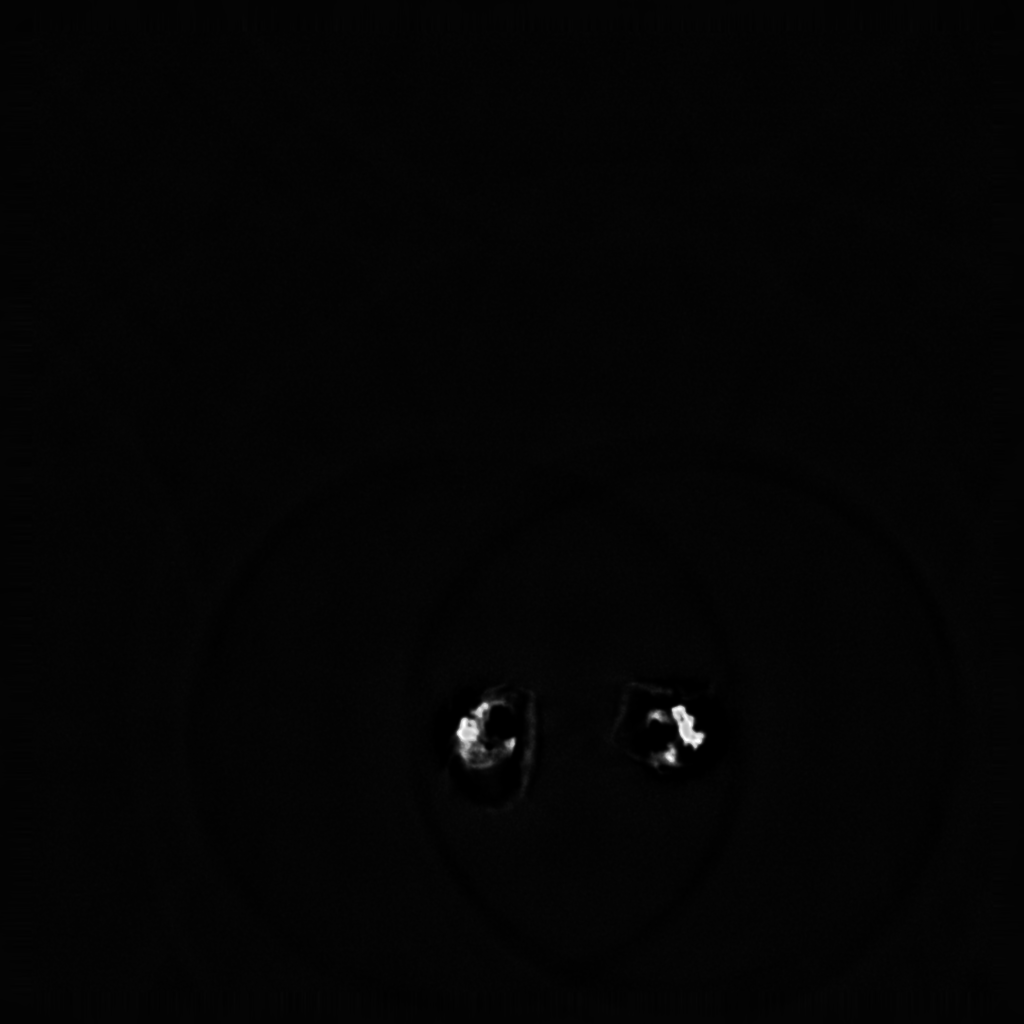

Supplement: Supplementary file 3 — Source data Fig. 1 [file 44321_2024_87_MOESM3_ESM.zip › Fig. 1/Figure 1B/Uncropped/Uninfected/1B_w435_Green_Uninfected.tif]

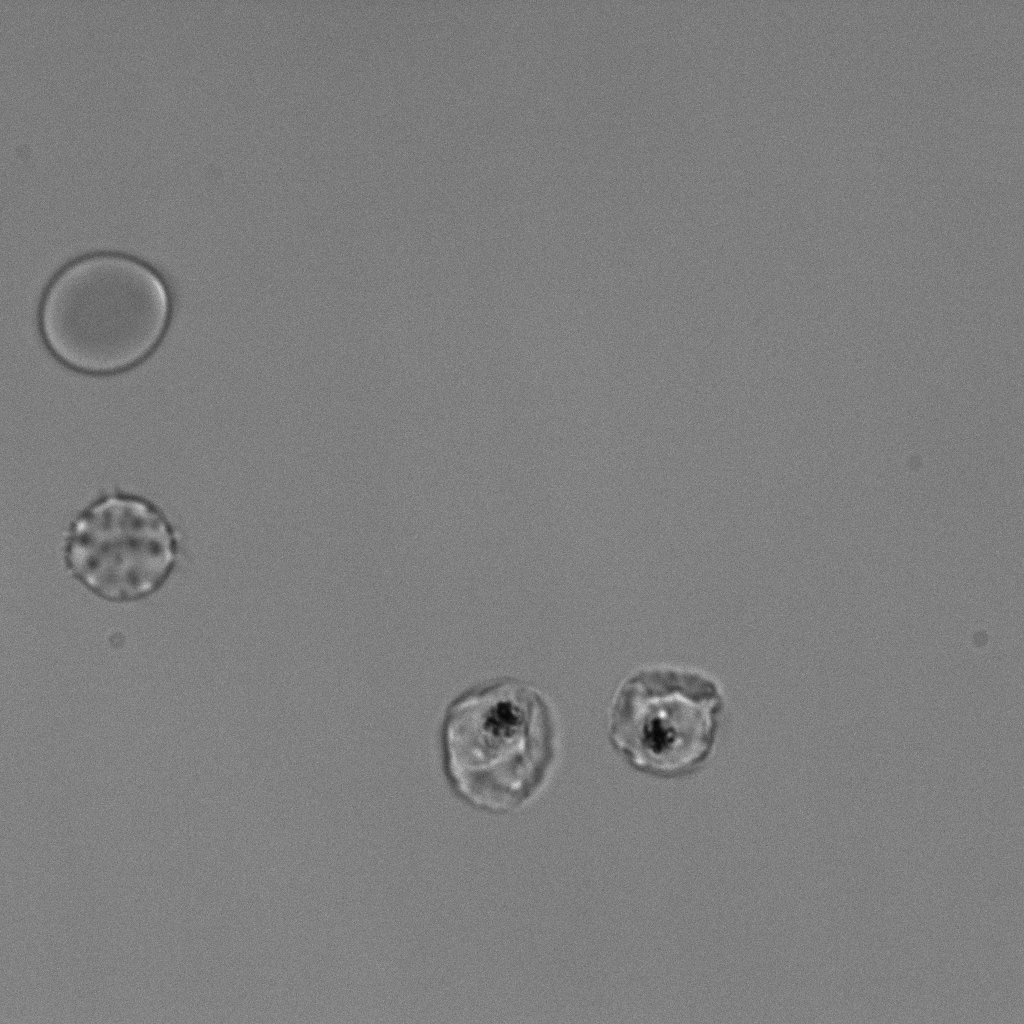

Supplement: Supplementary file 3 — Source data Fig. 1 [file 44321_2024_87_MOESM3_ESM.zip › Fig. 1/Figure 1B/Uncropped/Uninfected/1B_DIC_Uninfected.tif]

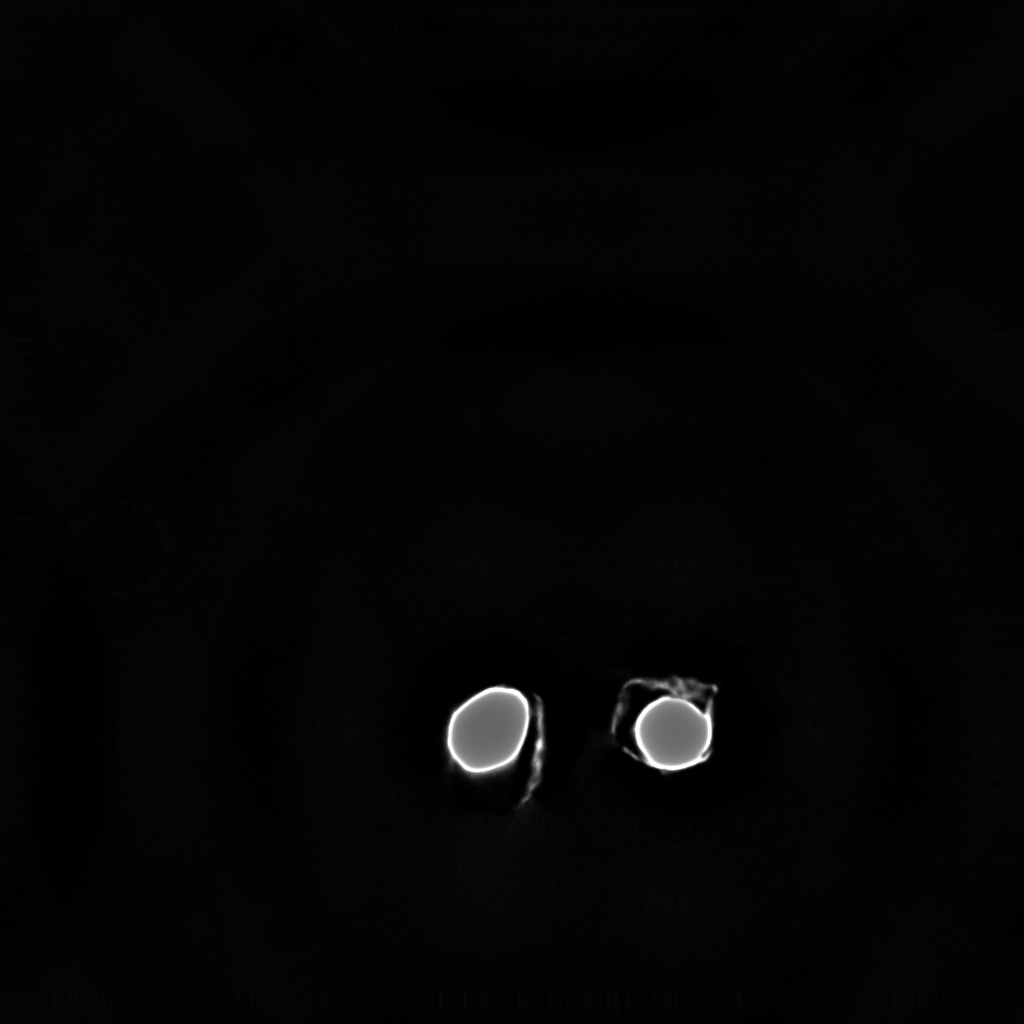

Supplement: Supplementary file 3 — Source data Fig. 1 [file 44321_2024_87_MOESM3_ESM.zip › Fig. 1/Figure 1B/Uncropped/Uninfected/1B_w676_Red_Uninfected.tif]

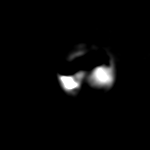

Supplement: Supplementary file 3 — Source data Fig. 1 [file 44321_2024_87_MOESM3_ESM.zip › Fig. 1/Figure 1B/Cropped/Infected/1B_w435_Green_Infected.tif]

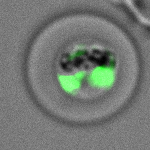

Supplement: Supplementary file 3 — Source data Fig. 1 [file 44321_2024_87_MOESM3_ESM.zip › Fig. 1/Figure 1B/Cropped/Infected/1B_Merge_Infected.tif]

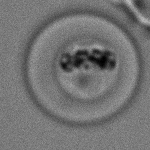

Supplement: Supplementary file 3 — Source data Fig. 1 [file 44321_2024_87_MOESM3_ESM.zip › Fig. 1/Figure 1B/Cropped/Infected/1B_DIC_Infected.tif]

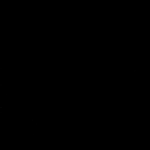

Supplement: Supplementary file 3 — Source data Fig. 1 [file 44321_2024_87_MOESM3_ESM.zip › Fig. 1/Figure 1B/Cropped/Uninfected/1B_w435_Green_Uninfected.tif]

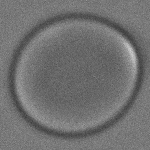

Supplement: Supplementary file 3 — Source data Fig. 1 [file 44321_2024_87_MOESM3_ESM.zip › Fig. 1/Figure 1B/Cropped/Uninfected/1B_DIC_Uninfected copy.tif]

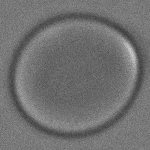

Supplement: Supplementary file 3 — Source data Fig. 1 [file 44321_2024_87_MOESM3_ESM.zip › Fig. 1/Figure 1B/Cropped/Uninfected/1B_Merge_Uninfected.tif]

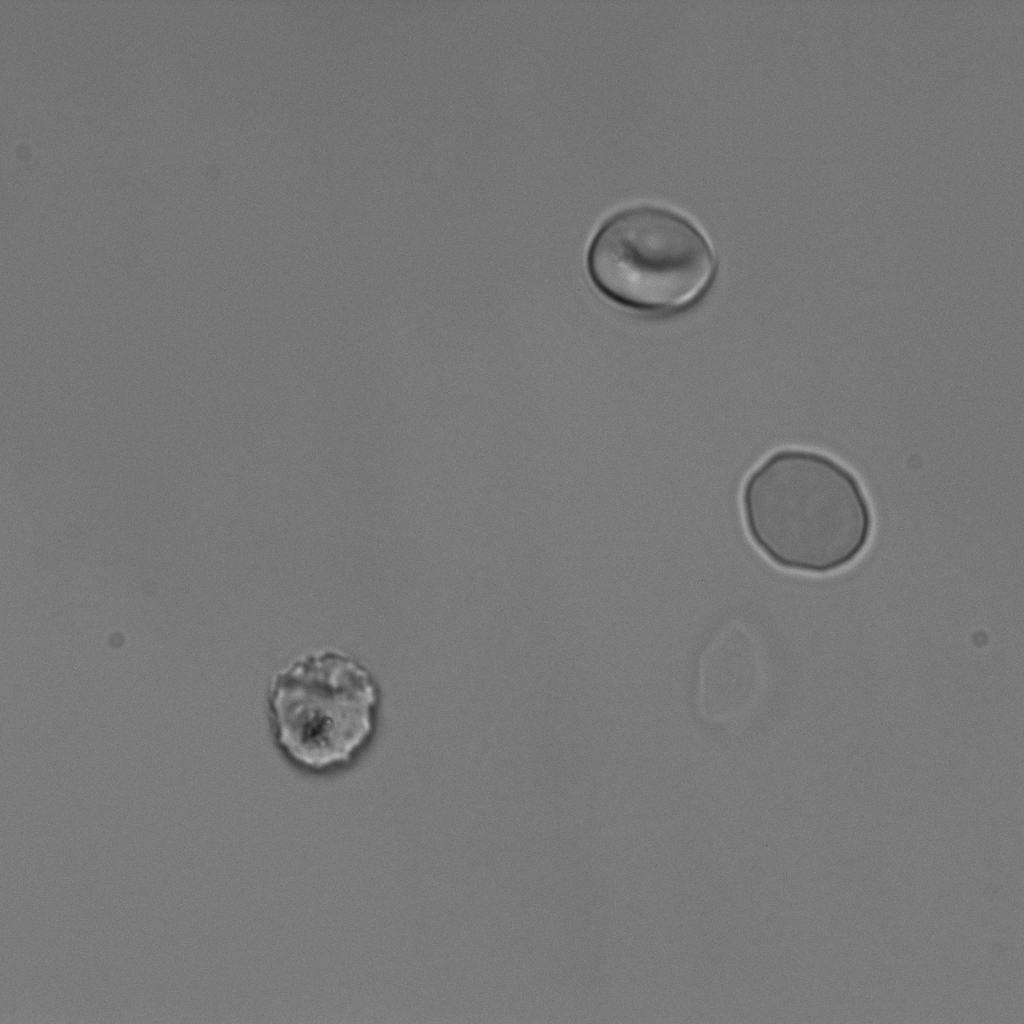

Supplement: Supplementary file 3 — Source data Fig. 1 [file 44321_2024_87_MOESM3_ESM.zip › Fig. 1/Figure 1E/Uncropped/Infected/1E_DIC_Infected.tif]

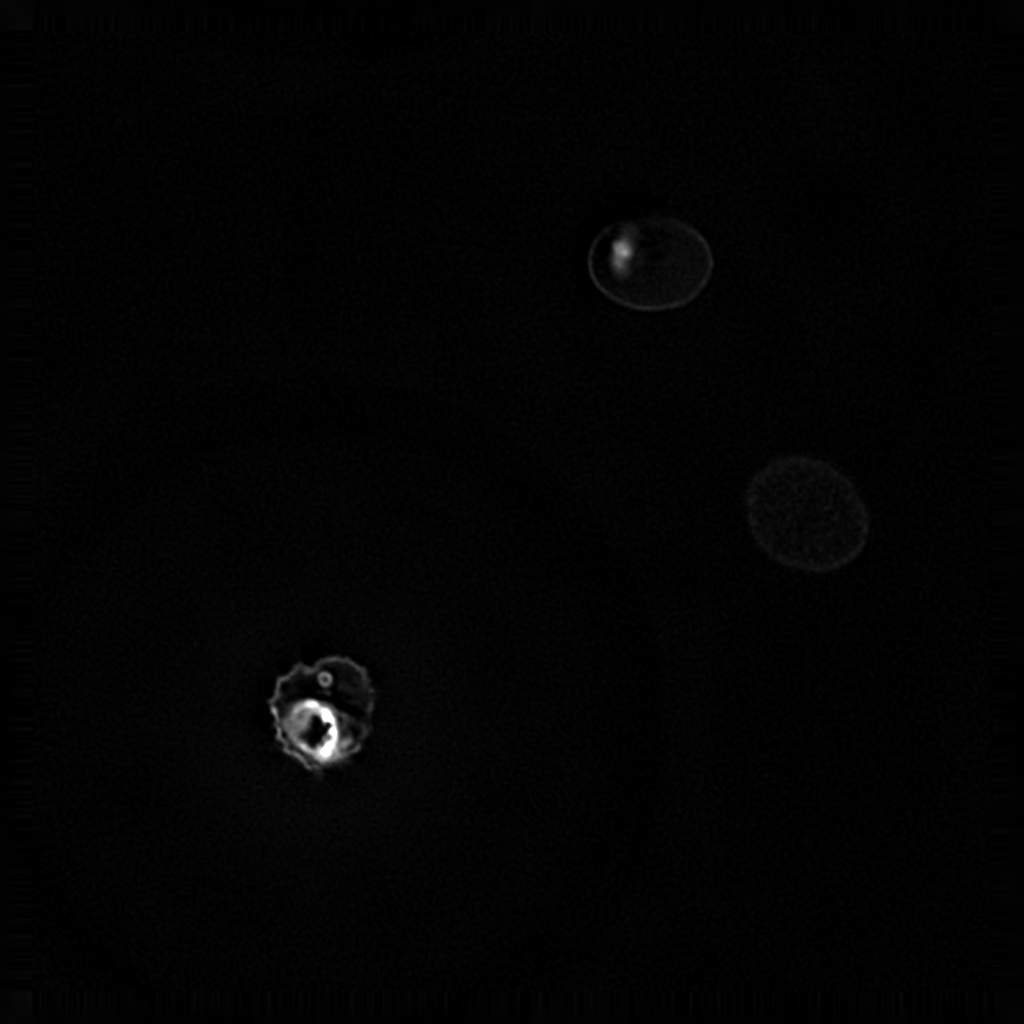

Supplement: Supplementary file 3 — Source data Fig. 1 [file 44321_2024_87_MOESM3_ESM.zip › Fig. 1/Figure 1E/Uncropped/Infected/1E_w523_Green_Infected.tif]

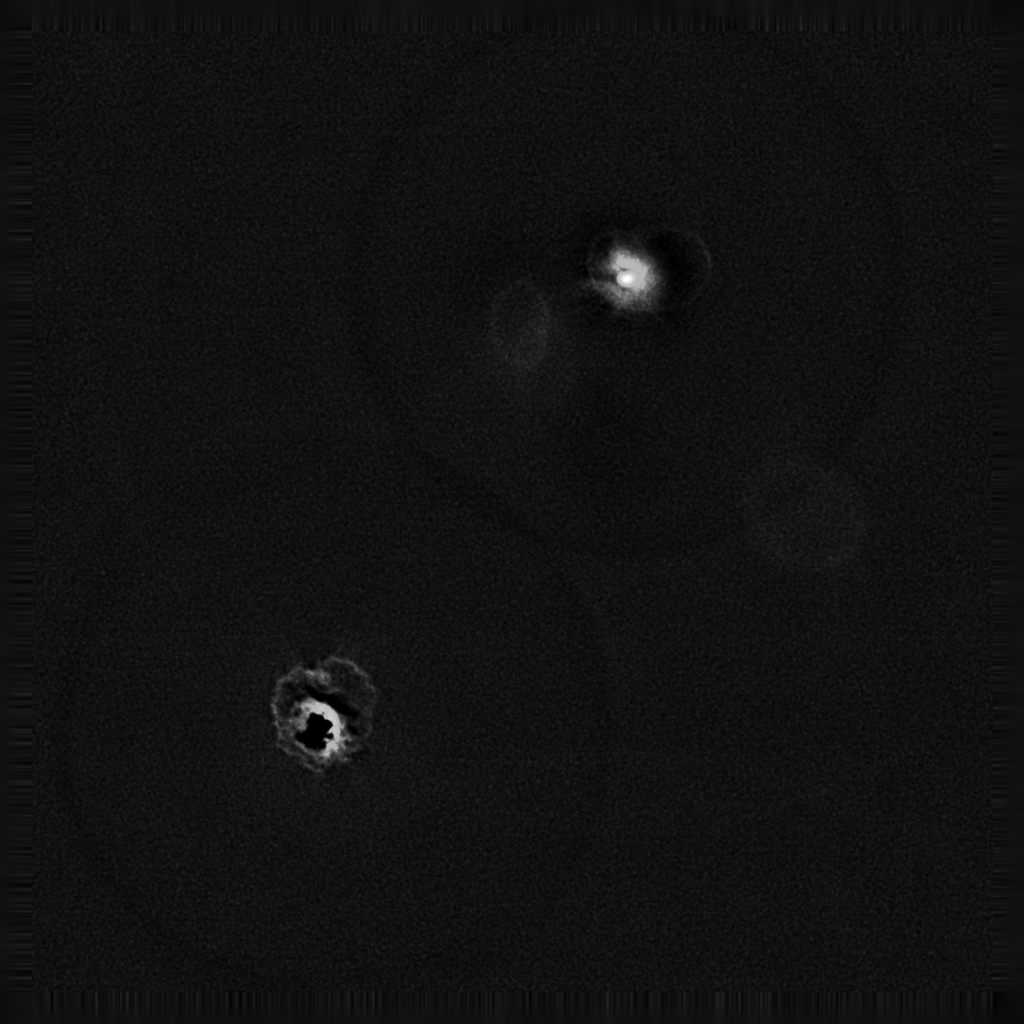

Supplement: Supplementary file 3 — Source data Fig. 1 [file 44321_2024_87_MOESM3_ESM.zip › Fig. 1/Figure 1E/Uncropped/Infected/1E_w435_Blue_Infected.tif]

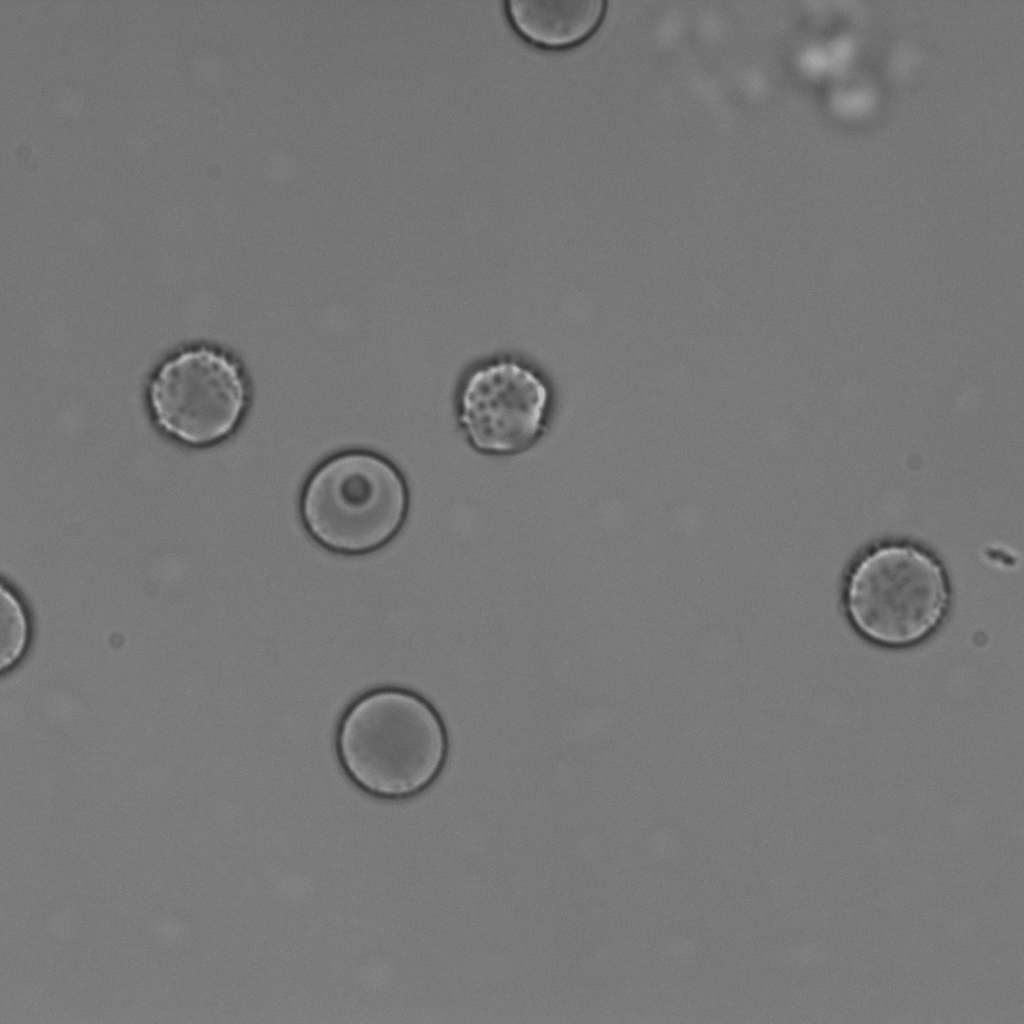

Supplement: Supplementary file 3 — Source data Fig. 1 [file 44321_2024_87_MOESM3_ESM.zip › Fig. 1/Figure 1E/Uncropped/Uninfected/1E_DIC_Uninfected.tif]

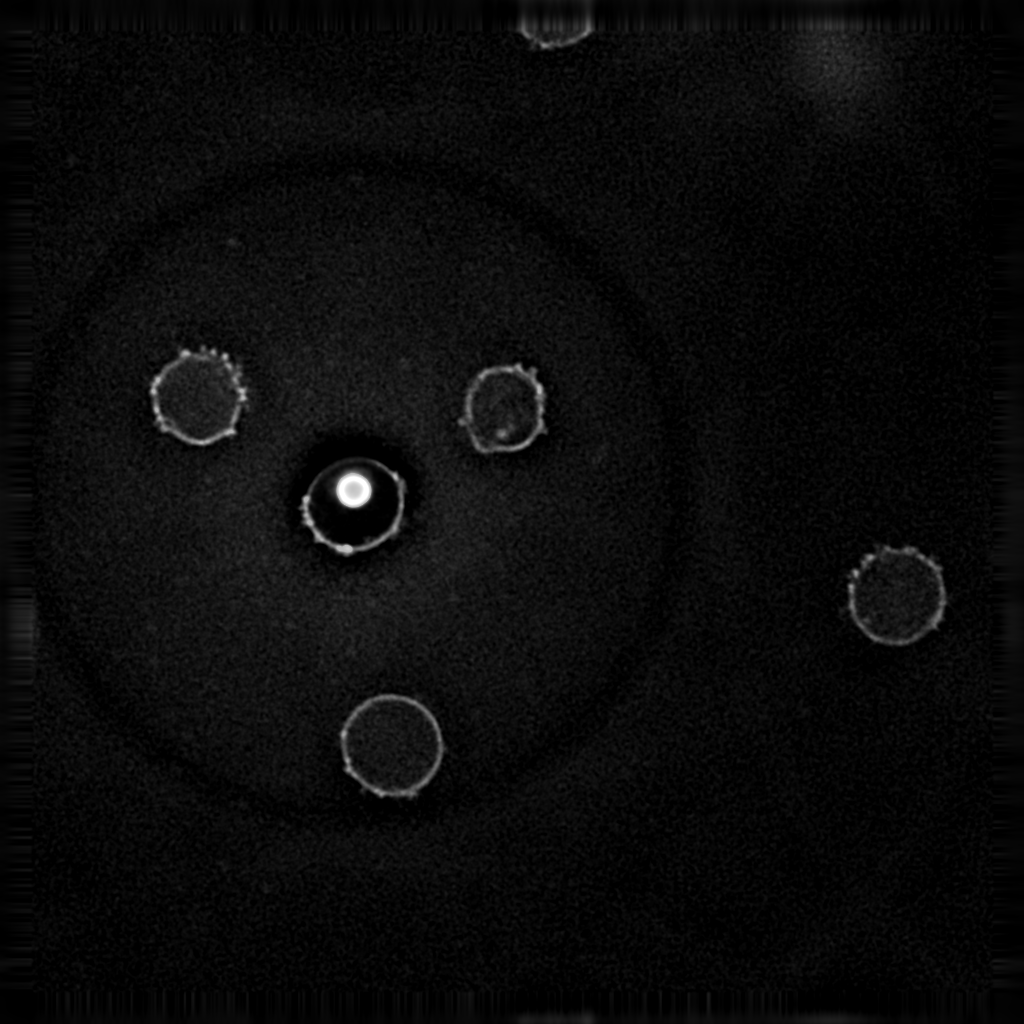

Supplement: Supplementary file 3 — Source data Fig. 1 [file 44321_2024_87_MOESM3_ESM.zip › Fig. 1/Figure 1E/Uncropped/Uninfected/1E_w523_Green_Uninfected.tif]

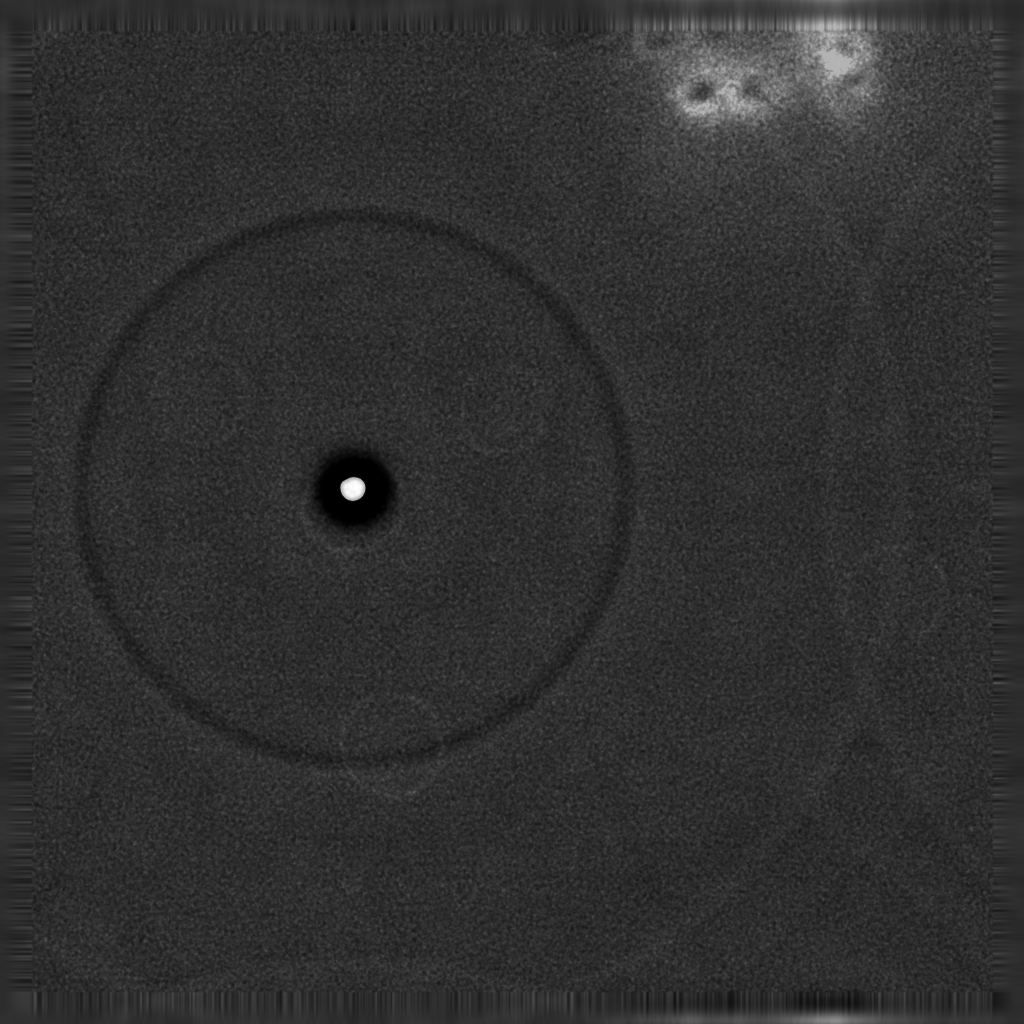

Supplement: Supplementary file 3 — Source data Fig. 1 [file 44321_2024_87_MOESM3_ESM.zip › Fig. 1/Figure 1E/Uncropped/Uninfected/1E_w435_Blue_Uninfected.tif]

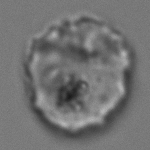

Supplement: Supplementary file 3 — Source data Fig. 1 [file 44321_2024_87_MOESM3_ESM.zip › Fig. 1/Figure 1E/Cropped/Infected/1E_DIC_Infected.tif]

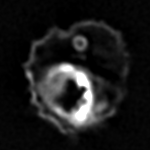

Supplement: Supplementary file 3 — Source data Fig. 1 [file 44321_2024_87_MOESM3_ESM.zip › Fig. 1/Figure 1E/Cropped/Infected/1E_w523_Green_Infected.tif]

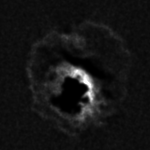

Supplement: Supplementary file 3 — Source data Fig. 1 [file 44321_2024_87_MOESM3_ESM.zip › Fig. 1/Figure 1E/Cropped/Infected/1E_w435_Blue_Infected.tif]

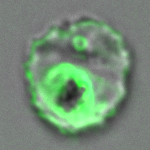

Supplement: Supplementary file 3 — Source data Fig. 1 [file 44321_2024_87_MOESM3_ESM.zip › Fig. 1/Figure 1E/Cropped/Infected/1E_Merge_Infected.tif]

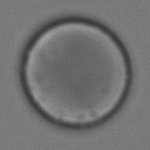

Supplement: Supplementary file 3 — Source data Fig. 1 [file 44321_2024_87_MOESM3_ESM.zip › Fig. 1/Figure 1E/Cropped/Uninfected/1E_DIC_Uninfected.tif]

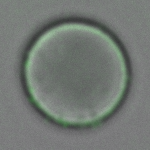

Supplement: Supplementary file 3 — Source data Fig. 1 [file 44321_2024_87_MOESM3_ESM.zip › Fig. 1/Figure 1E/Cropped/Uninfected/1E_Merge_Uninfected.tif]

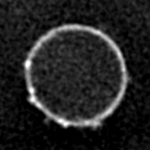

Supplement: Supplementary file 3 — Source data Fig. 1 [file 44321_2024_87_MOESM3_ESM.zip › Fig. 1/Figure 1E/Cropped/Uninfected/1E_w523_Green_Uninfected.tif]

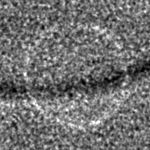

Supplement: Supplementary file 3 — Source data Fig. 1 [file 44321_2024_87_MOESM3_ESM.zip › Fig. 1/Figure 1E/Cropped/Uninfected/1E_w435_Blue_Uninfected.tif]

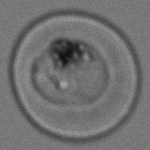

Supplement: Supplementary file 3 — Source data Fig. 1 [file 44321_2024_87_MOESM3_ESM.zip › Fig. 1/Figure 1D/Cropped/Infected/1D_DIC_Infected.tif]

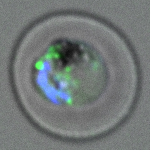

Supplement: Supplementary file 3 — Source data Fig. 1 [file 44321_2024_87_MOESM3_ESM.zip › Fig. 1/Figure 1D/Cropped/Infected/1D_Merge_Infected.tif]

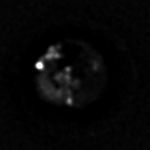

Supplement: Supplementary file 3 — Source data Fig. 1 [file 44321_2024_87_MOESM3_ESM.zip › Fig. 1/Figure 1D/Cropped/Infected/1D_w523_Green_Infected.tif]

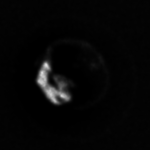

Supplement: Supplementary file 3 — Source data Fig. 1 [file 44321_2024_87_MOESM3_ESM.zip › Fig. 1/Figure 1D/Cropped/Infected/1D_w435_Blue_Infected.tif]

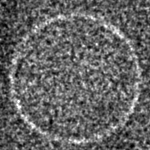

Supplement: Supplementary file 3 — Source data Fig. 1 [file 44321_2024_87_MOESM3_ESM.zip › Fig. 1/Figure 1D/Cropped/Uninfected/1D_w435_Blue_Uninfected.tif]

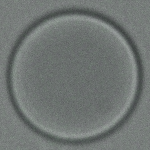

Supplement: Supplementary file 3 — Source data Fig. 1 [file 44321_2024_87_MOESM3_ESM.zip › Fig. 1/Figure 1D/Cropped/Uninfected/1D_Merge_Uninfected.tif]

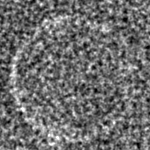

Supplement: Supplementary file 3 — Source data Fig. 1 [file 44321_2024_87_MOESM3_ESM.zip › Fig. 1/Figure 1D/Cropped/Uninfected/1D_w523_Green_Uninfected.tif]

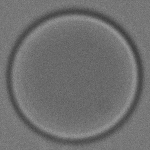

Supplement: Supplementary file 3 — Source data Fig. 1 [file 44321_2024_87_MOESM3_ESM.zip › Fig. 1/Figure 1D/Cropped/Uninfected/1D_DIC_Uninfected.tif]

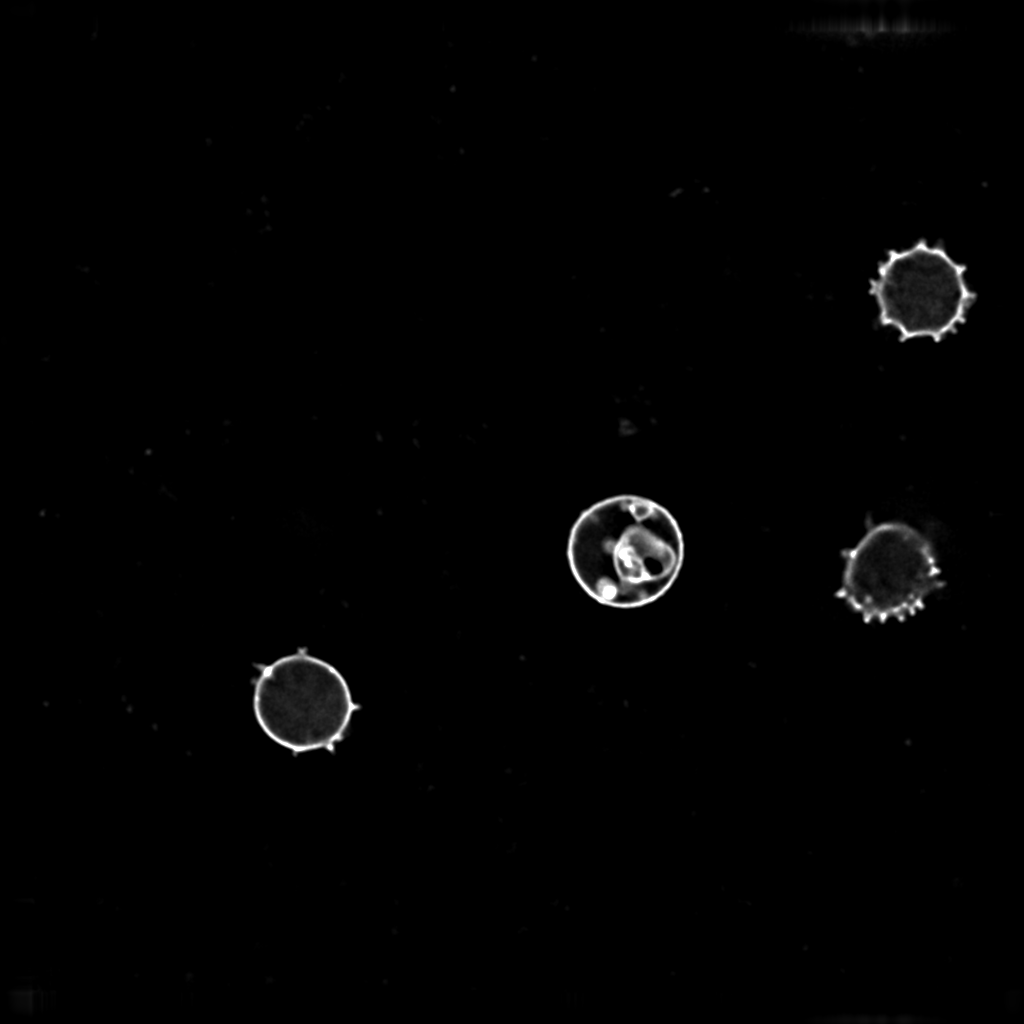

Supplement: Supplementary file 3 — Source data Fig. 1 [file 44321_2024_87_MOESM3_ESM.zip › Fig. 1/Figure 1C/Uncropped/Infected/1C_w523_Green_INfected.tif]

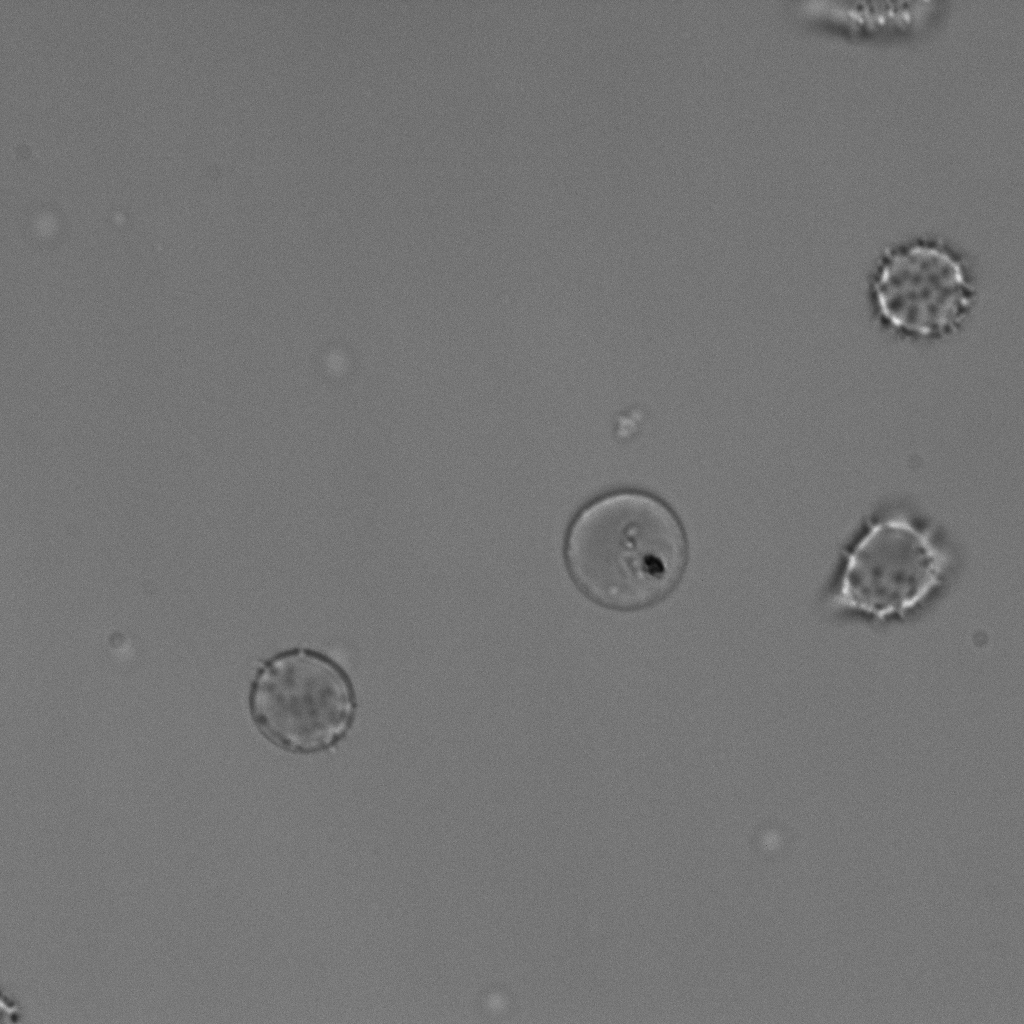

Supplement: Supplementary file 3 — Source data Fig. 1 [file 44321_2024_87_MOESM3_ESM.zip › Fig. 1/Figure 1C/Uncropped/Infected/1C_DIC_Infected.tif]

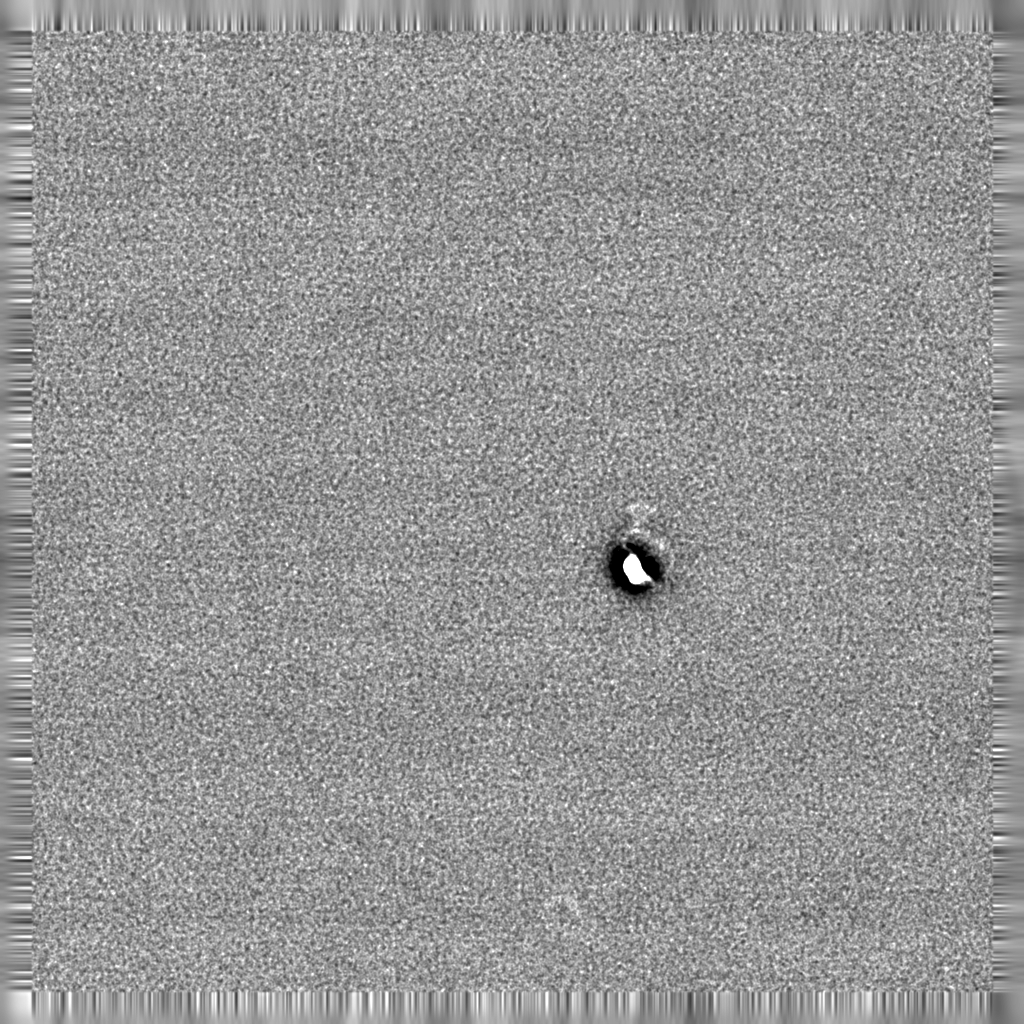

Supplement: Supplementary file 3 — Source data Fig. 1 [file 44321_2024_87_MOESM3_ESM.zip › Fig. 1/Figure 1C/Uncropped/Infected/1C_w435_Blue_Infected.tif]

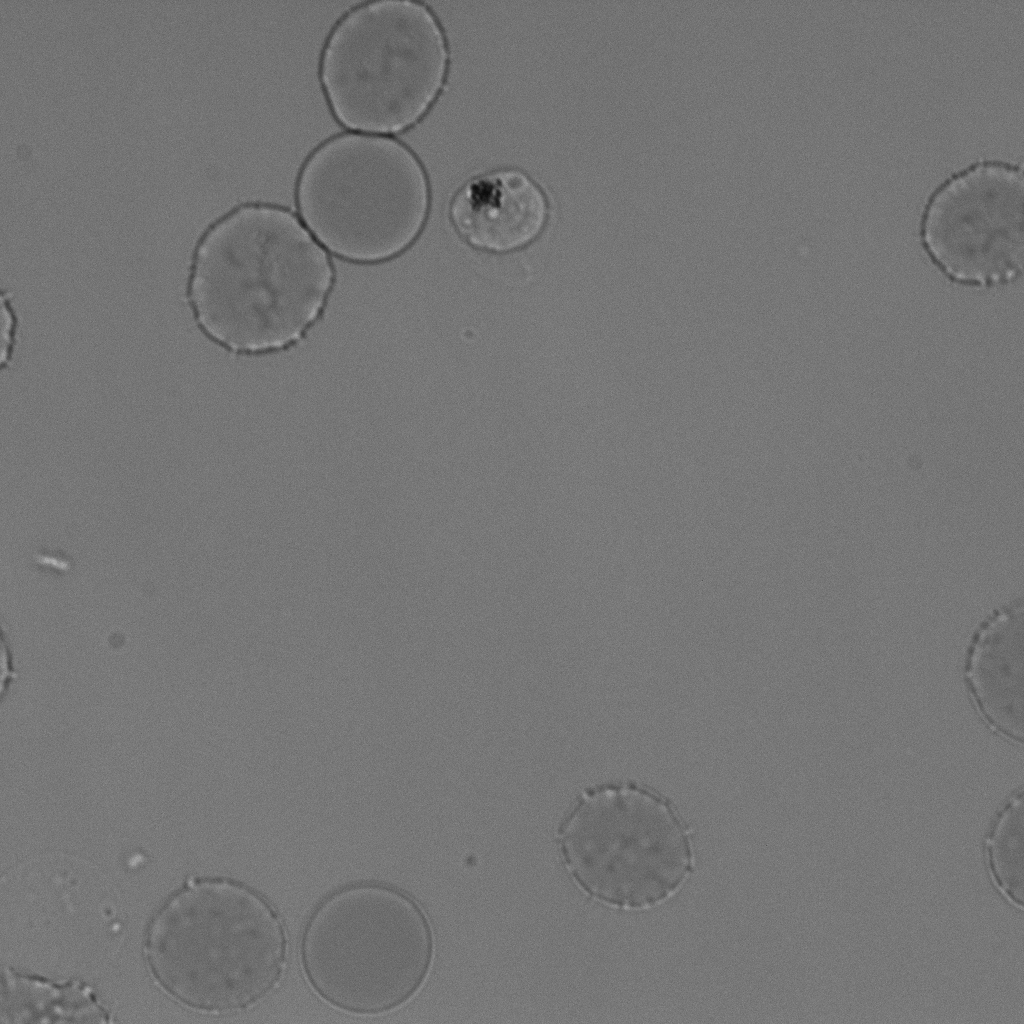

Supplement: Supplementary file 3 — Source data Fig. 1 [file 44321_2024_87_MOESM3_ESM.zip › Fig. 1/Figure 1C/Uncropped/Uninfected/1C_DIC_Uninfected.tif]

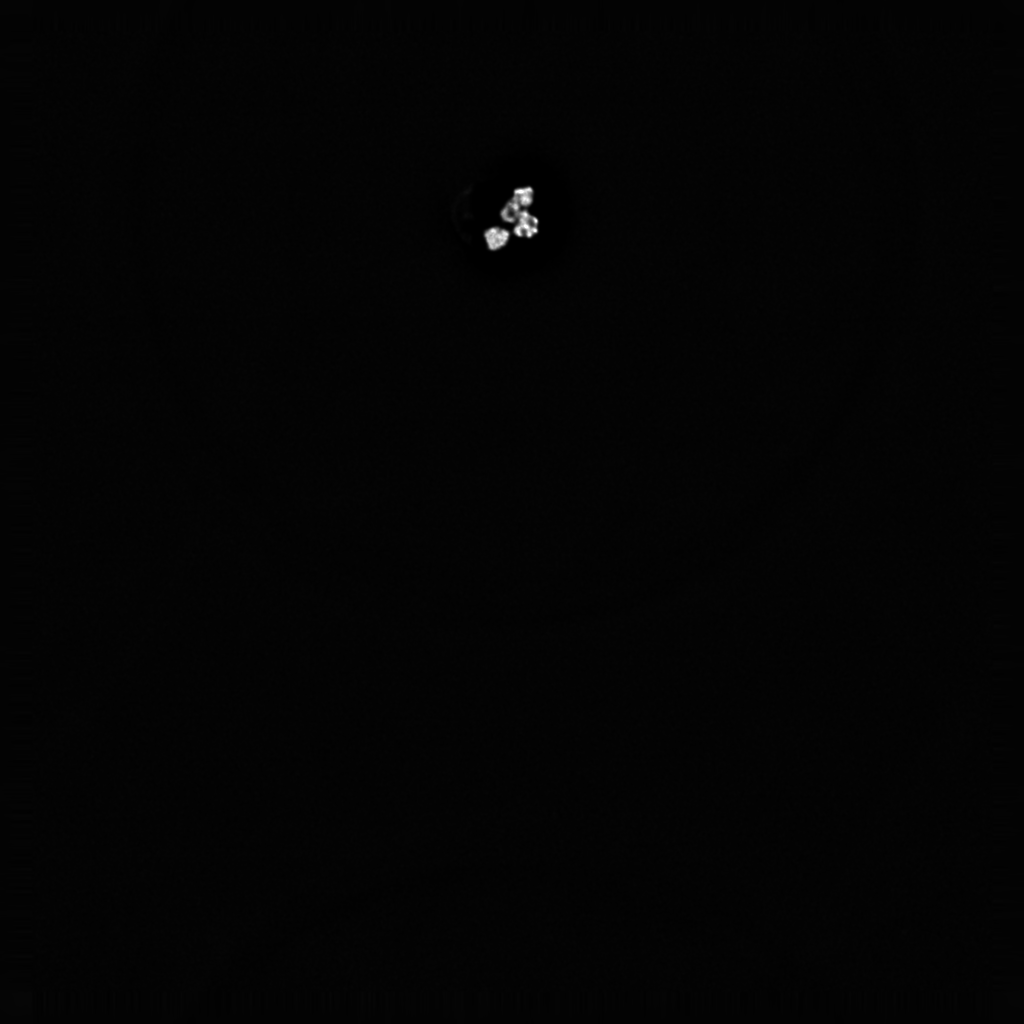

Supplement: Supplementary file 3 — Source data Fig. 1 [file 44321_2024_87_MOESM3_ESM.zip › Fig. 1/Figure 1C/Uncropped/Uninfected/1C_w435_Blue_Uninfected.tif]

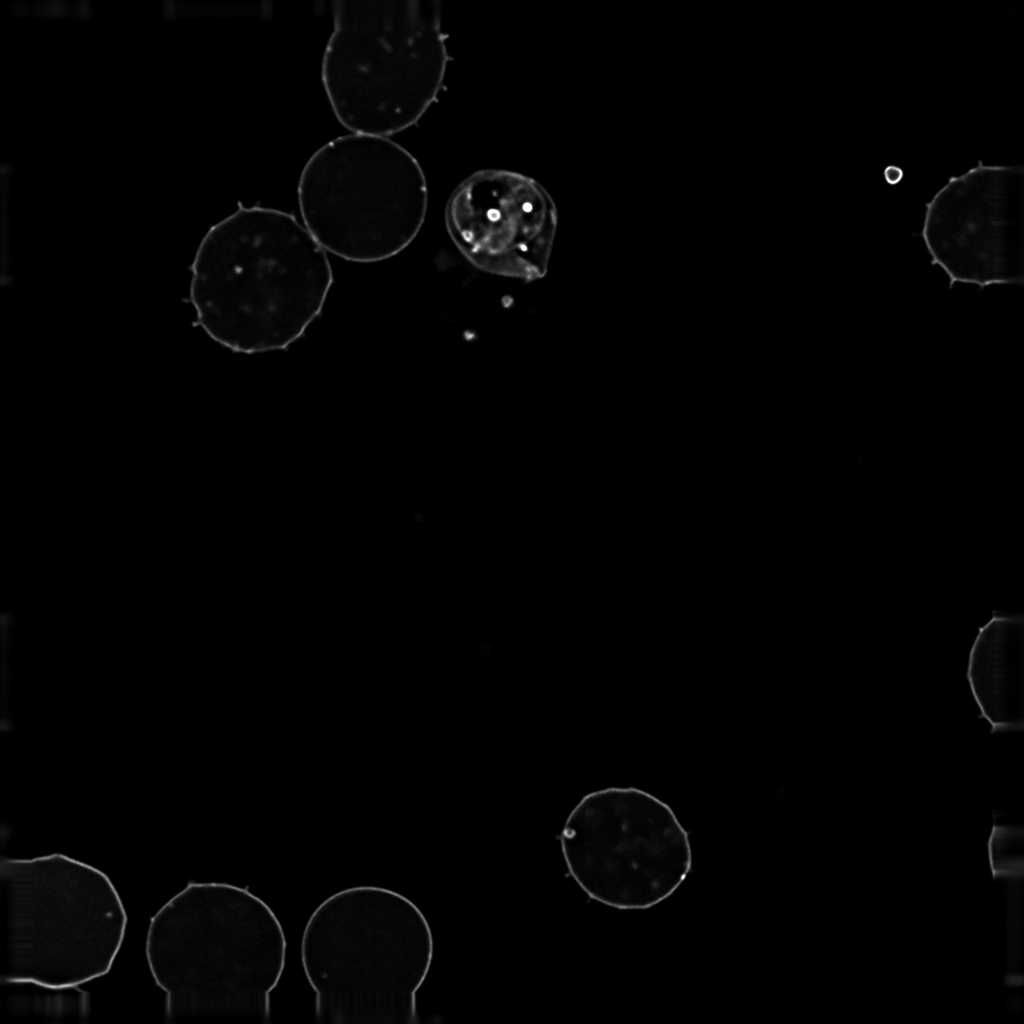

Supplement: Supplementary file 3 — Source data Fig. 1 [file 44321_2024_87_MOESM3_ESM.zip › Fig. 1/Figure 1C/Uncropped/Uninfected/1C_w523_Green_Uninfected.tif]

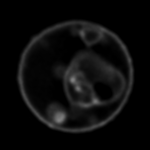

Supplement: Supplementary file 3 — Source data Fig. 1 [file 44321_2024_87_MOESM3_ESM.zip › Fig. 1/Figure 1C/Cropped/Infected/1C_w523_Green_Infected.tif]

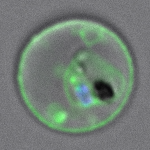

Supplement: Supplementary file 3 — Source data Fig. 1 [file 44321_2024_87_MOESM3_ESM.zip › Fig. 1/Figure 1C/Cropped/Infected/1C_Merge_Infected.tif]

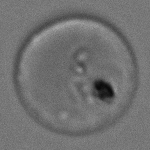

Supplement: Supplementary file 3 — Source data Fig. 1 [file 44321_2024_87_MOESM3_ESM.zip › Fig. 1/Figure 1C/Cropped/Infected/1C_DIC_Infected.tif]

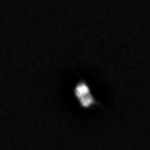

Supplement: Supplementary file 3 — Source data Fig. 1 [file 44321_2024_87_MOESM3_ESM.zip › Fig. 1/Figure 1C/Cropped/Infected/1C_w435_Blue_Infected.tif]

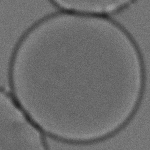

Supplement: Supplementary file 3 — Source data Fig. 1 [file 44321_2024_87_MOESM3_ESM.zip › Fig. 1/Figure 1C/Cropped/Uninfected/1C_DIC_Uninfected.tif]

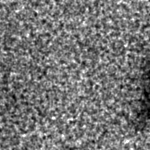

Supplement: Supplementary file 3 — Source data Fig. 1 [file 44321_2024_87_MOESM3_ESM.zip › Fig. 1/Figure 1C/Cropped/Uninfected/1C_w435_Blue_Uninfected.tif]

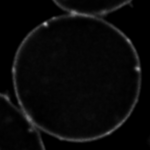

Supplement: Supplementary file 3 — Source data Fig. 1 [file 44321_2024_87_MOESM3_ESM.zip › Fig. 1/Figure 1C/Cropped/Uninfected/1C_w523_Green_Uninfected.tif]

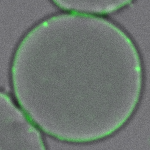

Supplement: Supplementary file 3 — Source data Fig. 1 [file 44321_2024_87_MOESM3_ESM.zip › Fig. 1/Figure 1C/Cropped/Uninfected/1C_Merge_Uninfected.tif]

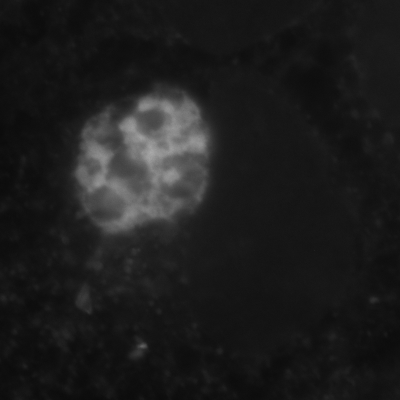

Supplement: Supplementary file 5 — Source data Fig. 3 [file 44321_2024_87_MOESM5_ESM.zip › Fig. 3/Figure 3B/Cropped Images/3B_Primaquine 3┬╡M/3B_Primaquine-3┬╡M_Green.tif]

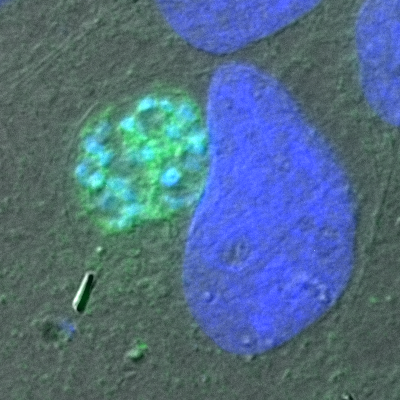

Supplement: Supplementary file 5 — Source data Fig. 3 [file 44321_2024_87_MOESM5_ESM.zip › Fig. 3/Figure 3B/Cropped Images/3B_Primaquine 3┬╡M/3B_Primaquine 3┬╡M_Merge.tif]

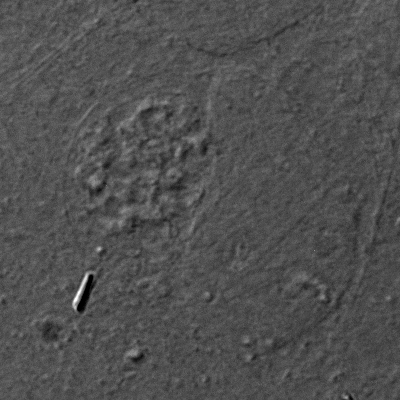

Supplement: Supplementary file 5 — Source data Fig. 3 [file 44321_2024_87_MOESM5_ESM.zip › Fig. 3/Figure 3B/Cropped Images/3B_Primaquine 3┬╡M/3B_Primaquine-3┬╡M_DIC.tif]

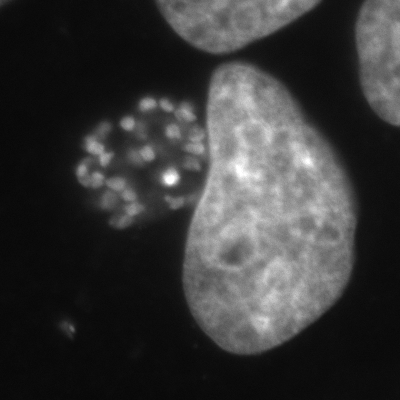

Supplement: Supplementary file 5 — Source data Fig. 3 [file 44321_2024_87_MOESM5_ESM.zip › Fig. 3/Figure 3B/Cropped Images/3B_Primaquine 3┬╡M/3B_Primaquine-3┬╡M_Blue.tif]

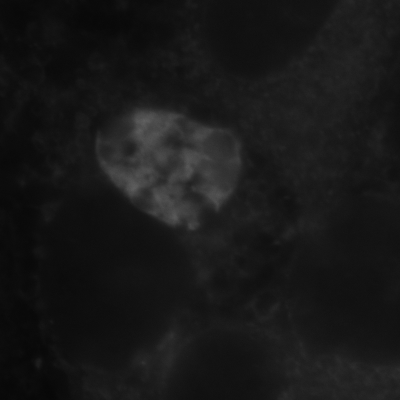

Supplement: Supplementary file 5 — Source data Fig. 3 [file 44321_2024_87_MOESM5_ESM.zip › Fig. 3/Figure 3B/Cropped Images/3B_C-17-PQ 0.3┬╡M/3B_C-17-PQ 0.3┬╡M_Green.tif]

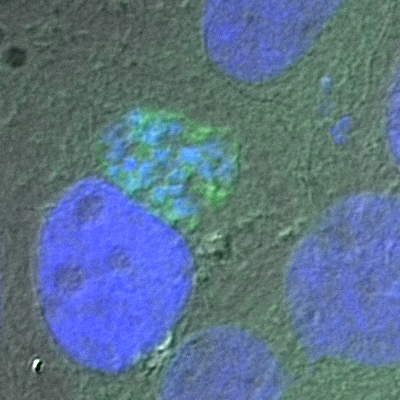

Supplement: Supplementary file 5 — Source data Fig. 3 [file 44321_2024_87_MOESM5_ESM.zip › Fig. 3/Figure 3B/Cropped Images/3B_C-17-PQ 0.3┬╡M/3B_C-17-PQ 0.3┬╡M_Merge.tif]

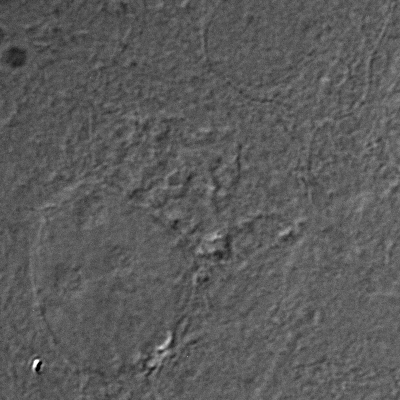

Supplement: Supplementary file 5 — Source data Fig. 3 [file 44321_2024_87_MOESM5_ESM.zip › Fig. 3/Figure 3B/Cropped Images/3B_C-17-PQ 0.3┬╡M/3B_C-17-PQ 0.3┬╡M_DIC.tif]

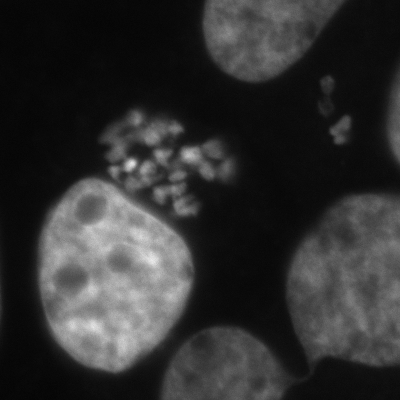

Supplement: Supplementary file 5 — Source data Fig. 3 [file 44321_2024_87_MOESM5_ESM.zip › Fig. 3/Figure 3B/Cropped Images/3B_C-17-PQ 0.3┬╡M/3B_C-17-PQ 0.3┬╡M_Blue.tif]

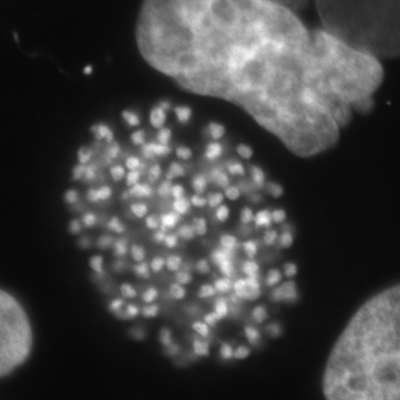

Supplement: Supplementary file 5 — Source data Fig. 3 [file 44321_2024_87_MOESM5_ESM.zip › Fig. 3/Figure 3B/Cropped Images/3B_DMSO/3B_DMSO_Blue.tif]

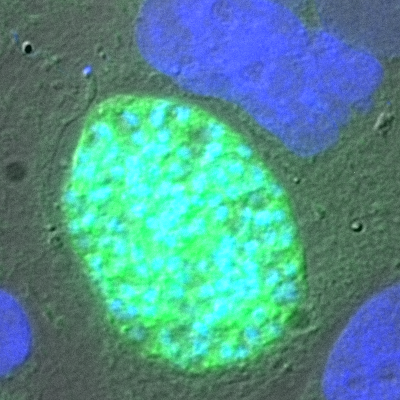

Supplement: Supplementary file 5 — Source data Fig. 3 [file 44321_2024_87_MOESM5_ESM.zip › Fig. 3/Figure 3B/Cropped Images/3B_DMSO/3B_DMSO_Merge.tif]

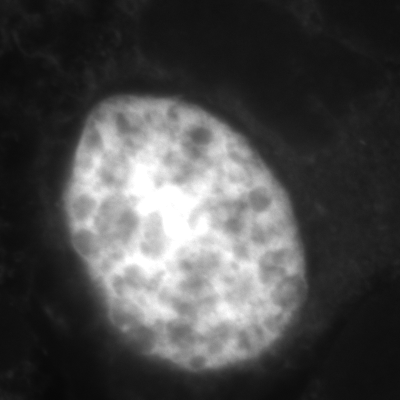

Supplement: Supplementary file 5 — Source data Fig. 3 [file 44321_2024_87_MOESM5_ESM.zip › Fig. 3/Figure 3B/Cropped Images/3B_DMSO/3B_DMSO_Green.tif]

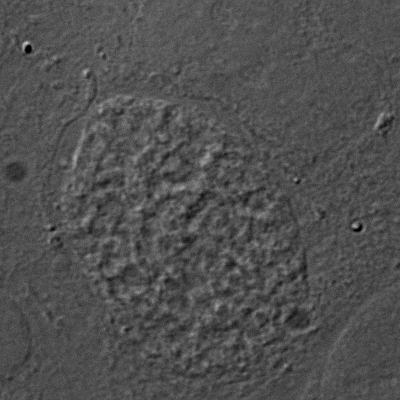

Supplement: Supplementary file 5 — Source data Fig. 3 [file 44321_2024_87_MOESM5_ESM.zip › Fig. 3/Figure 3B/Cropped Images/3B_DMSO/3B_DMSO_DIC.tif]

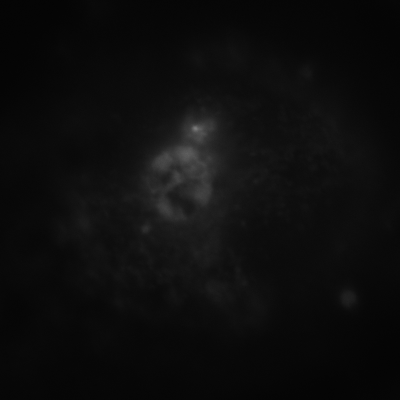

Supplement: Supplementary file 5 — Source data Fig. 3 [file 44321_2024_87_MOESM5_ESM.zip › Fig. 3/Figure 3B/Cropped Images/3B_C-17-PQ 3┬╡M/3B_C-17-PQ 3┬╡M_Green.tf.tiff]

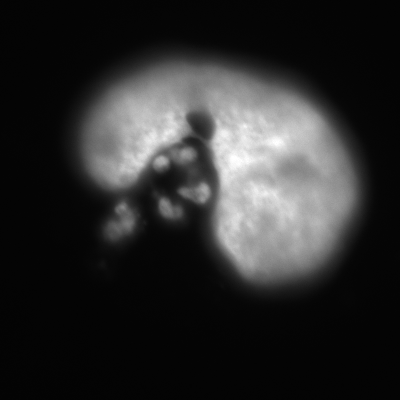

Supplement: Supplementary file 5 — Source data Fig. 3 [file 44321_2024_87_MOESM5_ESM.zip › Fig. 3/Figure 3B/Cropped Images/3B_C-17-PQ 3┬╡M/3B_C-17-PQ 3┬╡M_Blue.tif]

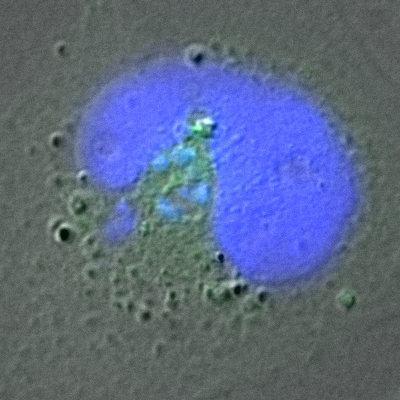

Supplement: Supplementary file 5 — Source data Fig. 3 [file 44321_2024_87_MOESM5_ESM.zip › Fig. 3/Figure 3B/Cropped Images/3B_C-17-PQ 3┬╡M/3B_C-17-PQ 3┬╡M_Merge.tif]

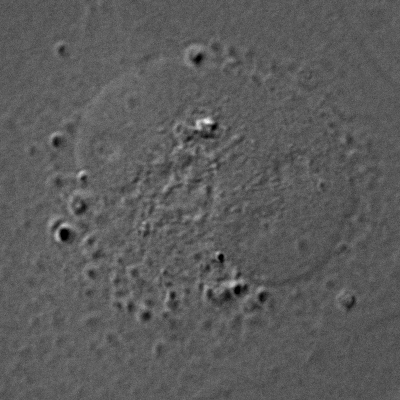

Supplement: Supplementary file 5 — Source data Fig. 3 [file 44321_2024_87_MOESM5_ESM.zip › Fig. 3/Figure 3B/Cropped Images/3B_C-17-PQ 3┬╡M/3B_C-17-PQ 3┬╡M_DIC.tif]

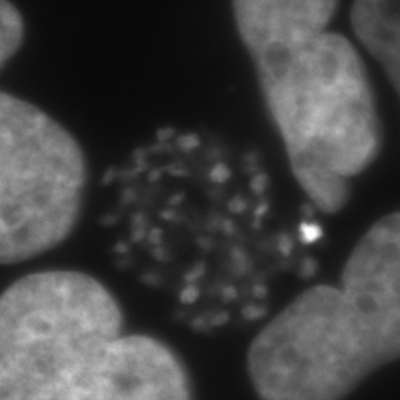

Supplement: Supplementary file 5 — Source data Fig. 3 [file 44321_2024_87_MOESM5_ESM.zip › Fig. 3/Figure 3B/Cropped Images/3B_Primaquine 0.3┬╡M/3B_Primaquine 0.3┬╡M_Blue.tif]

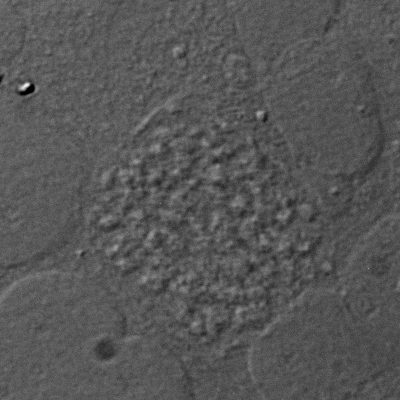

Supplement: Supplementary file 5 — Source data Fig. 3 [file 44321_2024_87_MOESM5_ESM.zip › Fig. 3/Figure 3B/Cropped Images/3B_Primaquine 0.3┬╡M/3B_Primaquine 0.3┬╡M_DIC.tif]

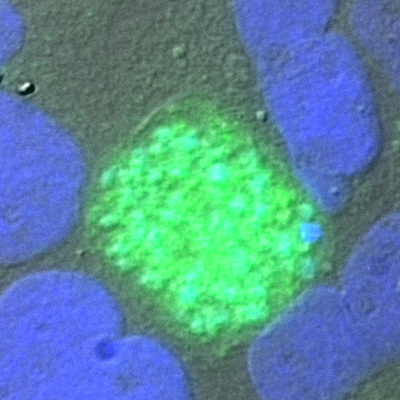

Supplement: Supplementary file 5 — Source data Fig. 3 [file 44321_2024_87_MOESM5_ESM.zip › Fig. 3/Figure 3B/Cropped Images/3B_Primaquine 0.3┬╡M/3B_Primaquine 0.3┬╡M_Merge.tif]

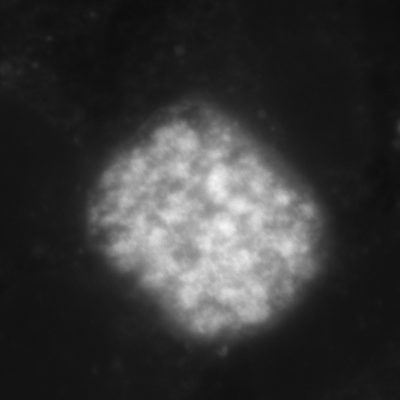

Supplement: Supplementary file 5 — Source data Fig. 3 [file 44321_2024_87_MOESM5_ESM.zip › Fig. 3/Figure 3B/Cropped Images/3B_Primaquine 0.3┬╡M/3B_Primaquine-0.3┬╡M_Green.tif]

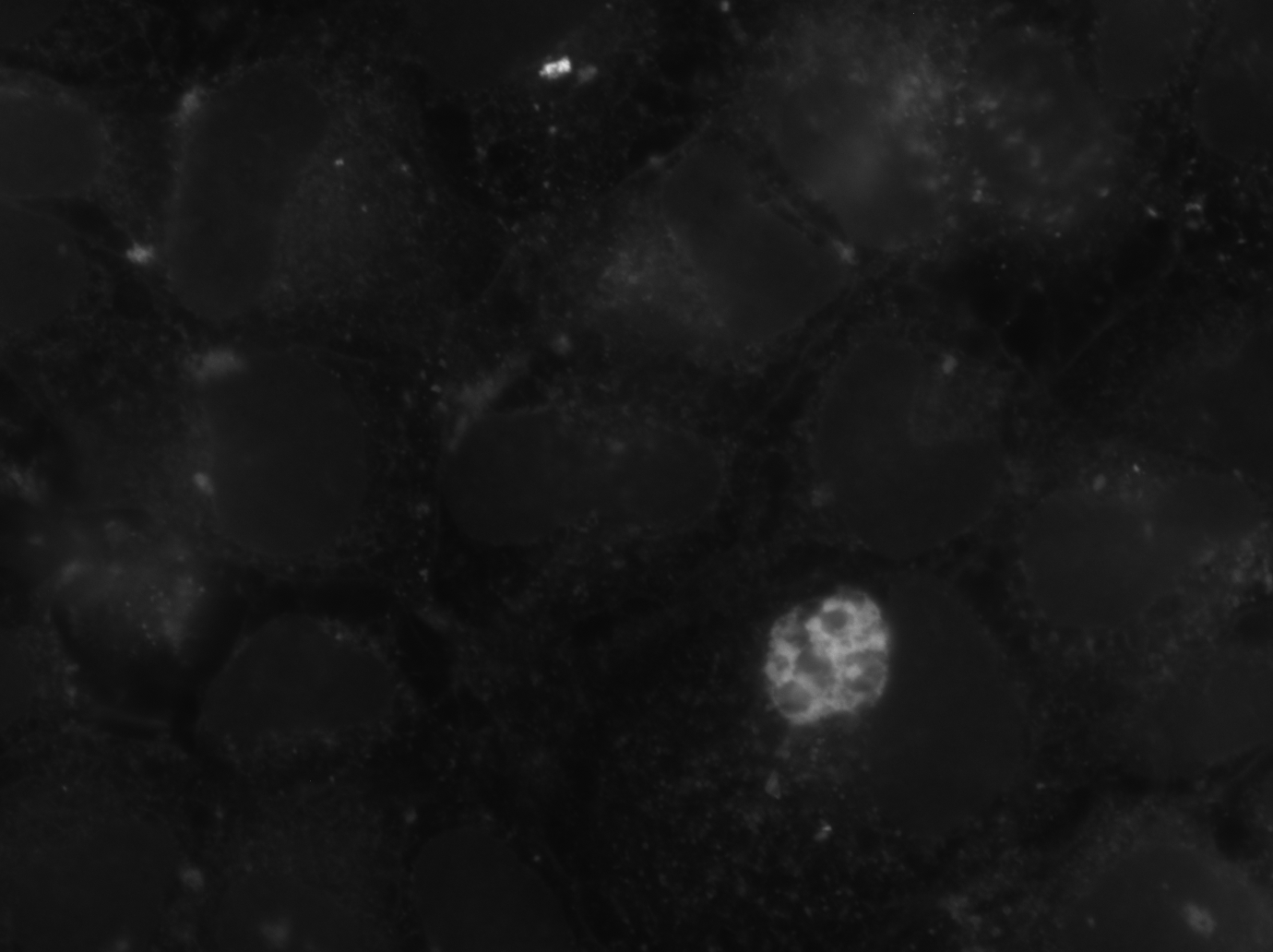

Supplement: Supplementary file 5 — Source data Fig. 3 [file 44321_2024_87_MOESM5_ESM.zip › Fig. 3/Figure 3B/Uncropped/3B_Primaquine 3┬╡M/3B_Primaquine 3┬╡M_Green.tif]

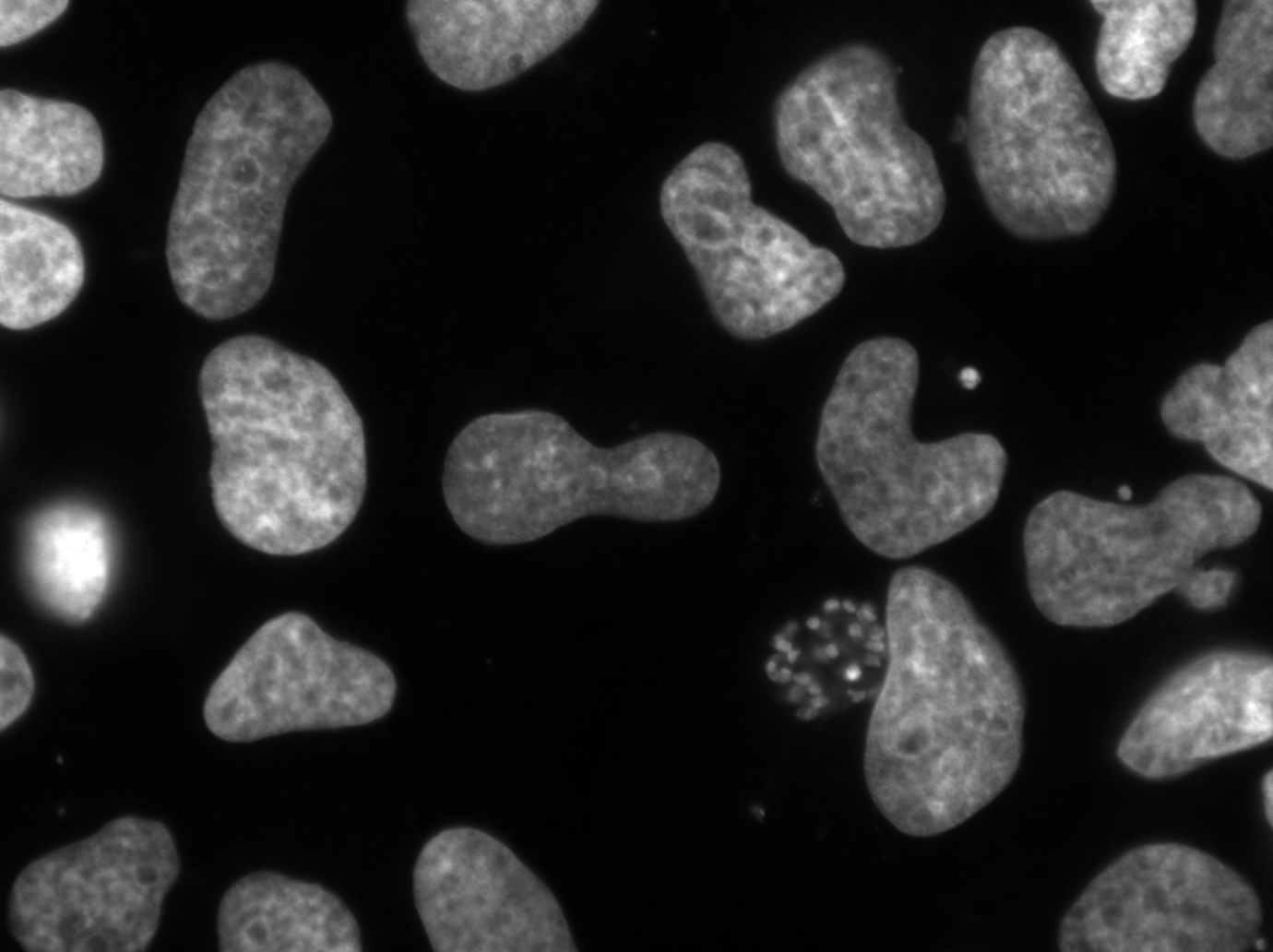

Supplement: Supplementary file 5 — Source data Fig. 3 [file 44321_2024_87_MOESM5_ESM.zip › Fig. 3/Figure 3B/Uncropped/3B_Primaquine 3┬╡M/3B_Primaquine 3┬╡M_Blue.tif]

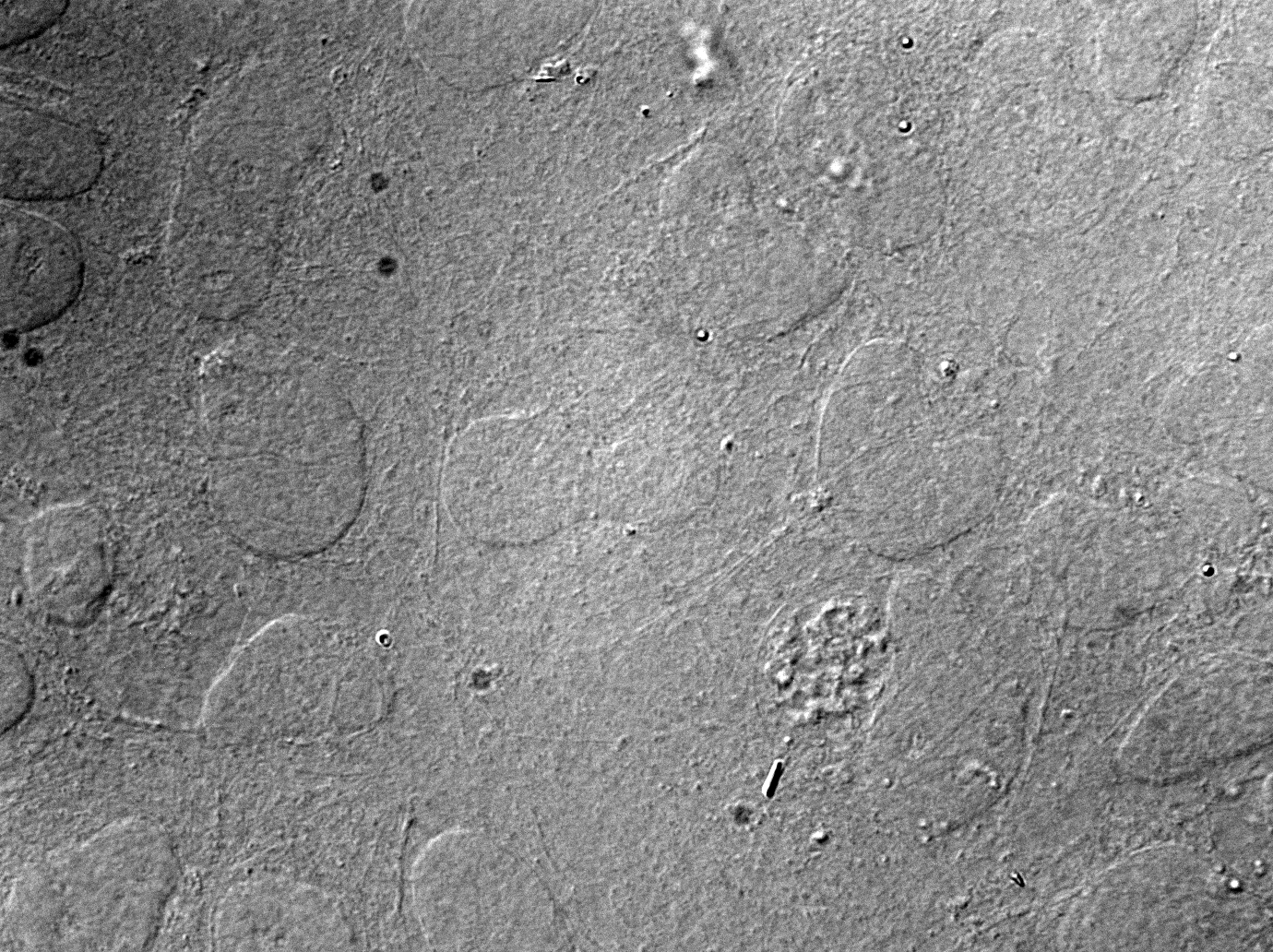

Supplement: Supplementary file 5 — Source data Fig. 3 [file 44321_2024_87_MOESM5_ESM.zip › Fig. 3/Figure 3B/Uncropped/3B_Primaquine 3┬╡M/3B_Primaquine 3┬╡M_DIC.tif]

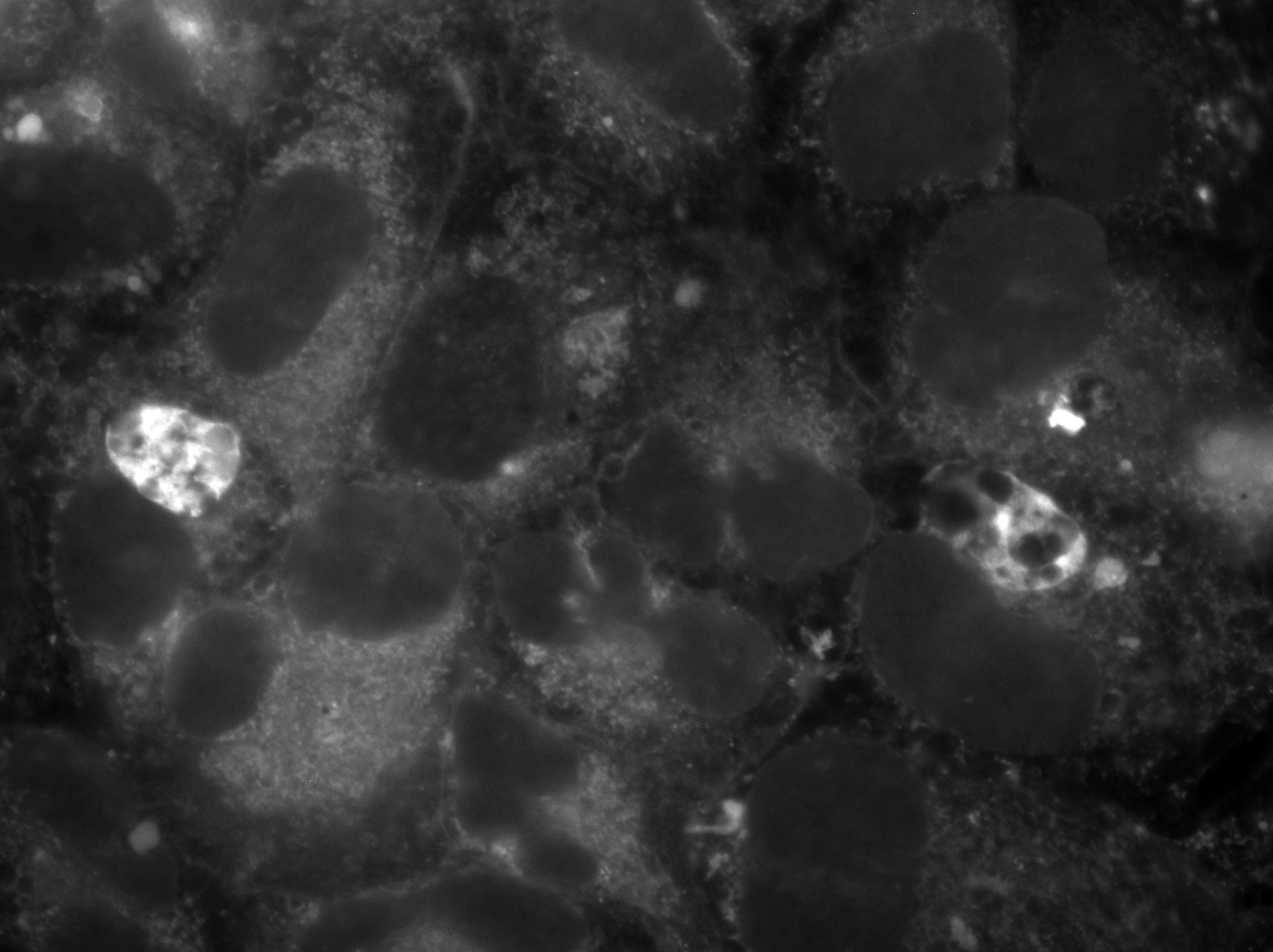

Supplement: Supplementary file 5 — Source data Fig. 3 [file 44321_2024_87_MOESM5_ESM.zip › Fig. 3/Figure 3B/Uncropped/3B_C-17-PQ 0.3┬╡M/3B_C-17-PQ 0.3┬╡M_Green.tif]

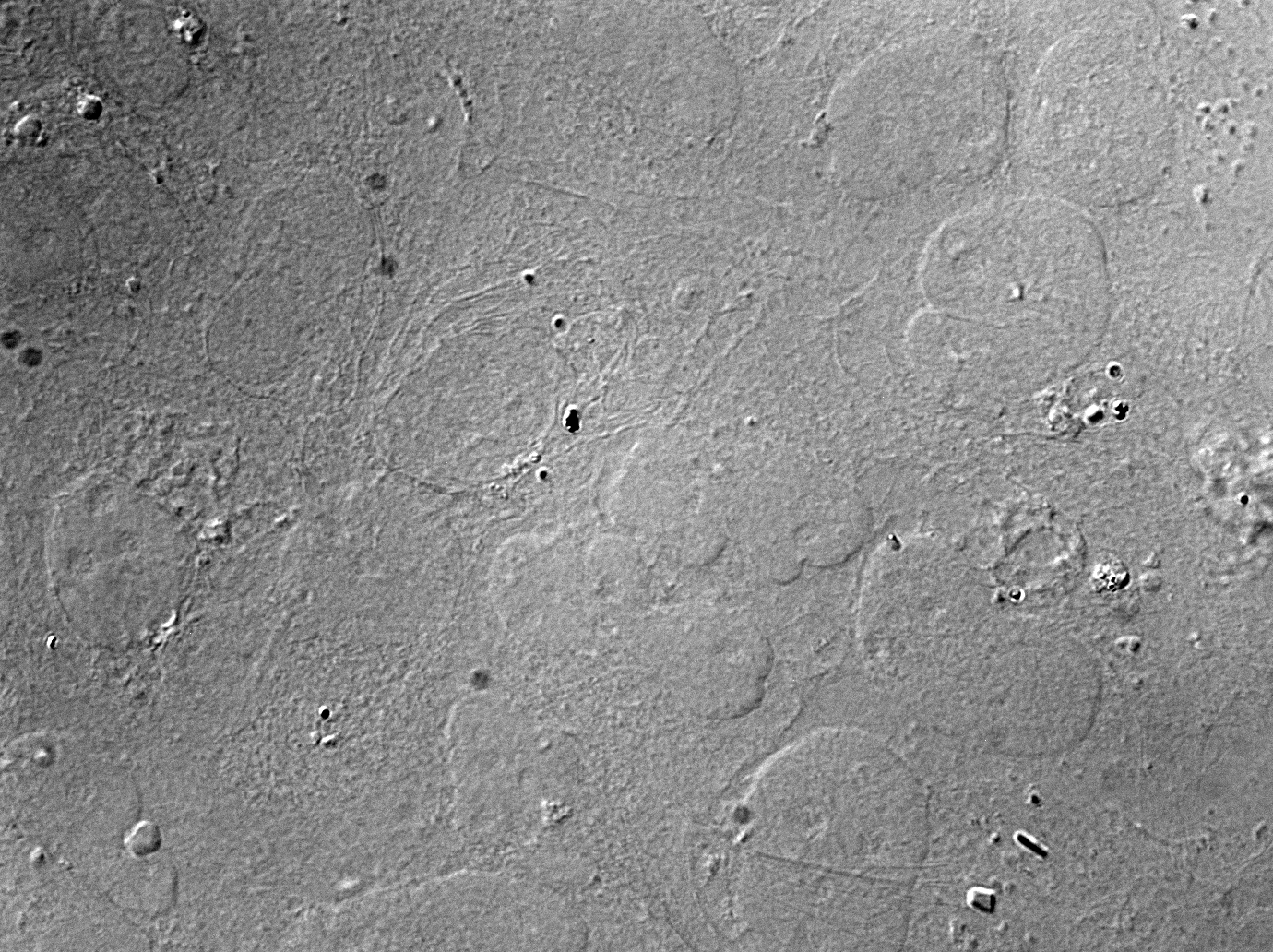

Supplement: Supplementary file 5 — Source data Fig. 3 [file 44321_2024_87_MOESM5_ESM.zip › Fig. 3/Figure 3B/Uncropped/3B_C-17-PQ 0.3┬╡M/3B_C-17-PQ 0.3┬╡M_DIC.tif]

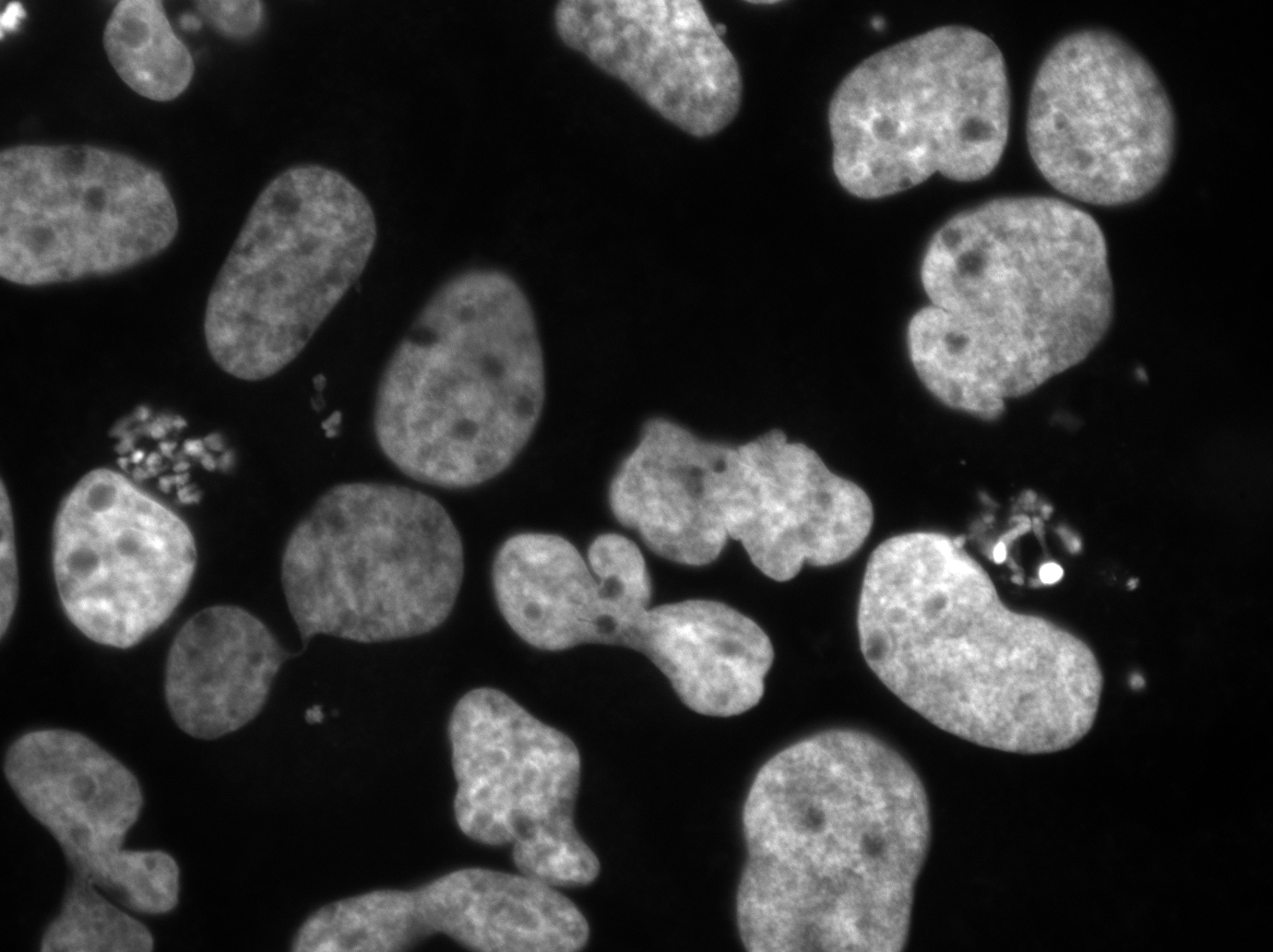

Supplement: Supplementary file 5 — Source data Fig. 3 [file 44321_2024_87_MOESM5_ESM.zip › Fig. 3/Figure 3B/Uncropped/3B_C-17-PQ 0.3┬╡M/3B_C-17-PQ 0.3┬╡M_Blue.tif]

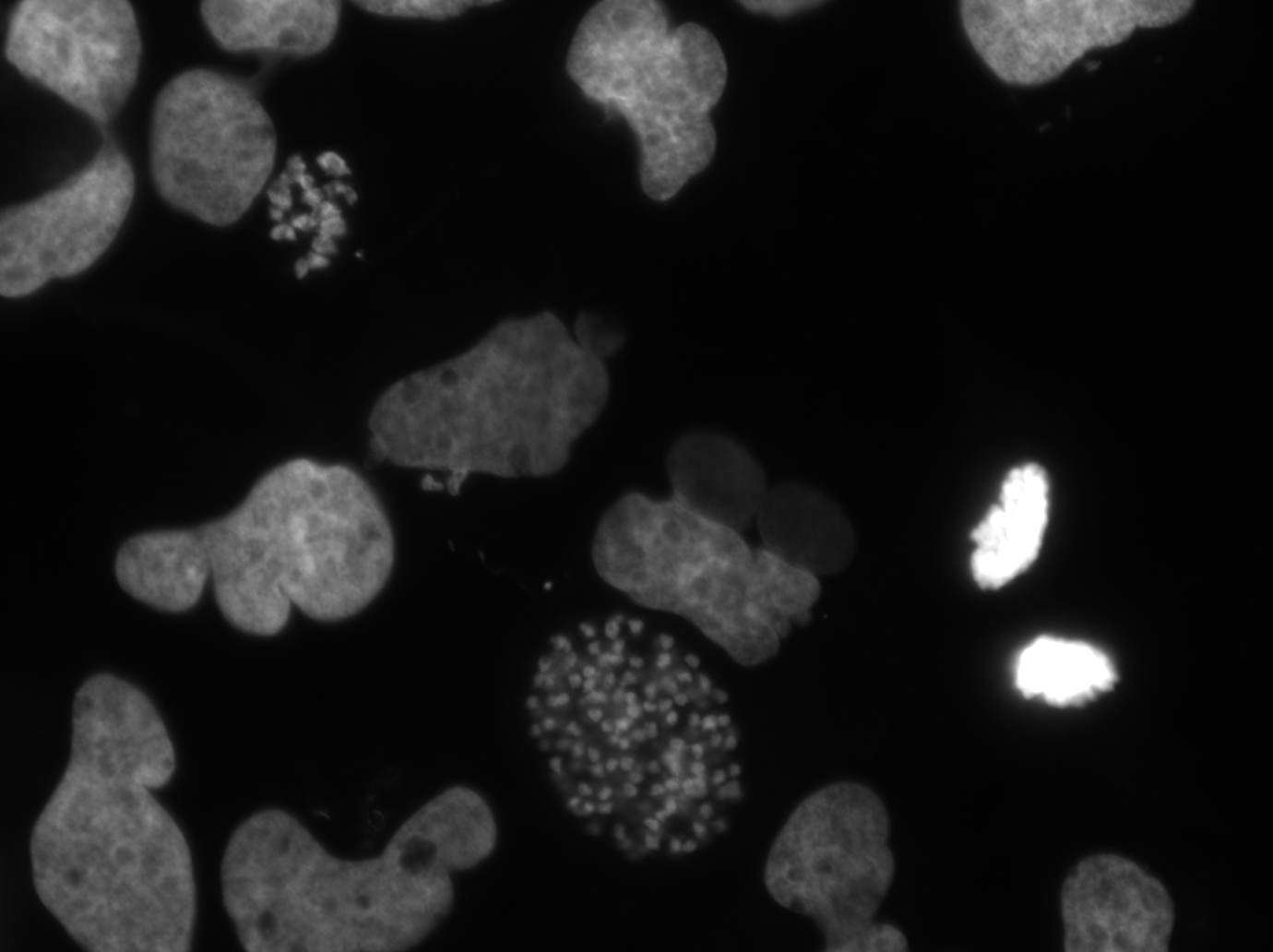

Supplement: Supplementary file 5 — Source data Fig. 3 [file 44321_2024_87_MOESM5_ESM.zip › Fig. 3/Figure 3B/Uncropped/3B_DMSO/3B_DMSO_Blue.tif]

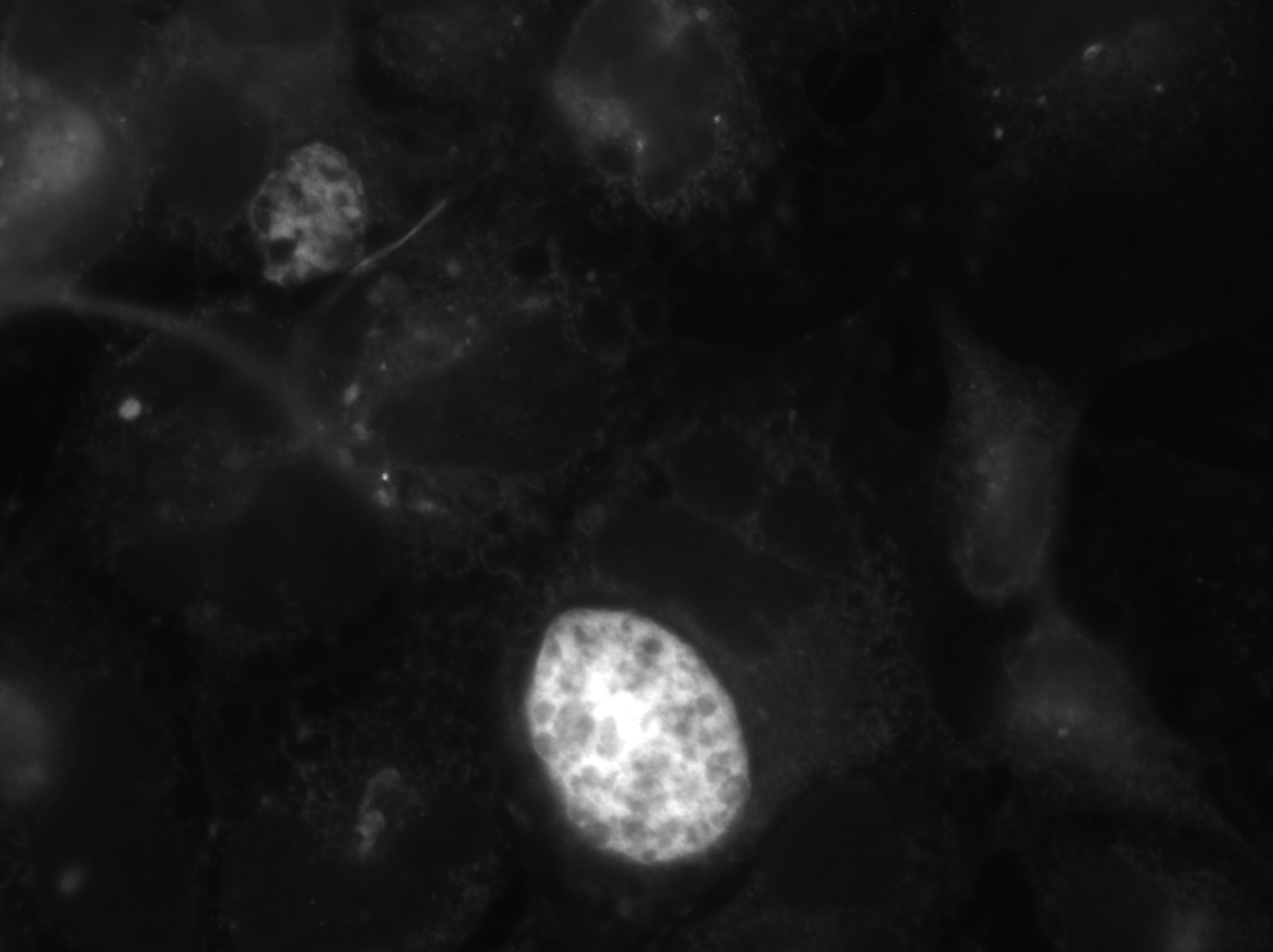

Supplement: Supplementary file 5 — Source data Fig. 3 [file 44321_2024_87_MOESM5_ESM.zip › Fig. 3/Figure 3B/Uncropped/3B_DMSO/3B_DMSO_Green.tif]

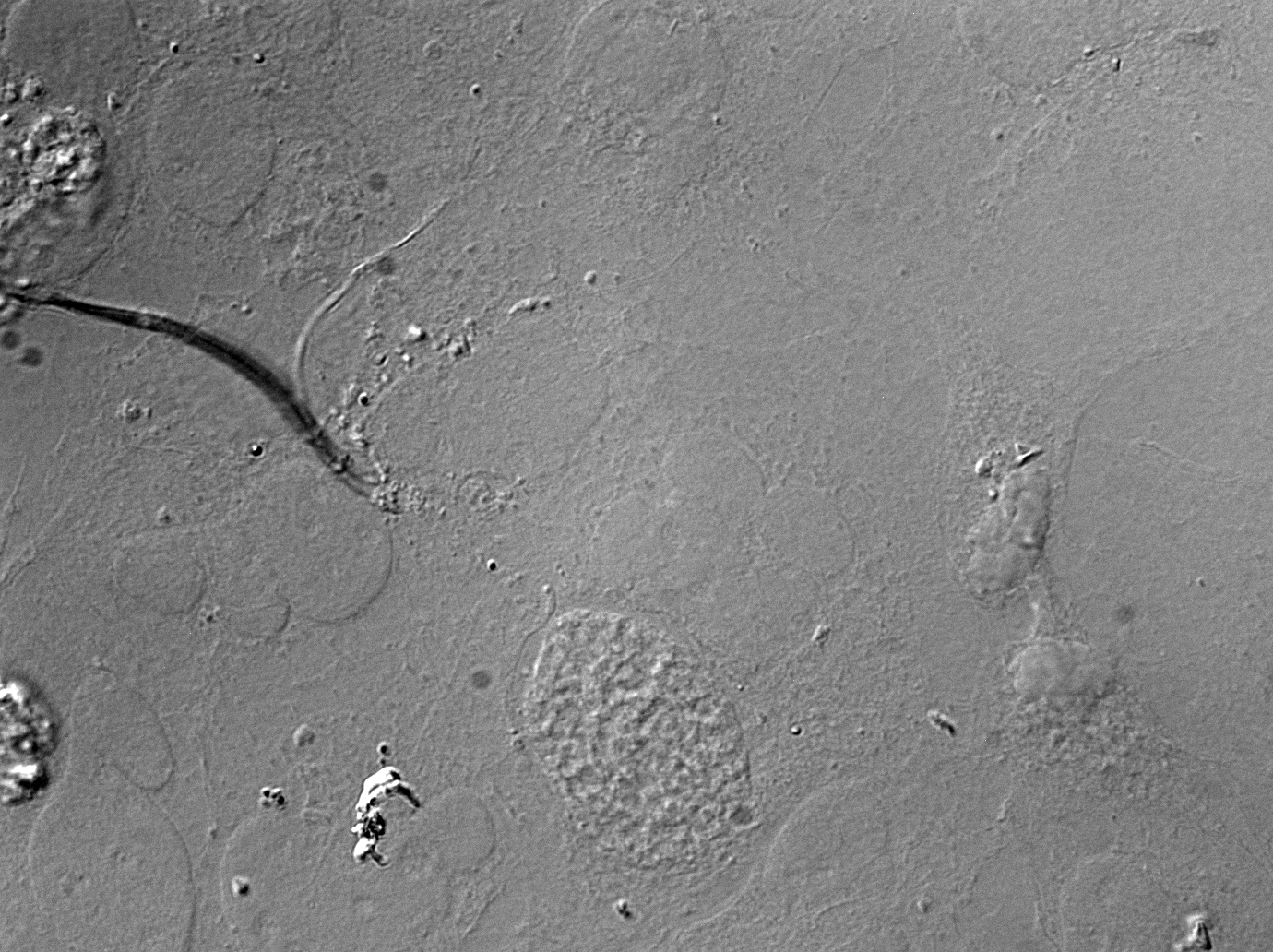

Supplement: Supplementary file 5 — Source data Fig. 3 [file 44321_2024_87_MOESM5_ESM.zip › Fig. 3/Figure 3B/Uncropped/3B_DMSO/3B_DMSO_DIC.tif]
