# Supplementary figures and images for: Harnessing cholesterol uptake of malaria parasites for therapeutic applications (part 2 of 2)
Source: EMBO Mol Med. 2024 Jun 11;16(7):4. doi: 10.1038/s44321-024-00087-1 (PMC11251039; doi:10.1038/s44321-024-00087-1)

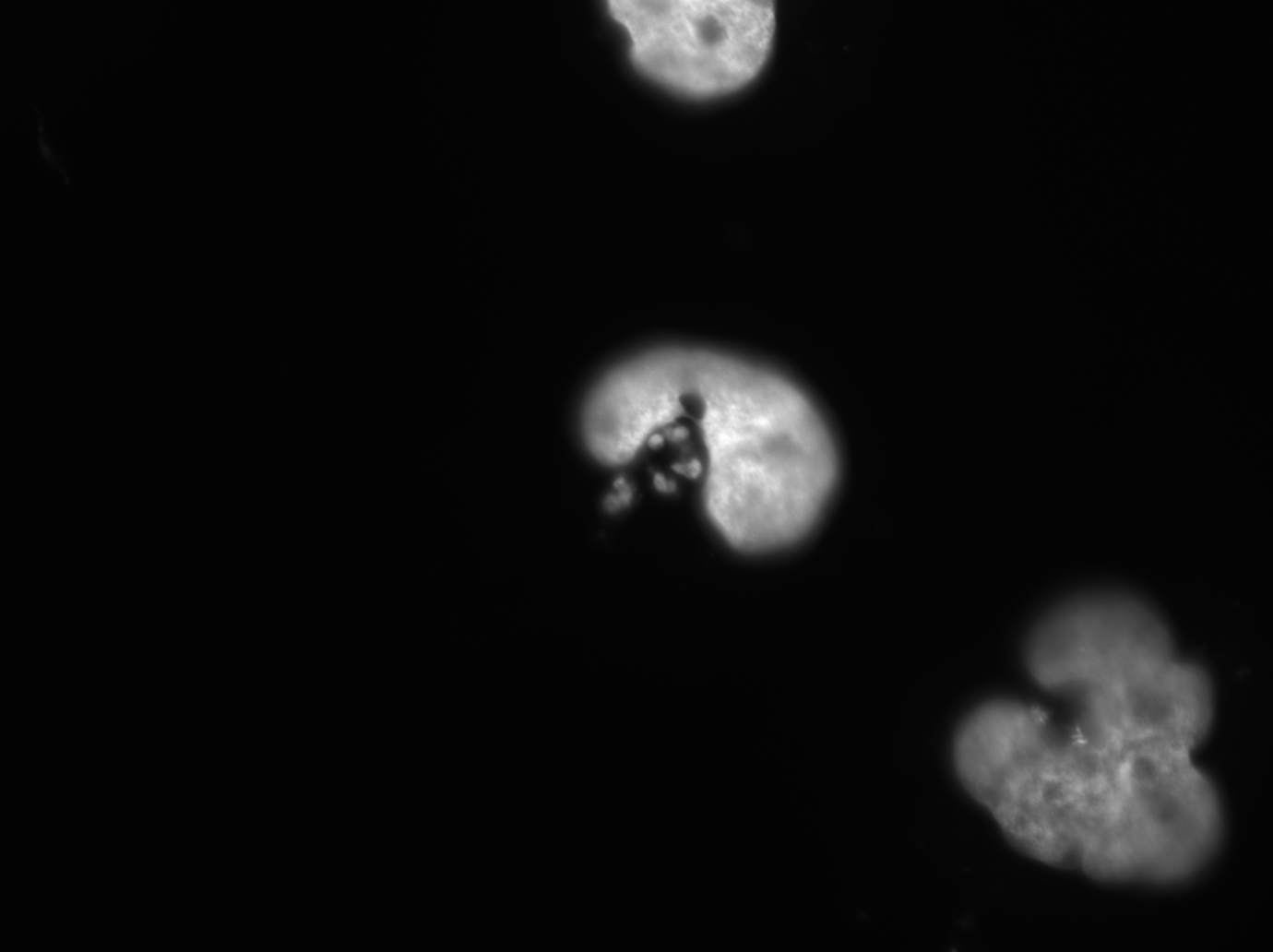

Supplement: Supplementary file 5 — Source data Fig. 3 [file 44321_2024_87_MOESM5_ESM.zip › Fig. 3/Figure 3B/Uncropped/3B_C-17-PQ 3┬╡M/3B_C-17-PQ 3┬╡M_Blue.tif]

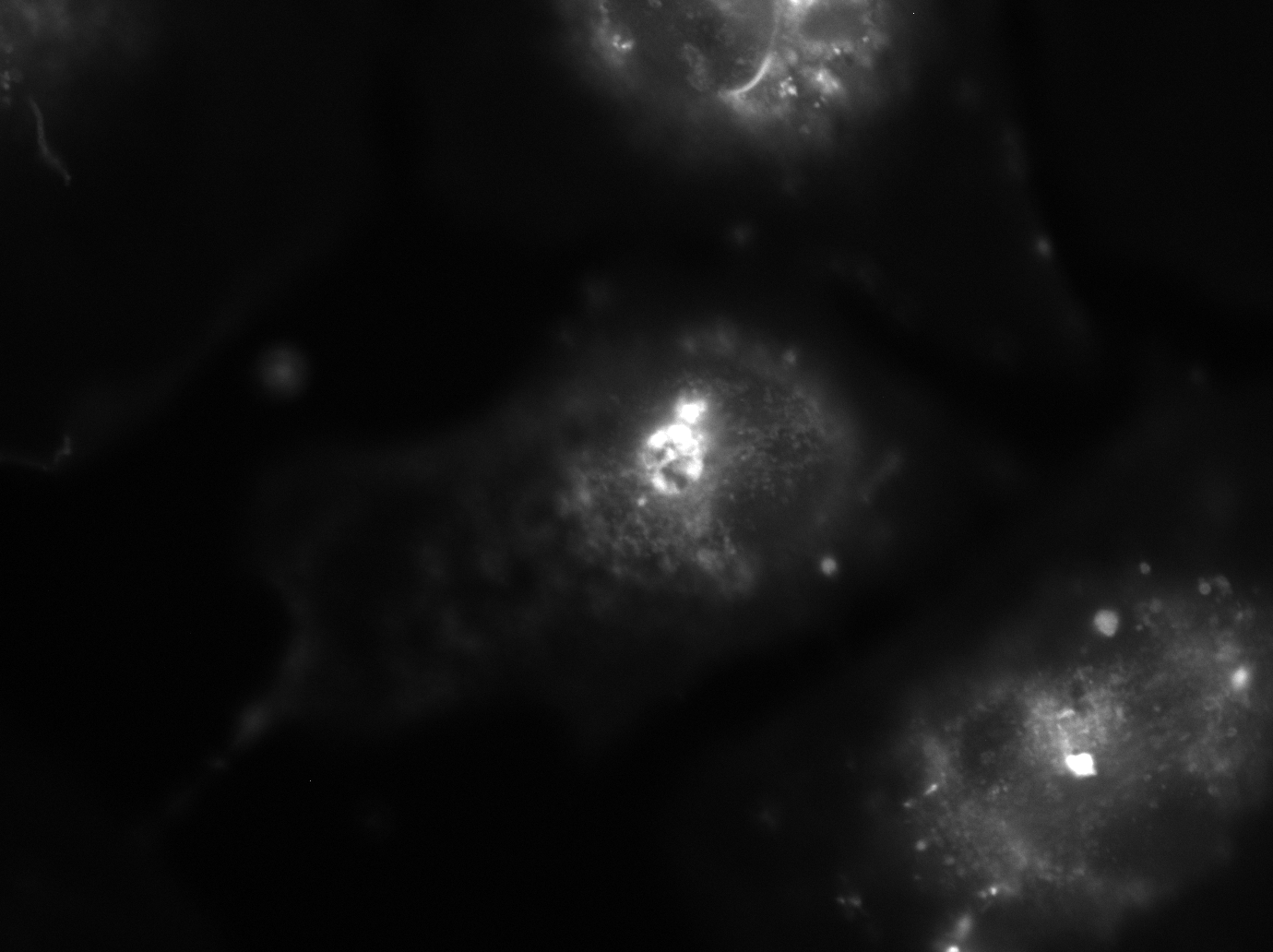

Supplement: Supplementary file 5 — Source data Fig. 3 [file 44321_2024_87_MOESM5_ESM.zip › Fig. 3/Figure 3B/Uncropped/3B_C-17-PQ 3┬╡M/3B_C-17-PQ 3┬╡M_Green.tif]

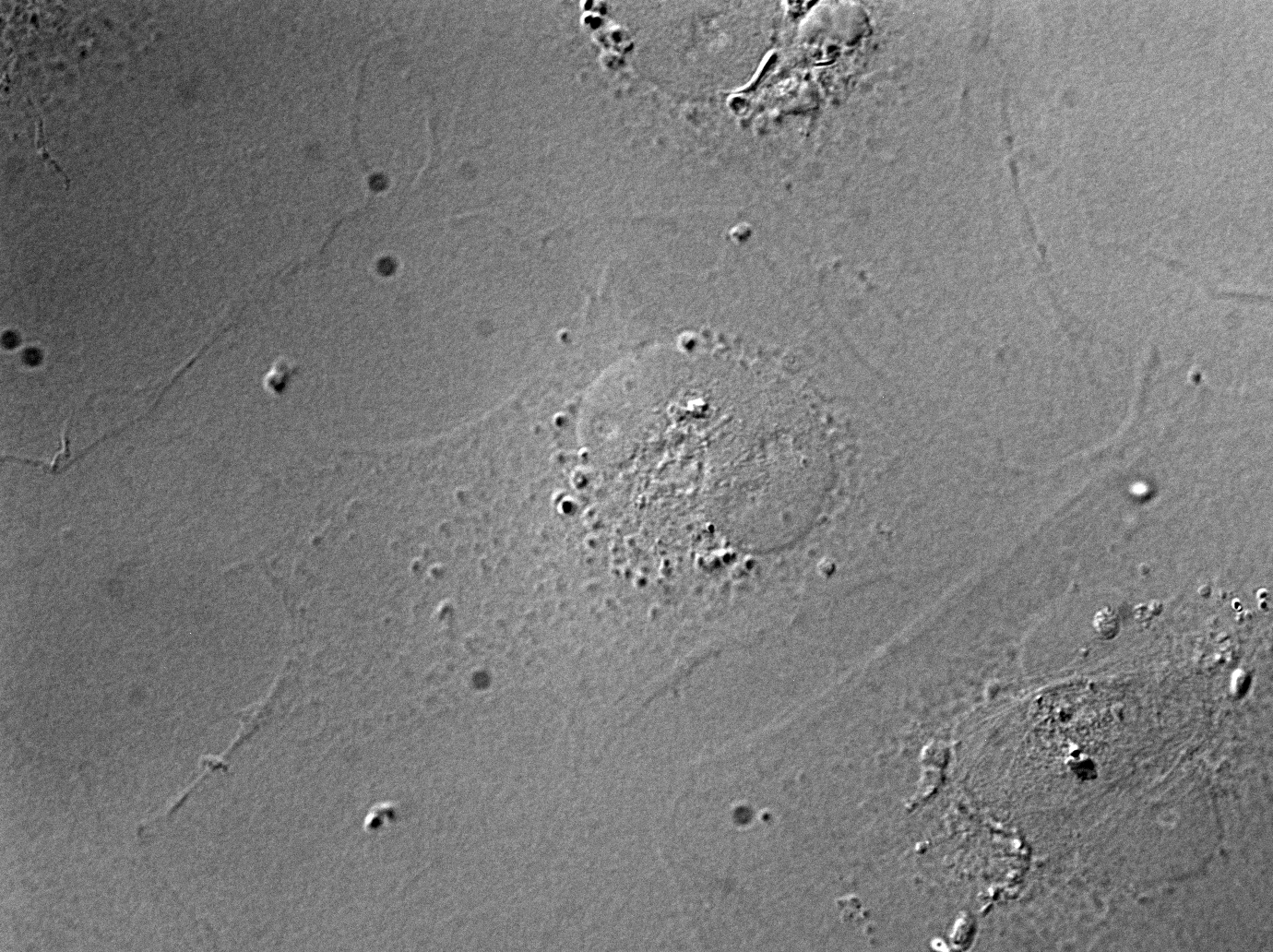

Supplement: Supplementary file 5 — Source data Fig. 3 [file 44321_2024_87_MOESM5_ESM.zip › Fig. 3/Figure 3B/Uncropped/3B_C-17-PQ 3┬╡M/3B_C-17-PQ 3┬╡M_DIC.tif]

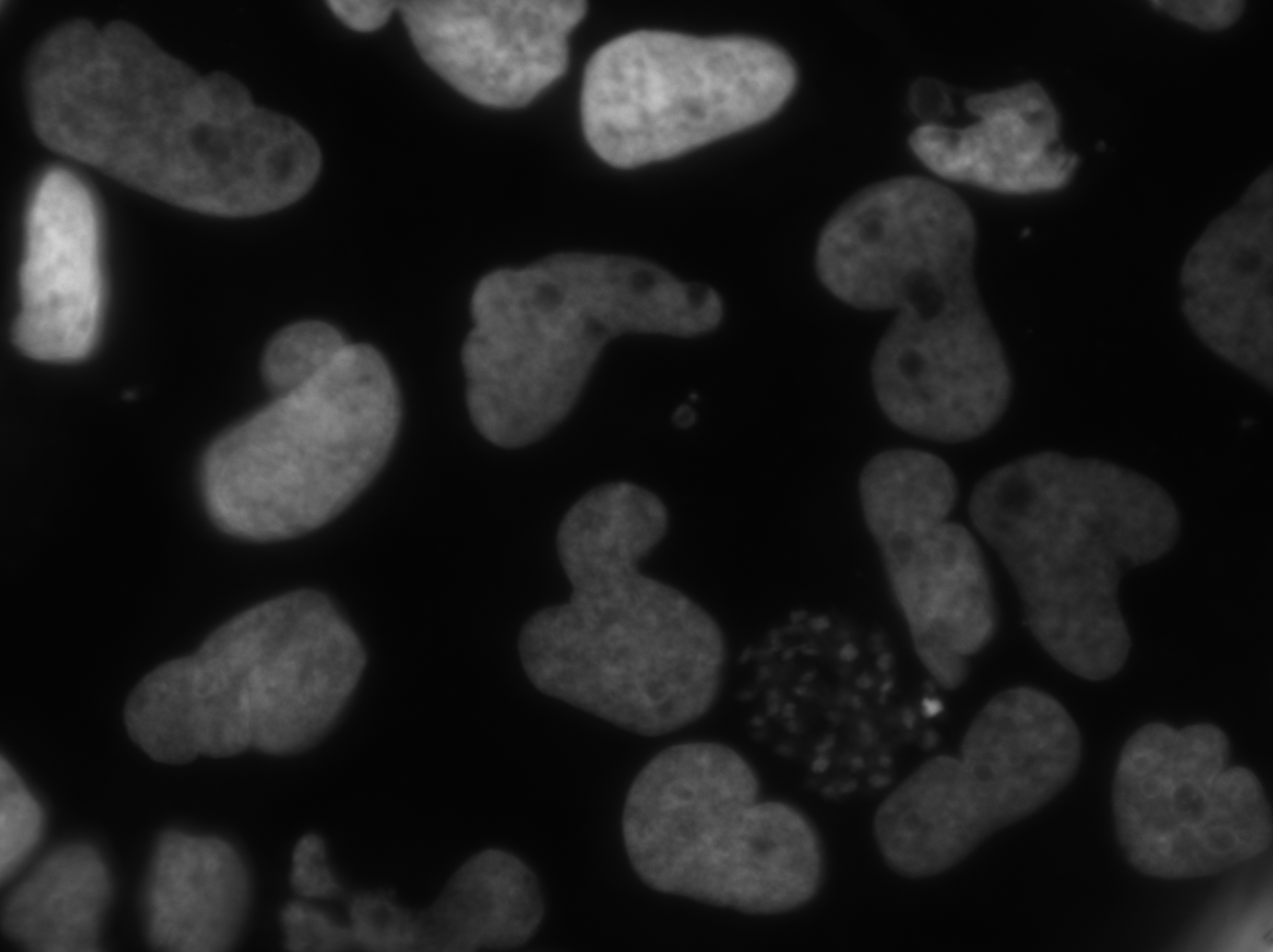

Supplement: Supplementary file 5 — Source data Fig. 3 [file 44321_2024_87_MOESM5_ESM.zip › Fig. 3/Figure 3B/Uncropped/3B_Primaquine 0.3┬╡M/3B_Primaquine 0.3┬╡M_Blue.tif]

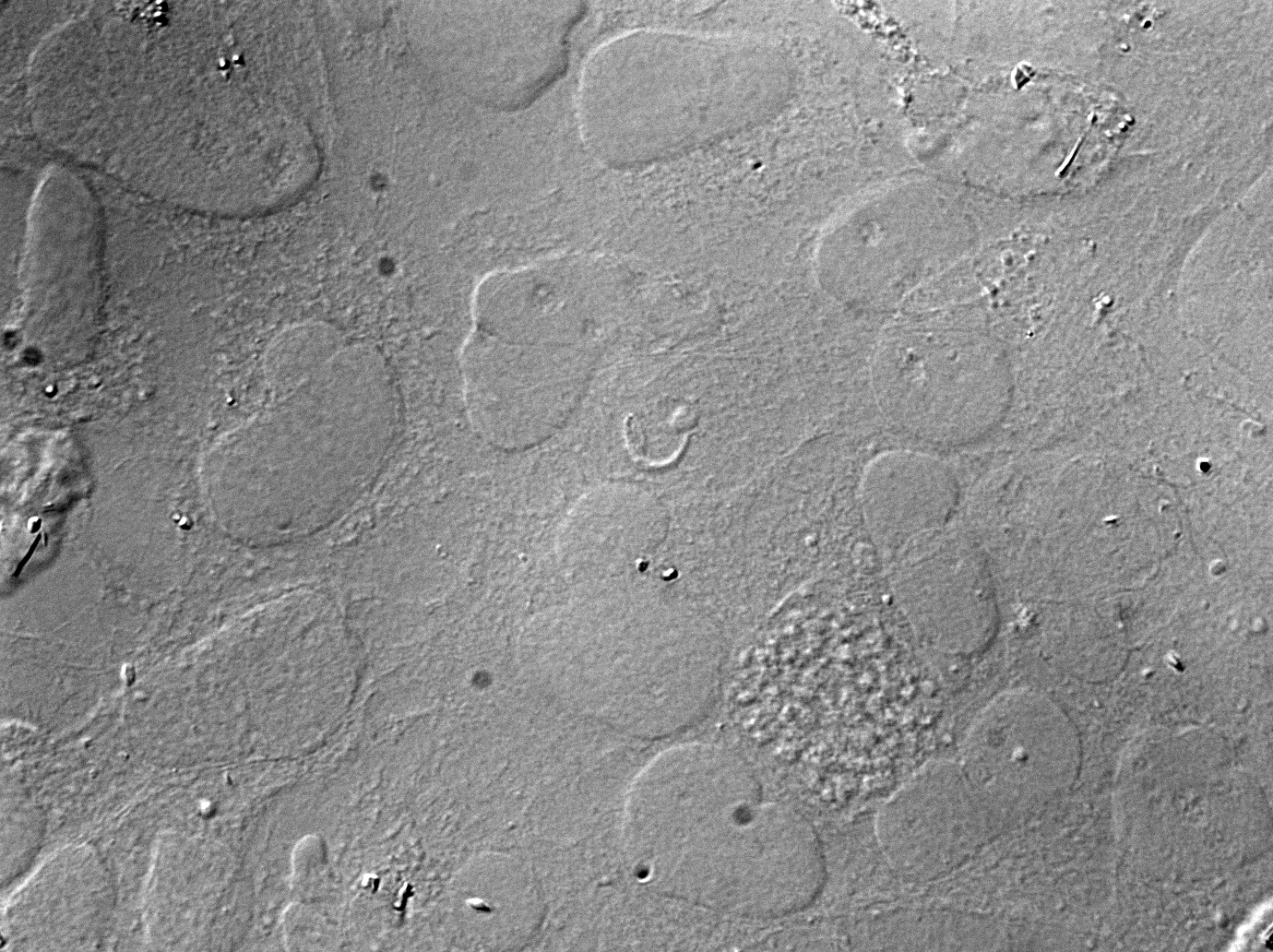

Supplement: Supplementary file 5 — Source data Fig. 3 [file 44321_2024_87_MOESM5_ESM.zip › Fig. 3/Figure 3B/Uncropped/3B_Primaquine 0.3┬╡M/3B_Primaquine 0.3┬╡M_DIC.tif]

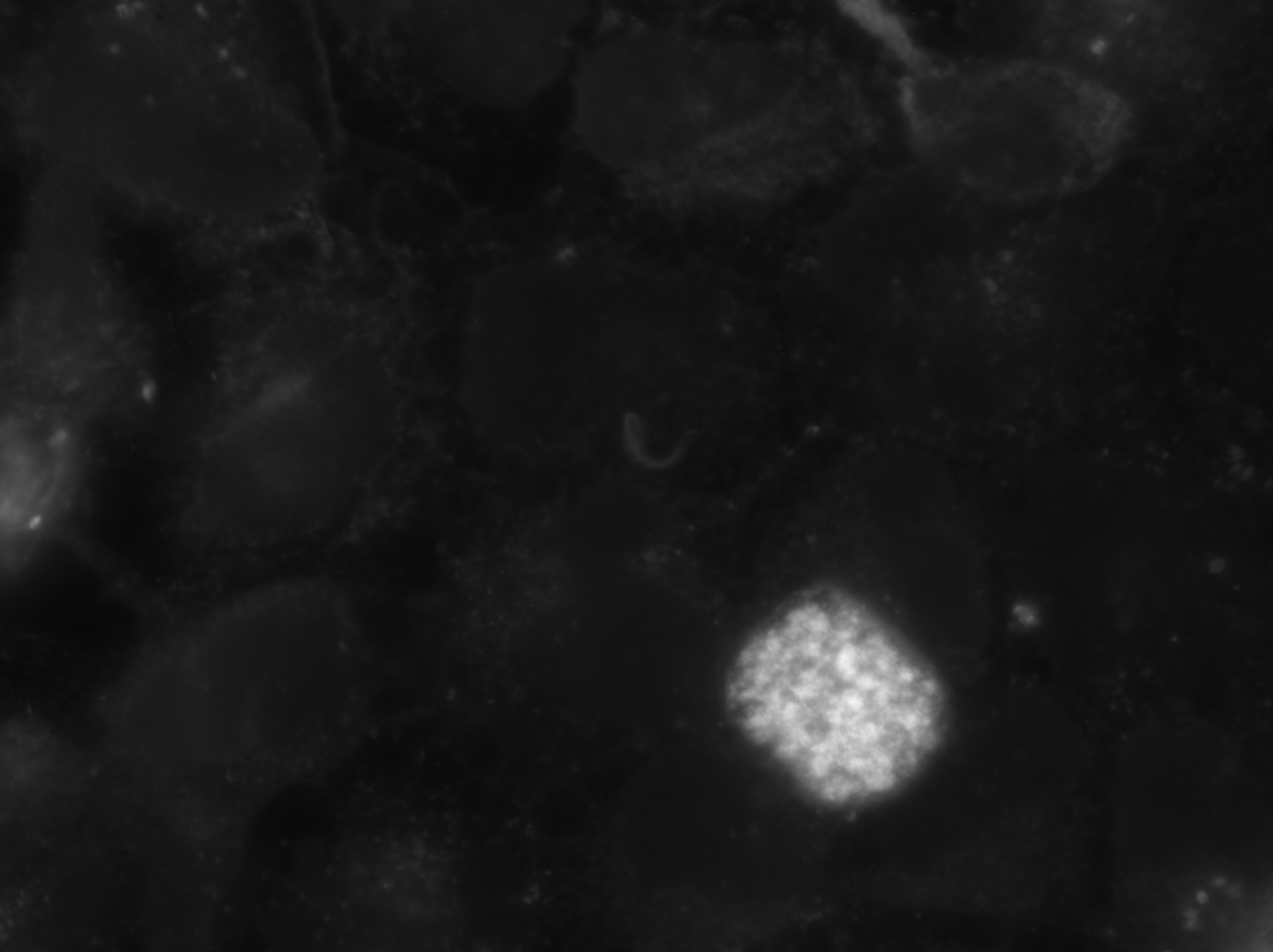

Supplement: Supplementary file 5 — Source data Fig. 3 [file 44321_2024_87_MOESM5_ESM.zip › Fig. 3/Figure 3B/Uncropped/3B_Primaquine 0.3┬╡M/3B_Primaquine 0.3┬╡M_Green.tif]

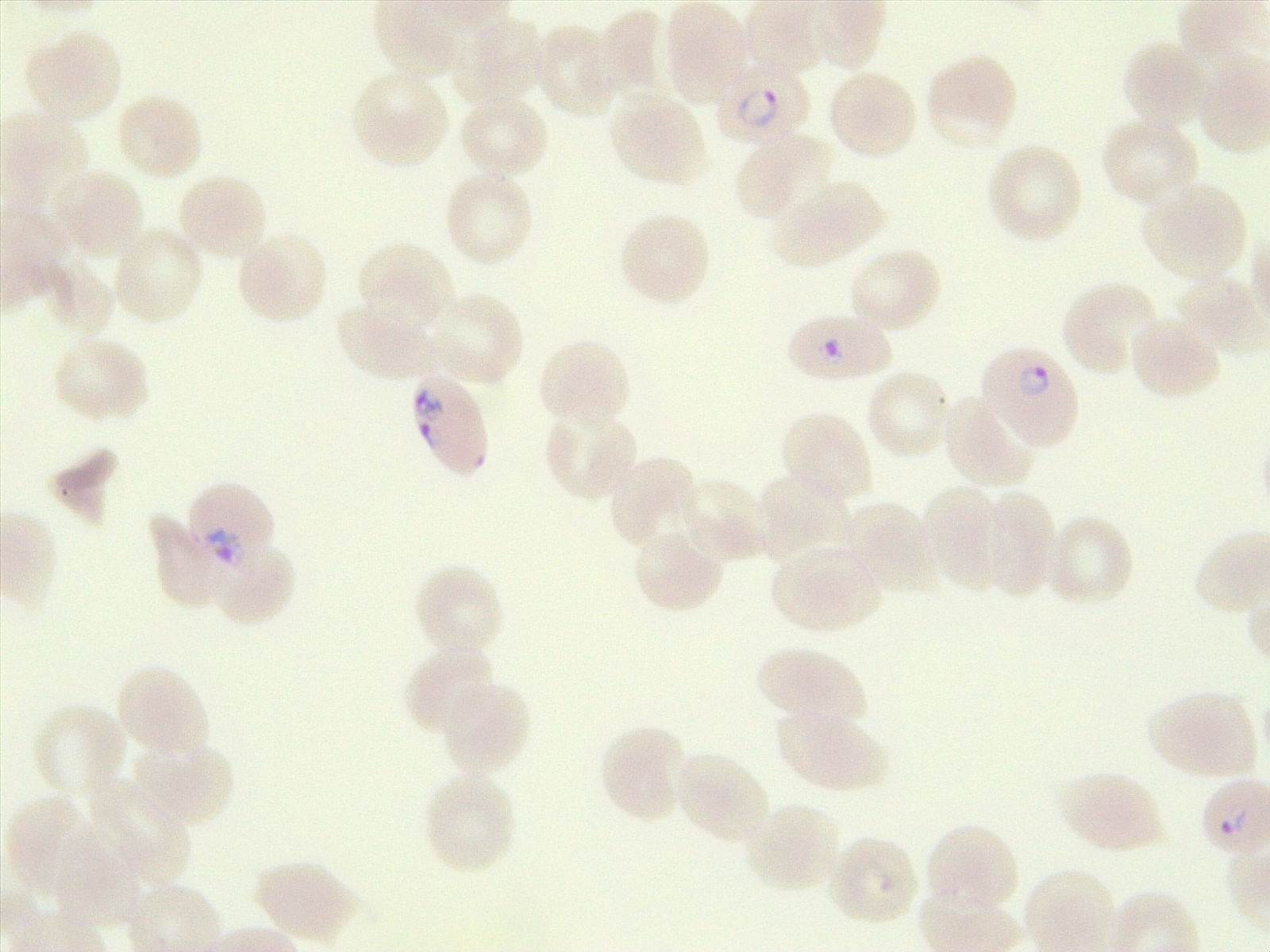

Supplement: Supplementary file 6 — Source data Fig. 4 [file 44321_2024_87_MOESM6_ESM.zip › Fig. 4/Figure 4B (contains alternative insets)/Uncropped/4B_Cam3.II_ART-link.tif]

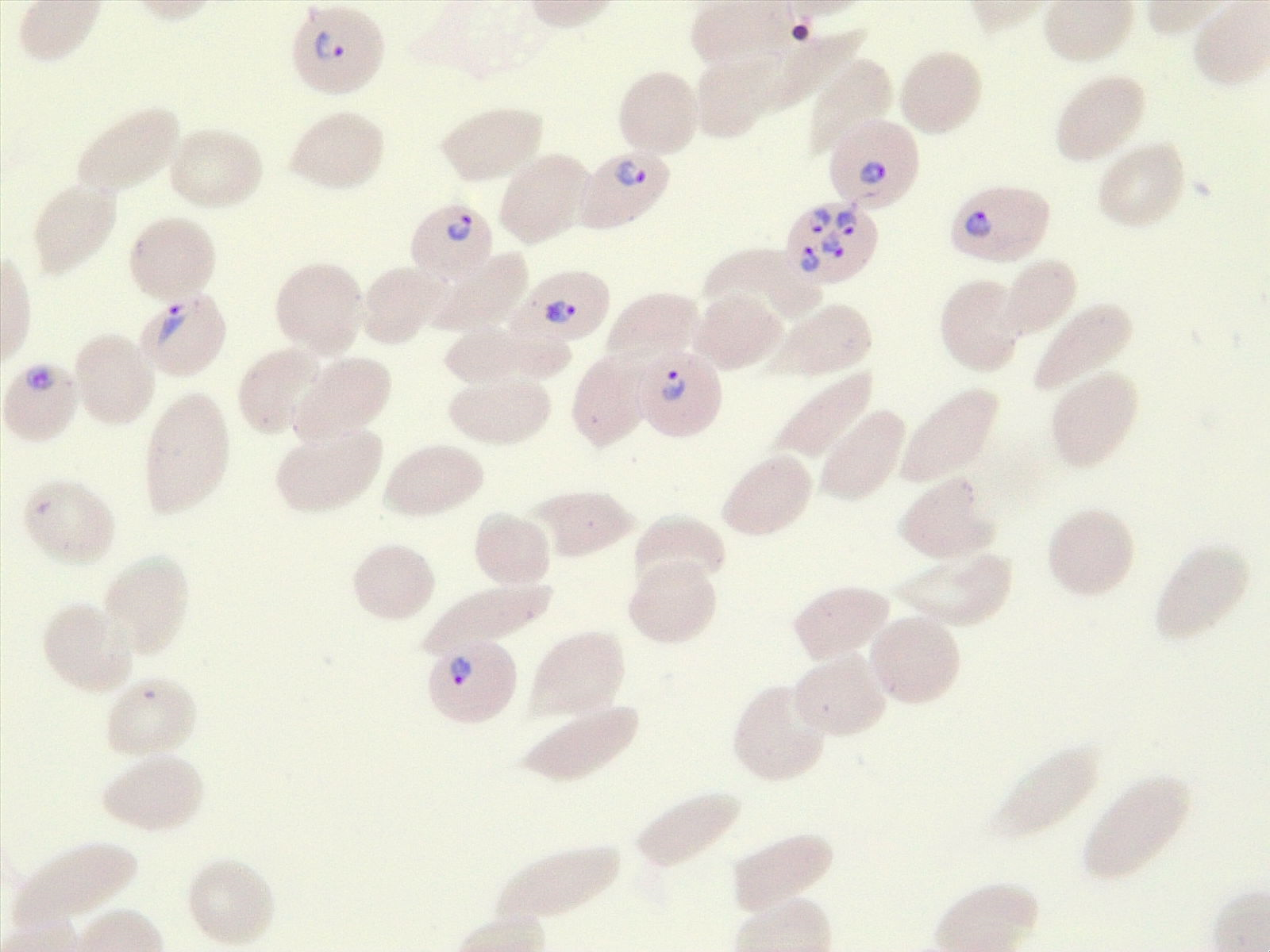

Supplement: Supplementary file 6 — Source data Fig. 4 [file 44321_2024_87_MOESM6_ESM.zip › Fig. 4/Figure 4B (contains alternative insets)/Uncropped/4B_Cam3.IIREV_DMSO.tif]

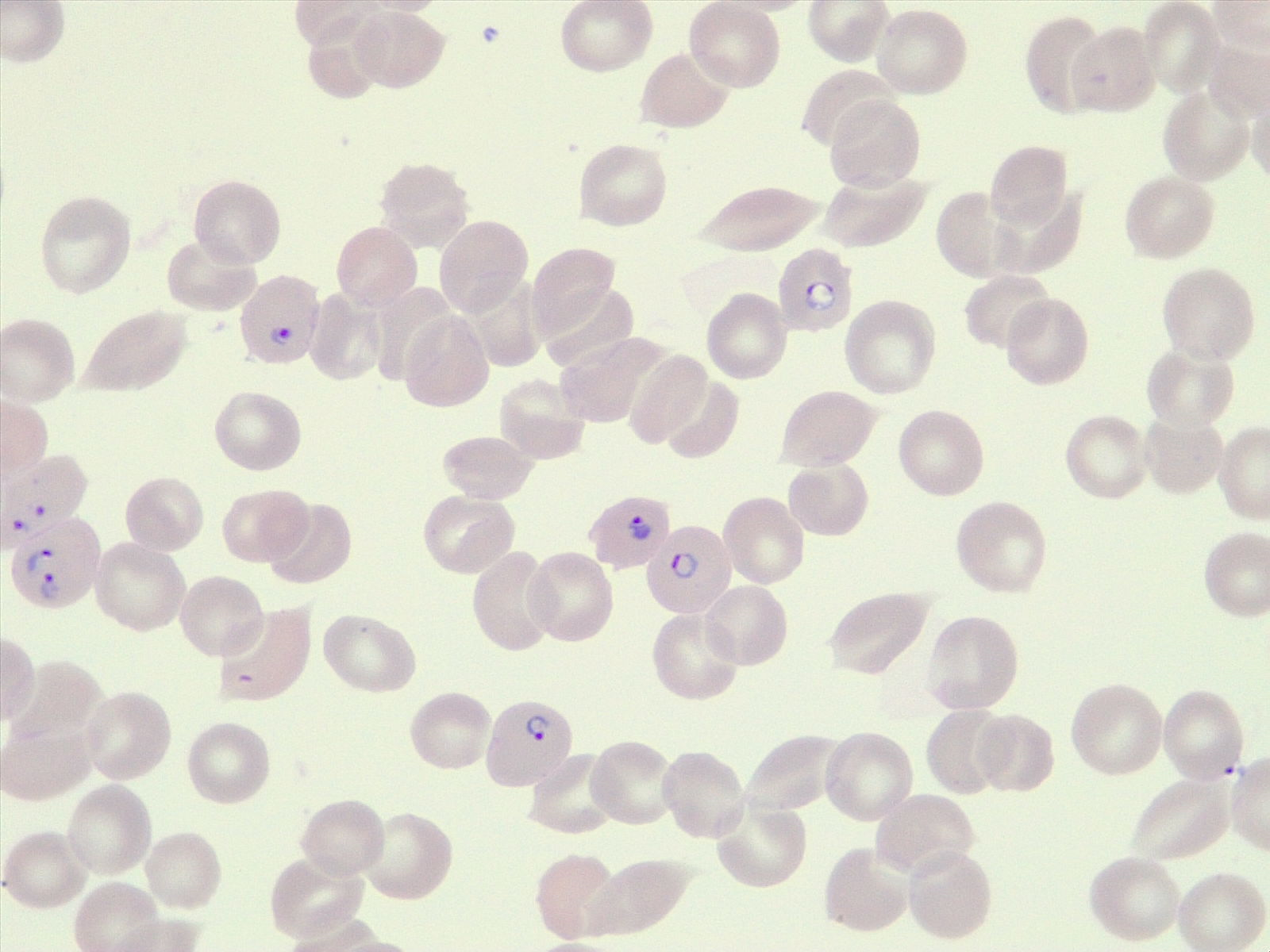

Supplement: Supplementary file 6 — Source data Fig. 4 [file 44321_2024_87_MOESM6_ESM.zip › Fig. 4/Figure 4B (contains alternative insets)/Uncropped/4B_Cam3.II_Artesunate.tif]

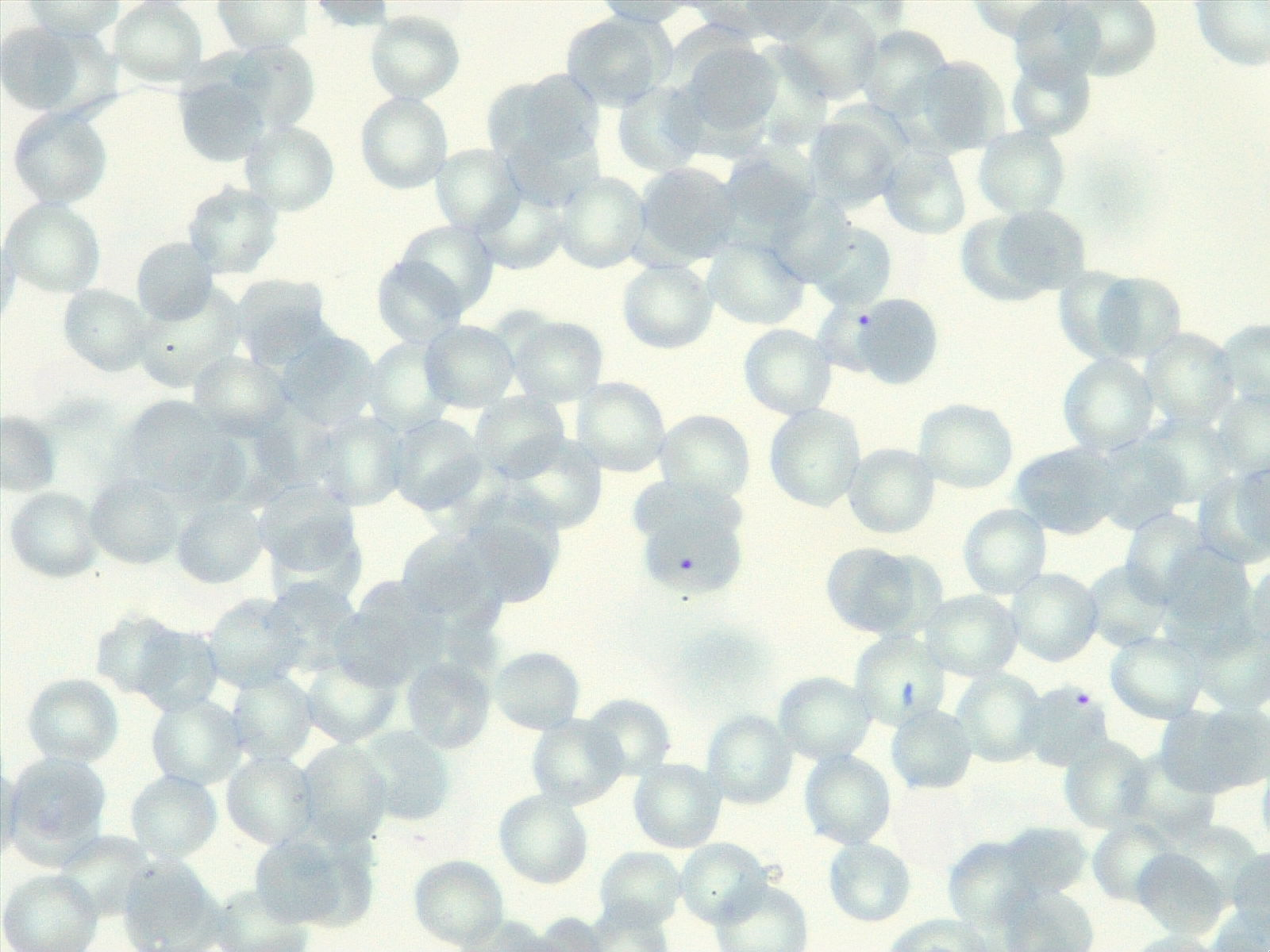

Supplement: Supplementary file 6 — Source data Fig. 4 [file 44321_2024_87_MOESM6_ESM.zip › Fig. 4/Figure 4B (contains alternative insets)/Uncropped/4B_Cam3.IIREV_C-17-ART.tif]

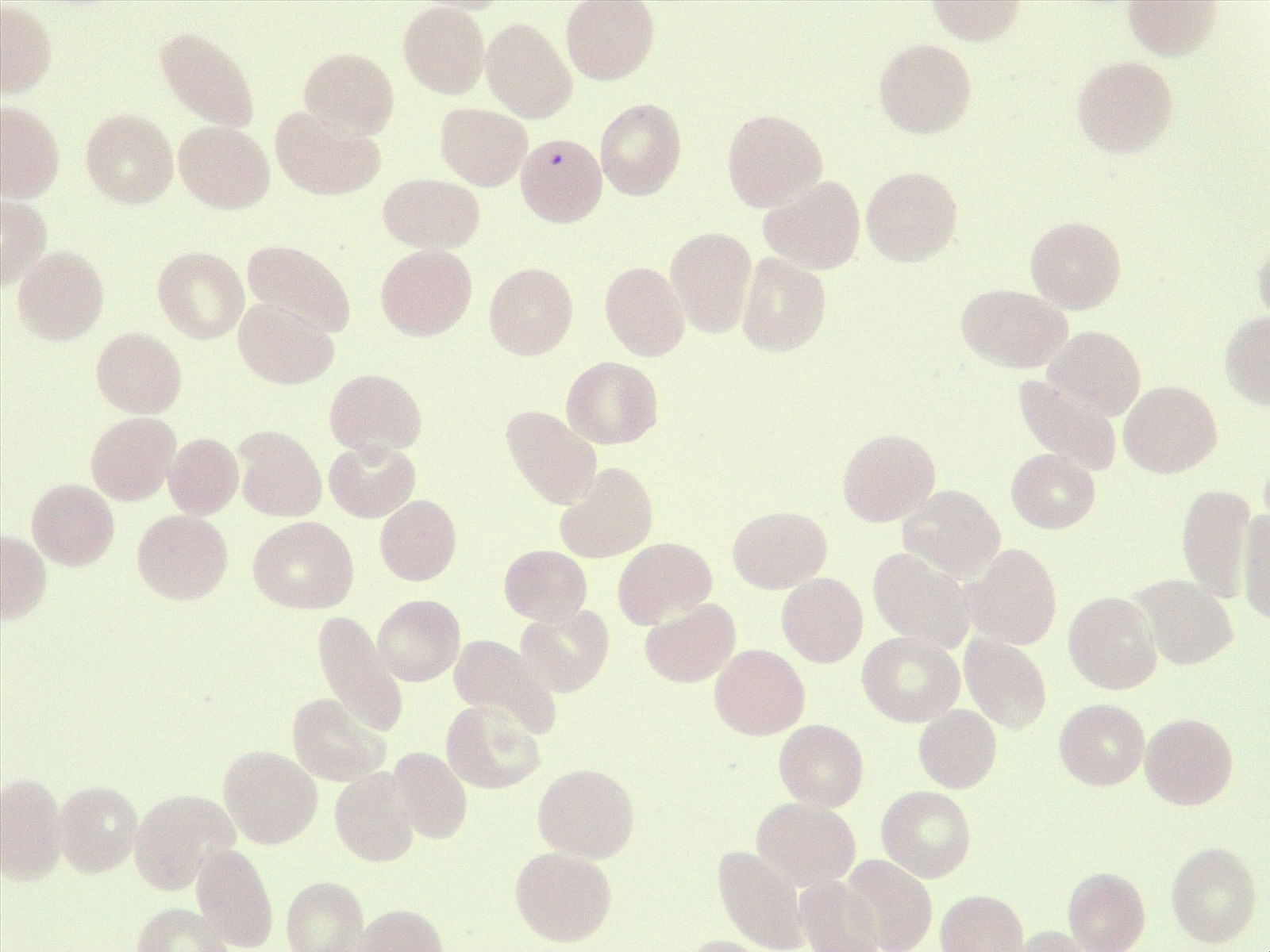

Supplement: Supplementary file 6 — Source data Fig. 4 [file 44321_2024_87_MOESM6_ESM.zip › Fig. 4/Figure 4B (contains alternative insets)/Uncropped/4B_Cam3.II_C-17-ART.tif]

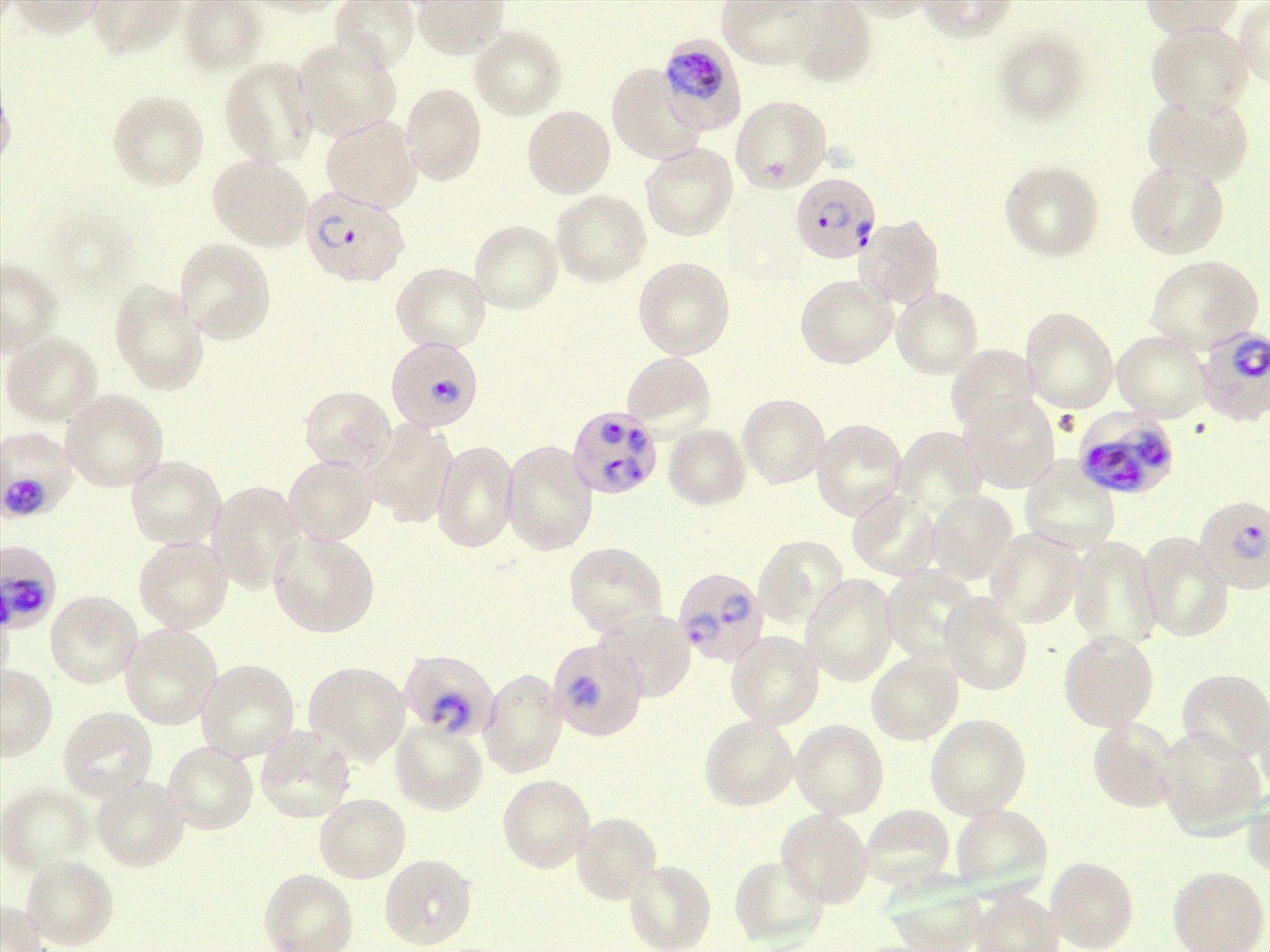

Supplement: Supplementary file 6 — Source data Fig. 4 [file 44321_2024_87_MOESM6_ESM.zip › Fig. 4/Figure 4B (contains alternative insets)/Uncropped/4B_Cam3.II_DMSO.tif]

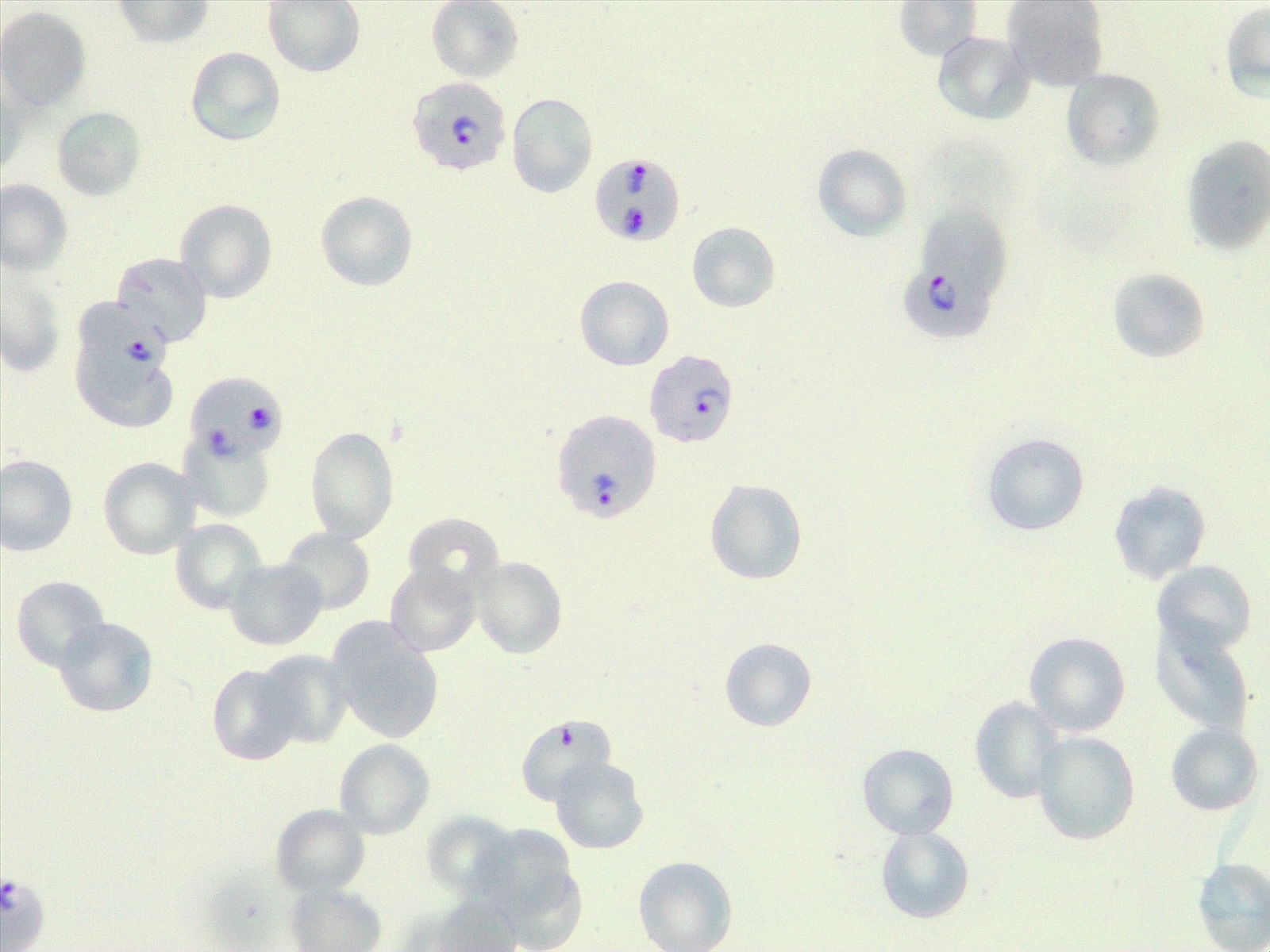

Supplement: Supplementary file 6 — Source data Fig. 4 [file 44321_2024_87_MOESM6_ESM.zip › Fig. 4/Figure 4B (contains alternative insets)/Uncropped/4B_Cam3.IIREV_C-17-link.tif]

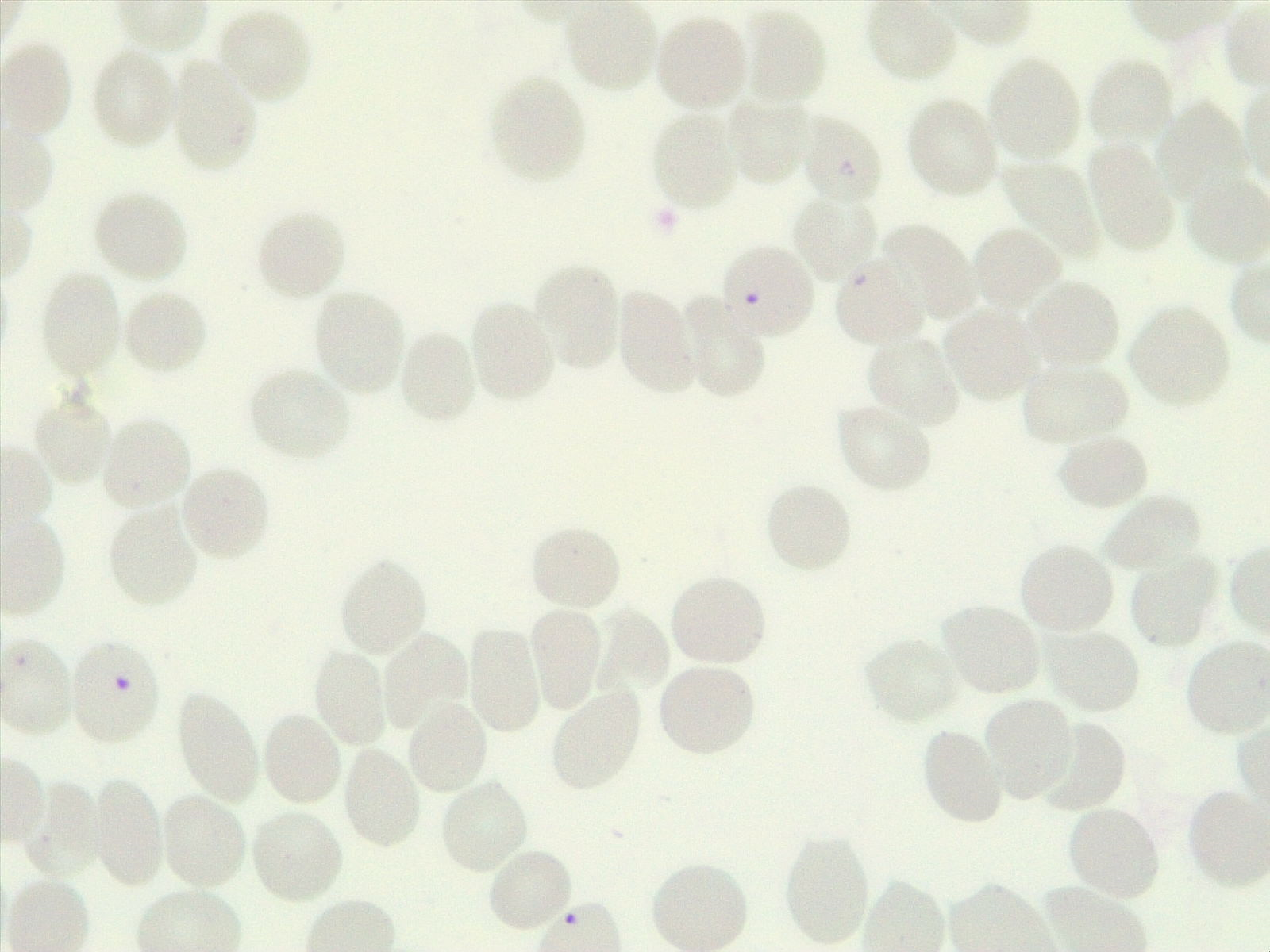

Supplement: Supplementary file 6 — Source data Fig. 4 [file 44321_2024_87_MOESM6_ESM.zip › Fig. 4/Figure 4B (contains alternative insets)/Uncropped/4B_Cam3.IIREV_Artesunate.tif]

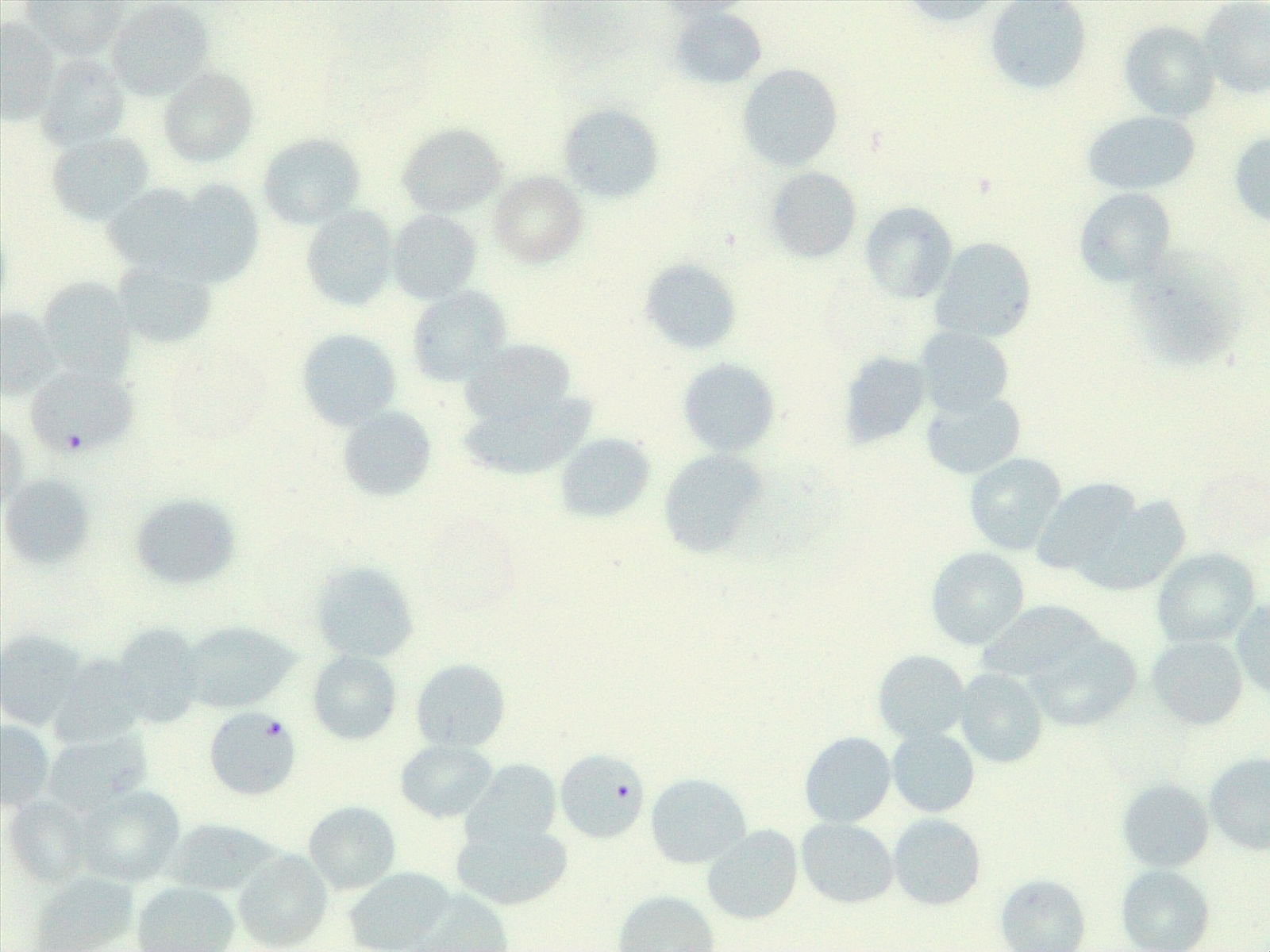

Supplement: Supplementary file 6 — Source data Fig. 4 [file 44321_2024_87_MOESM6_ESM.zip › Fig. 4/Figure 4B (contains alternative insets)/Uncropped/4B_Cam3.IIREV_ART-link.tif]

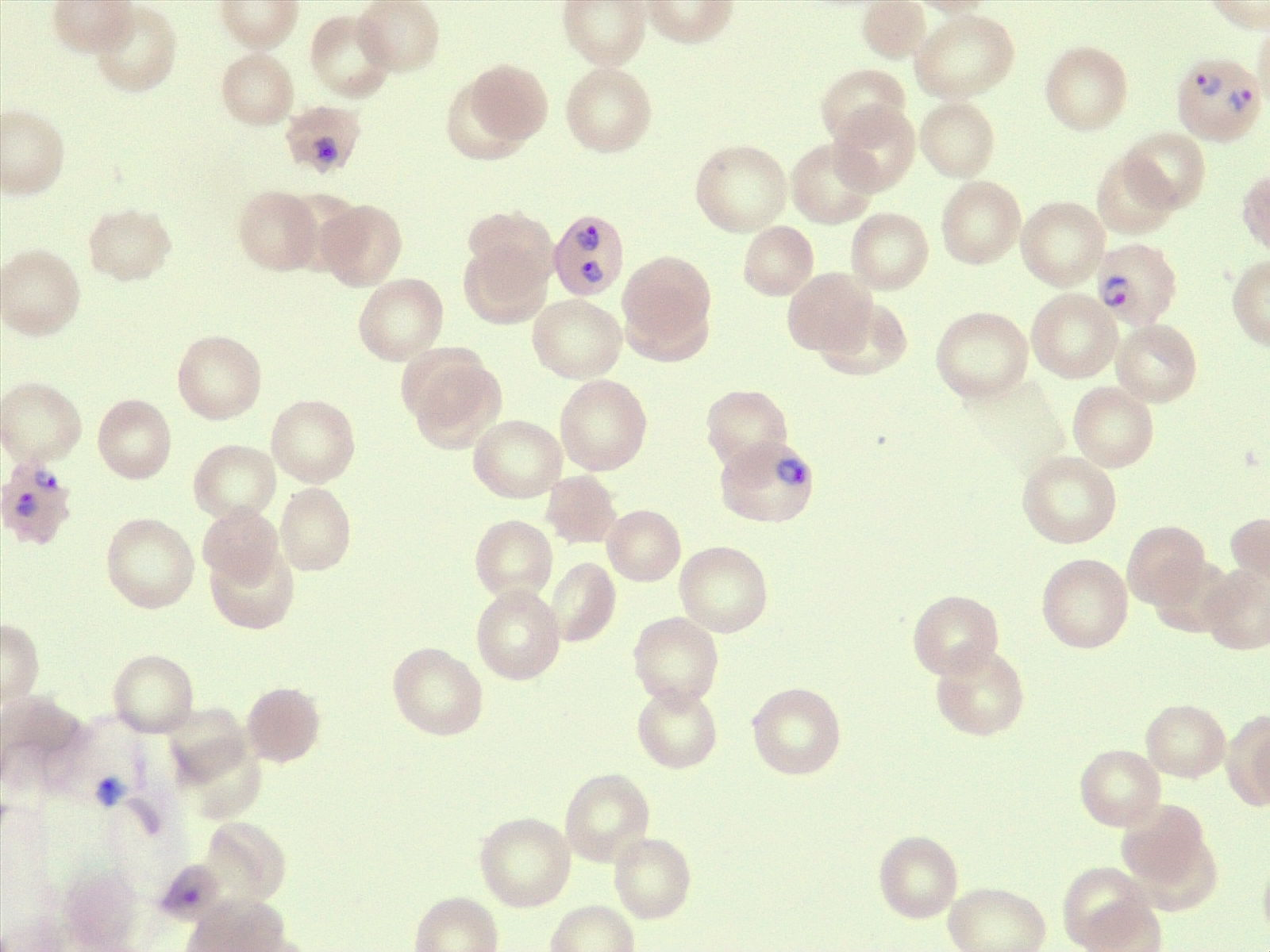

Supplement: Supplementary file 6 — Source data Fig. 4 [file 44321_2024_87_MOESM6_ESM.zip › Fig. 4/Figure 4B (contains alternative insets)/Uncropped/4B_Cam3.II_C-17-link.tif]

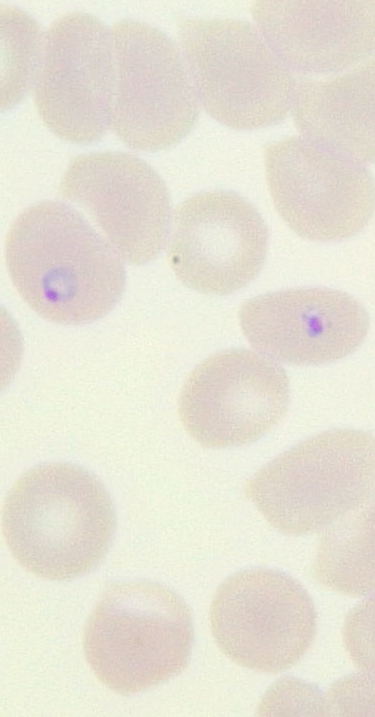

Supplement: Supplementary file 6 — Source data Fig. 4 [file 44321_2024_87_MOESM6_ESM.zip › Fig. 4/Figure 4B (contains alternative insets)/Cropped/4B_Cam3.II_ART-link.tif]

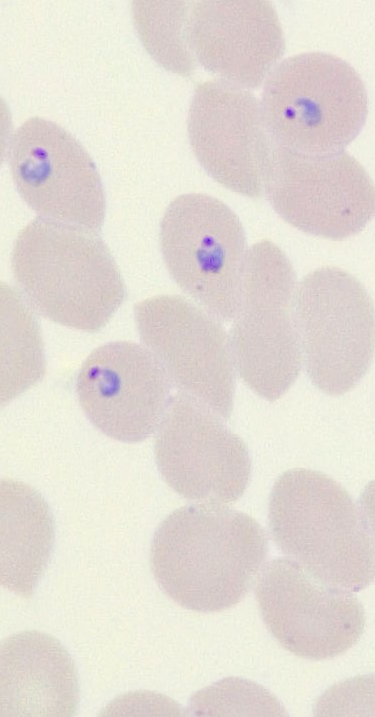

Supplement: Supplementary file 6 — Source data Fig. 4 [file 44321_2024_87_MOESM6_ESM.zip › Fig. 4/Figure 4B (contains alternative insets)/Cropped/4B_Cam3.IIREV_DMSO.tif]

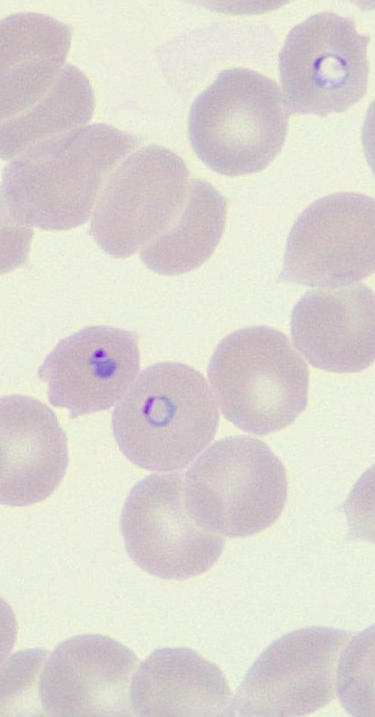

Supplement: Supplementary file 6 — Source data Fig. 4 [file 44321_2024_87_MOESM6_ESM.zip › Fig. 4/Figure 4B (contains alternative insets)/Cropped/4B_Cam3.II_Artesunate.tif]

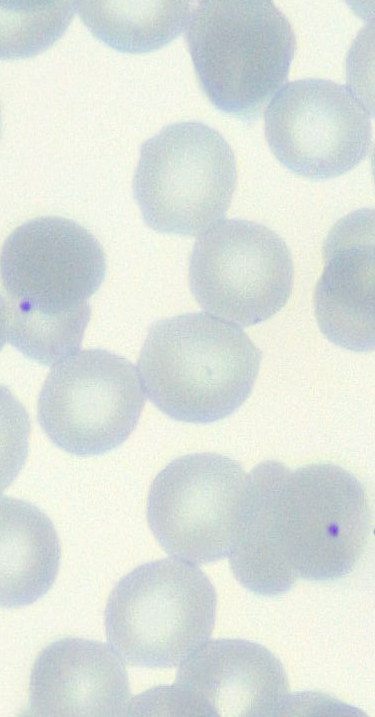

Supplement: Supplementary file 6 — Source data Fig. 4 [file 44321_2024_87_MOESM6_ESM.zip › Fig. 4/Figure 4B (contains alternative insets)/Cropped/4B_Cam3.IIREV_C-17-ART.tif]

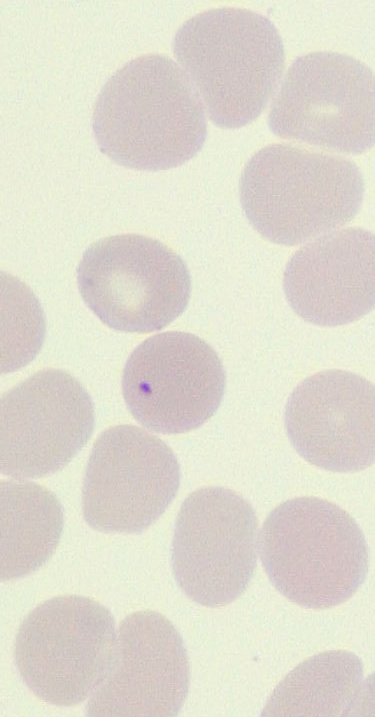

Supplement: Supplementary file 6 — Source data Fig. 4 [file 44321_2024_87_MOESM6_ESM.zip › Fig. 4/Figure 4B (contains alternative insets)/Cropped/4B_Cam3.II_C-17-ART.tif]

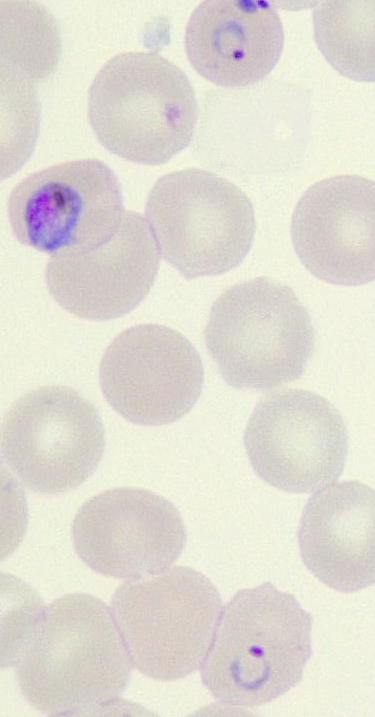

Supplement: Supplementary file 6 — Source data Fig. 4 [file 44321_2024_87_MOESM6_ESM.zip › Fig. 4/Figure 4B (contains alternative insets)/Cropped/4B_Cam3.II_DMSO.tif]

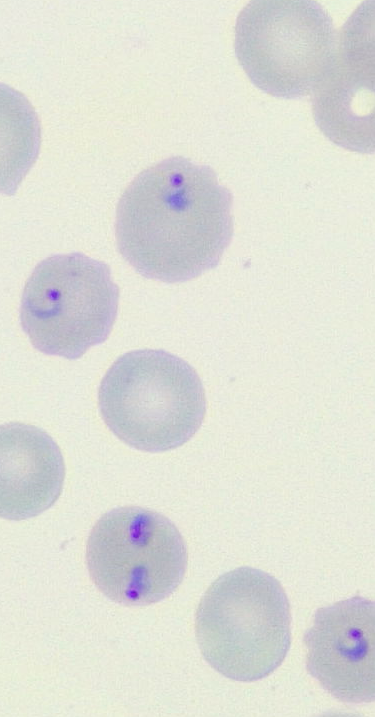

Supplement: Supplementary file 6 — Source data Fig. 4 [file 44321_2024_87_MOESM6_ESM.zip › Fig. 4/Figure 4B (contains alternative insets)/Cropped/4B_Cam3.IIREV_C-17-link.tif]

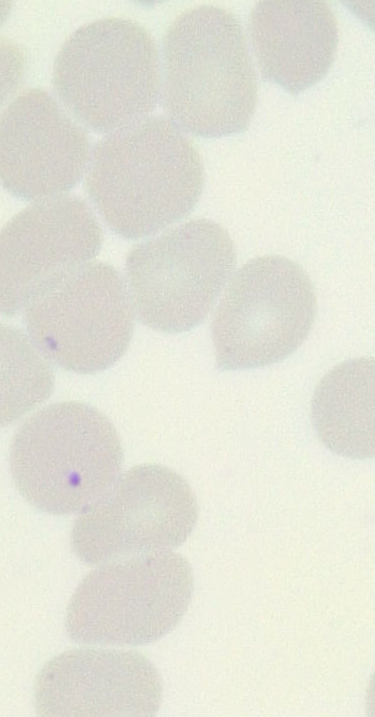

Supplement: Supplementary file 6 — Source data Fig. 4 [file 44321_2024_87_MOESM6_ESM.zip › Fig. 4/Figure 4B (contains alternative insets)/Cropped/4B_Cam3.IIREV_Artesunate.tif]

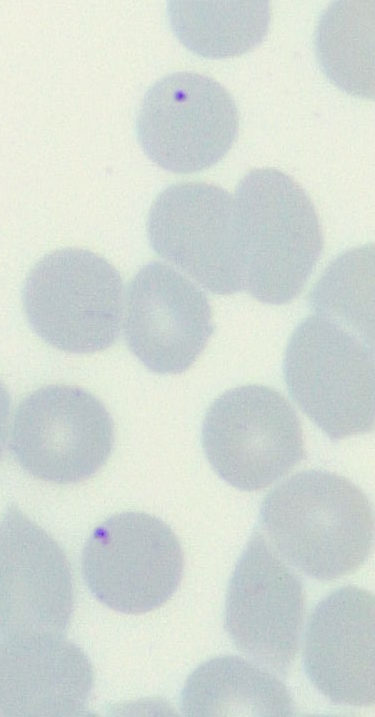

Supplement: Supplementary file 6 — Source data Fig. 4 [file 44321_2024_87_MOESM6_ESM.zip › Fig. 4/Figure 4B (contains alternative insets)/Cropped/4B_Cam3.IIREV_ART-link.tif]

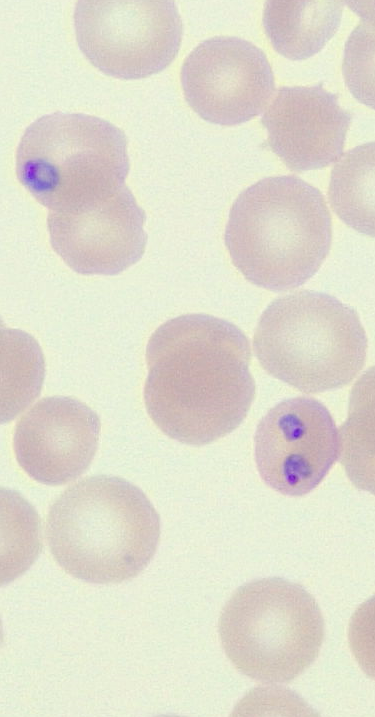

Supplement: Supplementary file 6 — Source data Fig. 4 [file 44321_2024_87_MOESM6_ESM.zip › Fig. 4/Figure 4B (contains alternative insets)/Cropped/4B_Cam3.II_C-17-link.tif]
