# Supplementary material for: Harnessing cholesterol uptake of malaria parasites for therapeutic applications
Source: EMBO Mol Med. 2024 Jun 11;16(7):4. doi: 10.1038/s44321-024-00087-1 (PMC11251039; doi:10.1038/s44321-024-00087-1)
Supplement: Supplementary file 9 — Expanded View Figures [file 44321_2024_87_MOESM9_ESM.pdf]

Expanded View Figures

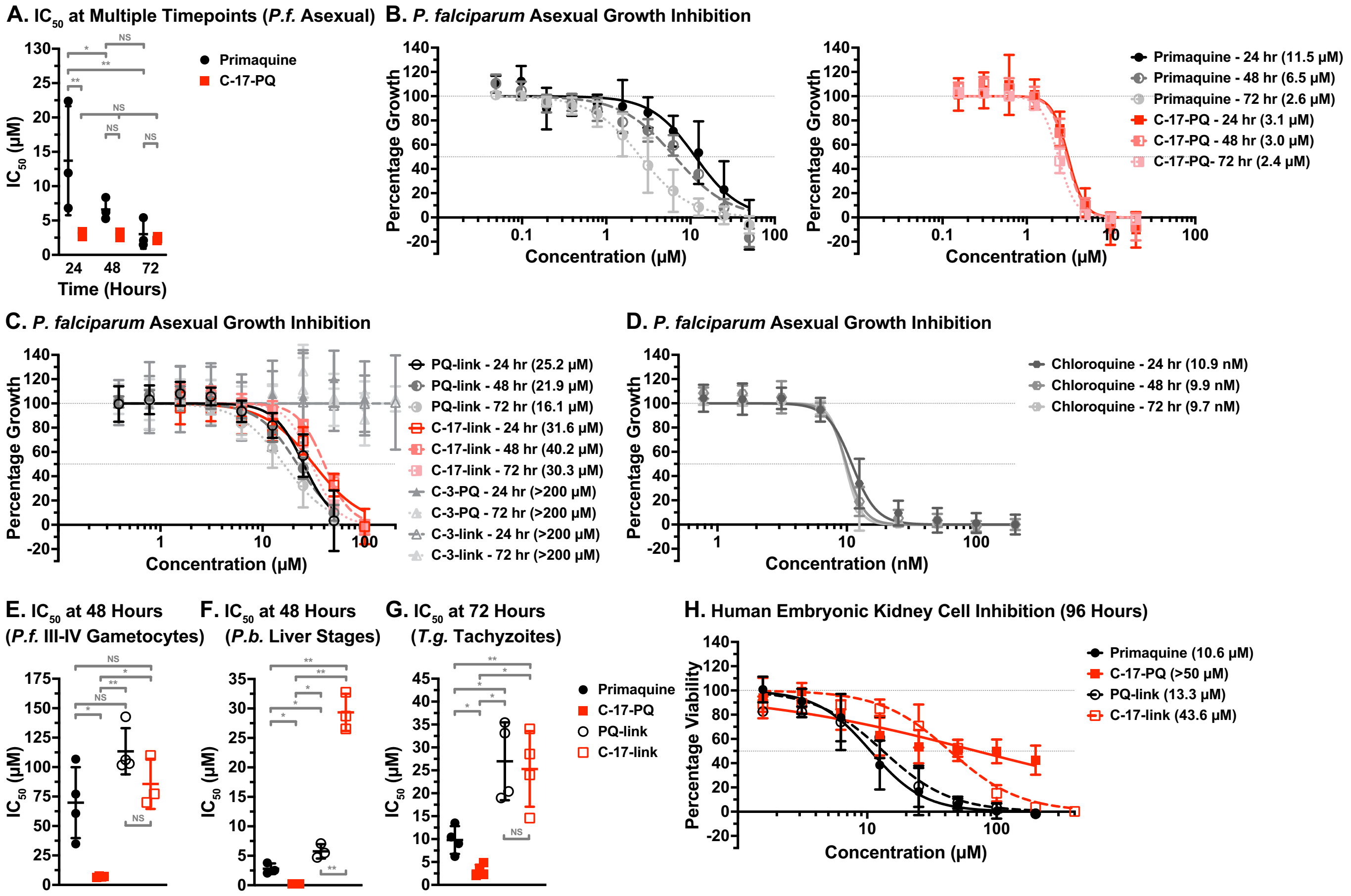

Figure EV1. Coupling of primaquine to a steroid increases its potency against apicomplexan parasites.

(**A**) Comparison of 50% inhibitory concentrations (IC_{50}) values against asexual *P. falciparum* from Fig. 2D. Individual data points are the IC_{50} values from independent experiments; centre bar is the mean value (± S.D.) of these data points. (**B–D**) Dose–response curves of (**B**) primaquine, C-17-PQ, (**C**) PQ-link, C-17-link, C-3-PQ, C-3-link, and (**D**) chloroquine against asexual P. *falciparum* growth at 24, 48 and 72 h. Shown are mean values (± S.D.). IC_{50} values are in brackets. $n = 3$ independent experiments. (**E**) Comparison of IC_{50} values against sexual *P. falciparum* gametocytes from Fig. 2C. Individual data points are the IC_{50} values from independent experiments; centre bar is the mean value (± S.D.) of these data points. $n = 4$ independent experiments. (**F**) Comparison of IC_{50} values against *P. berghei* liver stages from Fig. 3A. Individual data points are the IC_{50} values from independent experiments; centre bar is the mean value (± S.D.) of these data points. $n = 3$ independent experiments. (**G**) Comparison of IC_{50} values against *Toxoplasma gondii* from Fig. 3F. Individual data points are the IC_{50} values from independent experiments; centre bar is the mean value (± S.D.) of these data points. $n = 4$ independent experiments. (**H**) Dose–response assay (96 h) against human embryonic kidney (HEK293) cell viability incubated with primaquine, C-17-PQ, PQ-link, or C-17-link. Shown are mean values (± S.D.). IC_{50} is in brackets. $n = 3$ independent experiments. NS, not significant; *$p < 0.05$; **$p < 0.01$ (ANOVA). Source data are available online for this figure.

 © The Author(s)

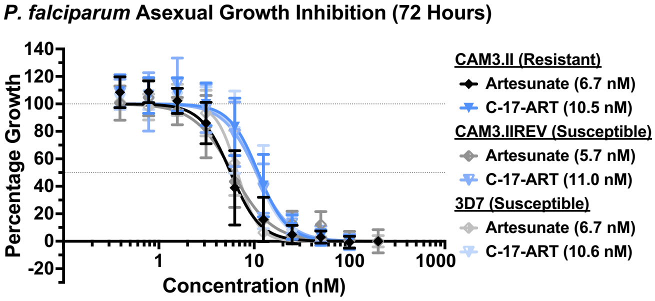

Figure EV2. Dose-response assay (72 h) of artesunate and C-17-ART against resistant (CAM3.II) and susceptible (CAM3.II REV and 3D7) asexual *P. falciparum* growth (starting at ring stages).

Shown are mean values (± S.D.). 50% inhibitory concentration (IC_{50}) is in brackets. $n = 4$ independent experiments. Source data are available online for this figure.

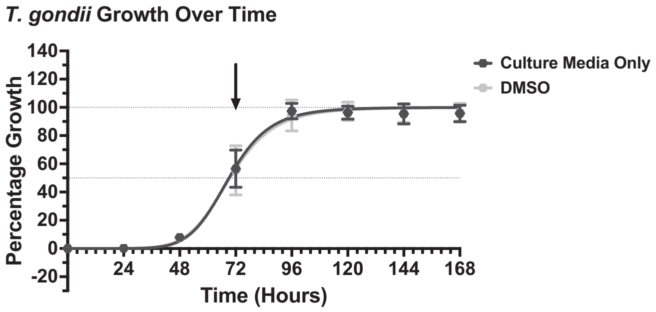

Figure EV3. Measurement of *Toxoplasma gondii* tachyzoite growth in supplemented media, or supplemented media with 0.1% (v/v) DMSO (solvent control for drugs) over one week.

Parasites were in the mid-logarithmic phase of growth at 72 h post seeding (arrow), hence this timepoint was used for the experiment outlined in Fig. 3F. Shown are mean values (± S.D.) $n = 4$ independent experiments. Source data are available online for this figure.

 © The Author(s)
